# *Verticillium dahliae* Vta3 promotes *ELV1* virulence factor gene expression in xylem sap, but tames Mtf1-mediated late stages of fungus-plant interactions and microsclerotia formation

**DOI:** 10.1371/journal.ppat.1011100

**Published:** 2023-01-30

**Authors:** Isabel Maurus, Rebekka Harting, Cornelia Herrfurth, Jessica Starke, Alexandra Nagel, Lennart Mohnike, Ying-Yu Chen, Kerstin Schmitt, Emmanouil Bastakis, Marian T. Süß, Miriam Leonard, Kai Heimel, Oliver Valerius, Ivo Feussner, James W. Kronstad, Gerhard H. Braus

**Affiliations:** 1 Department of Molecular Microbiology and Genetics, Institute of Microbiology and Genetics and Goettingen Center for Molecular Biosciences (GZMB), University of Goettingen, Goettingen, Germany; 2 Department of Plant Biochemistry and Service Unit for Metabolomics and Lipidomics, Albrecht-von-Haller-Institute for Plant Sciences and Goettingen Center for Molecular Biosciences (GZMB), University of Goettingen, Goettingen, Germany; 3 Michael Smith Laboratories, Department of Microbiology and Immunology, University of British Columbia, Vancouver, Canada; Purdue University, UNITED STATES

## Abstract

*Verticillium* transcription activator of adhesion 3 (Vta3) is required for plant root colonization and pathogenicity of the soil-borne vascular fungus *Verticillium dahliae*. RNA sequencing identified Vta3-dependent genetic networks required for growth in tomato xylem sap. Vta3 affects the expression of more than 1,000 transcripts, including candidates with predicted functions in virulence and morphogenesis such as Egh16-like virulence factor 1 (Elv1) and Master transcription factor 1 (Mtf1). The genes encoding Elv1 and Mtf1 were deleted and their functions in *V*. *dahliae* growth and virulence on tomato (*Solanum lycopersicum*) plants were investigated using genetics, plant infection experiments, gene expression studies and phytohormone analyses. Vta3 contributes to virulence by promoting *ELV1* expression, which is dispensable for vegetative growth and conidiation. Vta3 decreases disease symptoms mediated by Mtf1 in advanced stages of tomato plant colonization, while Mtf1 induces the expression of fungal effector genes and tomato pathogenesis-related protein genes. The levels of pipecolic and salicylic acids functioning in tomato defense signaling against (hemi-) biotrophic pathogens depend on the presence of *MTF1*, which promotes the formation of resting structures at the end of the infection cycle. In summary, the presence of *VTA3* alters gene expression of virulence factors and tames the Mtf1 genetic subnetwork for late stages of plant disease progression and subsequent survival of the fungus in the soil.

## Author summary

*Verticillium dahliae* causes significant economic losses to a wide range of valuable crops worldwide. The fungus can overwinter in the soil for years until it infects the roots of suitable host plants and colonizes the xylem sap, resulting in plant disease. *V*. *dahliae* needs the transcriptional regulator Vta3 for colonization of the tomato xylem sap, where it promotes gene expression of the virulence factor Elv1 but reduces expression of the transcription factor Mtf1 for fungal virulence and development. We discovered that Vta3 induced *ELV1* expression is exclusively important for virulence but not necessary for *V*. *dahliae* growth or spore production. Vta3 delays Mtf1 action, which triggers plant immune responses and is required for late stages of infection. In summary, the presence of *VTA3* alters gene expression of virulence factors and suppresses the Mtf1 subnetwork for late stages of plant disease progression. At the end of the infection cycle, Mtf1 initiates the formation of resting structures that allow the fungus to survive in the soil and later to re-infect host plants. A better understanding of the genetic networks underlying *V*. *dahliae* development and virulence is essential for developing new strategies to control growth and distribution of the fungus in fields.

## Introduction

Pathogenic fungi pose a major threat to plants worldwide. Their control in the fields remains difficult, as resistant cultivars are unavailable for many crops. *Verticillium dahliae* causes vascular wilt disease in a variety of important crop plants. Control of this soil-borne ascomycete is challenging because pesticides lose their effectiveness once the fungus is inside the plant [1–5]. Melanized fungal dormant structures (microsclerotia) can remain viable in the soil during unfavorable environmental conditions or in the absence of hosts [1,6]. Microsclerotia germinate when sensing root exudates, and hyphae grow toward the plant. *V*. *dahliae* enters through root tips, lateral root hairs, or natural root wounds before colonizing the root cortex and central cylinder [1,2,7]. Within the plant, *V*. *dahliae* forms asexual conidiospores to systematically spread throughout the host’s vasculature [7,8]. The above-ground plant tissue is supplied with important minerals and water via the xylem vessels [9–11]. However, germinating conidia can block this transport and colonize neighboring tissue, leading to plant defense responses and the occurrence of disease symptoms including wilting, stunting, chlorosis and early senescence [1,5].

The plant xylem sap is nutrient poor and unbalanced, containing plant defense proteins, hormones and low levels of amino acids and sugars [10–12]. *V*. *dahliae* requires the secretion of signaling molecules that impair the host’s defenses to live under these special nutritional conditions, compete with host cells and establish systemic infections. Sensing and adaptive mechanisms allow the fungus to respond to environmental cues such as nutrient supply or host defense molecules by altering gene expression and transport [13]. *Verticillium* can tailor its secretion patterns to adapt to different environments. The fungus produces a specific secretome pattern in xylem sap with proteins for degradation of plant material and growth under that unique condition, which differs from other growth conditions [14].

Transcriptomic responses of *V*. *dahliae* toward susceptible and tolerant olive cultivars are significantly different. Fungal genes involved in niche-adaptation, virulence and microsclerotia development were induced preferentially in the susceptible cultivar [15]. During plant infection, different transcription factors sequentially regulate the expression of host interaction-related genes. The *Verticillium* LisH domain transcription factor Som1 and the transcription activators of adhesion 2 and 3 (Vta2, Vta3) control distinct, overlapping genetic networks required for temporally sequential steps in plant root penetration and colonization [16,17]. These include shared genes such as *NLP2*, whose expression is induced for *V*. *dahliae* virulence [14,18]. Vta3 specifically links pathogenicity to development and is required for conidiation in the xylem, and microsclerotia formation for propagation in soil [17]. Transcription of *VTA3* is induced when *V*. *dahliae* was cultured in xylem sap of the host plant versus pectin-rich medium [19]. The Vta3-counterpart Crt1/RFX1 of *Saccharomyces cerevisiae* can recruit the global transcriptional co-repressors Ssn6-Tup1 and thus act as a repressor [20]. RFX1 is critical for genome integrity, hyphal growth, conidiation and virulence of the plant pathogen *Fusarium graminearum* [21]. In *Aspergillus fumigatus*, Ssn6/SsnF is linked to stress response and virulence [22].

So far, the Vta3 genetic network has been poorly characterized. Therefore, we wanted to better understand its importance for *V*. *dahliae* development and colonization of the plant xylem, including direct and indirect effects of Vta3 on the host plant and the development of the pathogen. The possible Vta3 interaction with Ssn6-Tup1 suggests that gene expression can not only be activated but also repressed by Vta3. Som1 controls transcription of *VTA3* and is requisite for root colonization earlier than Vta3 [17]. On the other hand, both Som1 and Vta3 promote the transcription of *VTA2*. The genetic networks they control are required for root colonization upstream of the network controlled by Vta2 [17]. Therefore, Vta3 may have transient functions. The plant xylem sap also serves a transient function for *V*. *dahliae*, where it feeds on plant nutrients before forming microsclerotia in the senescent plant for subsequent survival in the soil [1,2,7]. Additionally, *Verticillium* in xylem sap has been shown to communicate with its environment via specific secretome patterns [14]. We therefore focused our RNA sequencing approach on xylem sap with the goal of identifying previously unknown virulence factors that require the presence of *VTA3* and are therefore expressed in a Vta3-dependent manner. Corresponding identified genes were deleted and placed in the Vta3 control network. The phenotypes of the deletion strains were described with a focus on virulence. We were also interested in whether the transient factor Vta3, being active at advanced stages of fungal development and plant infection, modulates the expression of other regulatory genes. The function of their gene products in the life cycle of *V*. *dahliae* was investigated.

Our analyses revealed that Vta3 contributes to virulence via promoting expression of the Egh16-like virulence factor 1 (Elv1)-encoding gene *ELV1*, while restraining the Master transcription factor 1 (Mtf1)-driven genetic subnetwork for late stages of plant disease progression and development of microsclerotia as survival structures.

## Materials and methods

### Plasmid and strain construction

*ELV1* and *MTF1* deletion strains were constructed in the *Verticillium dahliae* JR2 wild-type [23] to study the cellular functions of their gene products. Gene predictions for *ELV1* (*VDAG_JR2_Chr6g05120a*) and *MTF1* (*VDAG_JR2_Chr2g08470a*) were obtained from the Ensembl Fungi database [24]. DNA fragments were amplified by PCR with Phusion (Thermo Fisher Scientific) or Q5 (New England Biolabs) DNA polymerases in Biometra thermal cyclers. Primers (S1 Table) were designed with 15 base pair (bp) overhangs homologous to the desired neighboring sequences to allow assembly using the GeneArt Seamless Cloning and Assembly Kit (Thermo Fisher Scientific). PCR products were purified using the NucleoSpin Gel and PCR Clean-up Kit (Macherey-Nagel). Plasmids (S2 Table) were transformed into *Escherichia coli* DH5α (Invitrogen Thermo Fisher Scientific) using the heat shock method [25,26], confirmed by sequencing (Microsynth Seqlab), and transformed into *Agrobacterium tumefaciens* AGL1 [27] as described [28]. *V*. *dahliae* was manipulated using *A*. *tumefaciens*-mediated transformation [17]. In-locus complemented strains were constructed in the same way to verify phenotypes of deletion strains. All strains used in this study are listed in S3 Table.

### Media and culture conditions

Lysogeny broth [29] supplemented with 100 μg ml^-1^ kanamycin (AppliChem) was used to cultivate *E*. *coli* and *A*. *tumefaciens* at 37°C and 25°C, respectively. *V*. *dahliae* was incubated at 25°C in or on simulated xylem medium (SXM), potato dextrose medium (PDM; Carl Roth) or Czapek-Dox medium (CDM) as described [30]. Conidiospores were harvested using sterile Miracloth (Calbiochem), washed and resuspended in sterile water, and counted using the Coulter Z2 Particle Count and Size Analyzer (Beckman Coulter) with Beckman Coulter Isoton II Diluent, or a Neubauer counting chamber.

### Sample preparation and transcriptome analysis

Xylem sap was extracted from six-week-old, uninfected Moneymaker tomato plants (*Solanum lycopersicum*) as described [16]. We acquired approximately 1 l pure xylem sap from 325 plants and added sterile distilled water, which accounted for 10% of the final volume. Filtered xylem sap was kept at -80°C until use. *V*. *dahliae* wild-type and the *VTA3* deletion strain were grown shaking in liquid SXM at 25°C to produce conidiospores. After harvest, 50 ml liquid SXM were inoculated with 5 × 10^7^ spores. Following cultivation for five days, mycelia were transferred to extracted tomato xylem sap and further incubated with shaking at 25°C for 8 h. For transfer, cultures were centrifuged at 2,500 rpm for 10 min and mycelia were washed with sterile water. This procedure was repeated once. Mycelia were harvested using Miracloth and ground to powder in liquid nitrogen. Fungal strains were cultivated in three independent replicates. RNA was extracted using the TRIzol/chloroform protocol [31]. DNase treatment was performed using the TURBO DNA-free Kit (Invitrogen Thermo Fisher Scientific). RNA concentrations and purity were checked with a Nanodrop spectrophotometer (Thermo Fisher Scientific).

Quality control, library preparation, sequencing and bioinformatic analysis were conducted by GENEWIZ (Azenta Life Sciences) on the HiSeq platform with 2 x 150 configuration as described [19]. Briefly, polyA selection for eukaryotic mRNA was performed. Raw reads were trimmed to remove possible adapter sequences and low-quality bases using Trimmomatic v.0.36 [32]. Trimmed reads were mapped to the *V*. *dahliae* JR2 genome obtained from Ensembl Fungi [24] using STAR aligner v.2.5.2b. Unique gene hit counts were calculated using featureCounts from the Subread package v.1.5.2. Comparison of gene expression between the three wild-type samples and the three samples of the *VTA3* deletion strain was performed using DESeq2. Sample distances were measured using expression values from each sample to identify how similar the groups are (S1 Fig). Wald tests were used to generate *P*-values and log_2_ fold changes (*VTA3* deletion strain versus wild-type). Genes with an adjusted *P*-value greater than 0.05 and log_2_(fold change) < 1 or > -1 were excluded from further analysis.

**Fig 1:**
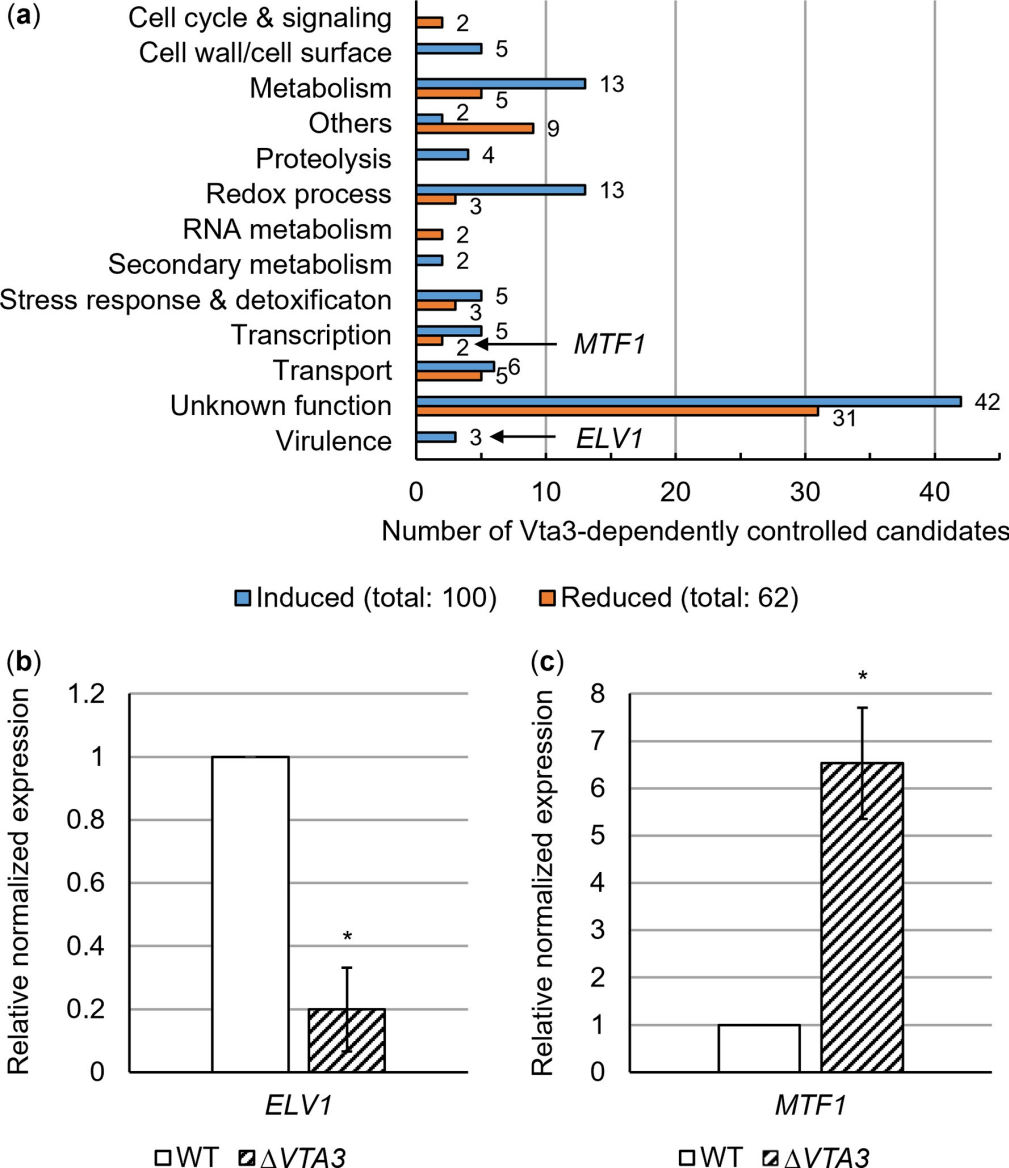
The *Verticillium dahliae* transcription factor Vta3 affects the expression of 162 fungal genes in tomato xylem sap including the Egh16-like virulence factor-encoding gene *ELV1* and the Master transcription factor-encoding gene *MTF1*. Equal numbers of spores of *V*. *dahliae* JR2 wild-type (WT) or *VTA3* deletion strain (Δ*VTA3*) were used to inoculate liquid simulated xylem medium and incubated for five days with shaking. The mycelia were then transferred to extracted tomato xylem sap and incubated for a further 8 h. (a) The transcriptomes of three biological replicates per fungal strain were analyzed by RNA sequencing. After applying a log_2_(fold change) of ≥ 2, transcript levels of 100 genes were significantly higher in the presence of *VTA3* (induced; in blue), whereas expression of 62 genes was reduced (in orange). The products of the differentially expressed genes are involved in various processes including metabolism, redox processes, stress response, transcription, transport, virulence and unknown functions. Putative functions were assigned based on conserved domains identified by searches with InterPro and BLAST. Details are given in S7 Table (Vta3-dependently induced candidates) and S8 Table (Vta3-dependently reduced candidates). (b, c) *ELV1* and *MTF1* expression levels were analyzed by reverse transcription-quantitative PCR. The transcript levels of references *H2A* and *EIF2B* were used for normalization and gene expression levels in wild-type were set to one. Mean values ± SE of the mean from three independent experiments are shown. Significance was calculated using *t*-tests. (b) The presence of *VTA3* leads to a five-fold induction of *ELV1* expression (*, *P* < 0.05). (c) *MTF1* expression was reduced six-fold (*, *P* < 0.05) in the presence of *VTA3*.

All remaining genes were considered significantly differentially expressed and analyzed for functional enrichment (S4–S6 Tables) with the FungiFun2 web tool [33] using corresponding VdLs.17 identifiers. The web tool produces overlaps between categories thus some genes appear in more than one category. Transcripts with a log_2_(fold change) ≤ -2 were regarded as most induced (S7 Table), those with a log_2_(fold change) ≥ 2 as most reduced by Vta3 (S8 Table). These transcripts were further investigated separately (Fig 1A) using Ensembl Fungi [24], InterPro [34], and BLAST with NCBI [35] and FungiDB [36].

### RNA extraction, cDNA synthesis and quantification of gene expression

Fungal strains were cultured as described above and RNAs were extracted from ground mycelia using TRIzol [31]. RNA from tomato hypocotyls was extracted in the same manner at 21 days post inoculation (dpi). QuantiTect Reverse Transcription Kit (Qiagen) was used for cDNA synthesis using 0.8 μg RNA. Transcript levels were analyzed in duplicate or triplicate (*n* = 1) using MESA GREEN qPCR MasterMix Plus for SYBR Assay (Eurogentec) in a CFX Connect Real Time PCR Detection System (Bio-Rad Laboratories). Primers used for quantitative PCR (qPCR) are listed in S9 Table. Expression levels of fungal genes were quantified relative to references histone *H2A* and *EIF2B*. For plant genes, tomato *αTUB* and elongation factor *EF1α* were used as references. The $2^{{-\Delta \Delta C}_{T}}$ method [37] was applied and wild-type expression was set to one. Means of two or three independent experiments ± SE of the mean are presented.

### Plasmid and strain construction of *ELV1* deletion and complementation strains

The 1,277 bp *ELV1* open reading frame (ORF) was replaced by a 2,194 bp nourseothricin resistance marker under control of a *gpdA* promoter and a *trpC* terminator amplified from pME4815 [14] using the primers ML8 and ML9. The primers IM32 and IM105 were used to amplify the 800 bp 5’ flanking region from wild-type genomic DNA and the 1,000 bp 3’ flanking region was amplified using the primers IM34 and IM35. All fragments were inserted into the *Eco*RV and *Stu*I-treated 6,804 bp pME4564 [14] backbone resulting in pME5482. The *V*. *dahliae* wild-type was transformed with this plasmid resulting in the *ELV1* deletion strains VGB670 and VGB671.

For the *ELV1* complementation strain, the 5’ flanking region, the backbone of pME4564 [14] and the 3’ flanking region were amplified from the plasmid pME5482 using the primers IM34 and IM85. The *ELV1* gene was obtained by amplification from wild-type genomic DNA using the primers IM86 and IM143. A 3,942 bp hygromycin B resistance marker under control of a *gpdA* promoter and a *trpC* terminator was amplified from the plasmid pPK2 [38] using the primers ML8 and RO3. All fragments were ligated and the resulting plasmid pME5483 was used to transform the *ELV1* deletion strain VGB670. The *ELV1* complementation strain was named VGB694. Correct integration of deletion and complementation constructs into *V*. *dahliae* was verified by Southern hybridization using *Sal*I restriction and the 5’ flanking region as probe (S2 Fig).

### Plasmid and strain construction of *MTF1* deletion and complementation strains

The 1,023 bp *MTF1* ORF was replaced by homologous recombination with a 2,643 bp hygromycin B resistance marker cassette amplified from the plasmid pPK2 [38] using the primers ML8 and ML9. The primers IM40 and IM41 were used to amplify the 650 bp 5’ flanking region from genomic DNA of the wild-type and the 1,300 bp 3’ flanking region was amplified using primers IM42 and IM43. All fragments were inserted into the *Eco*RV and *Stu*I-treated pME4564 [14] backbone. The resulting plasmid pME5480 was used to transform the *V*. *dahliae* JR2 wild-type by *A*. *tumefaciens*-mediated transformation resulting in the *MTF1* deletion strains VGB575 and VGB576. For visualization of fungal hyphae on plant roots, the *MTF1* deletion strain VGB575 was transformed with the plasmid pME4819 [39]. The μ-Slide 8 well microscopy chambers (Ibidi) were used to screen the transformants for green fluorescent hyphae. The *MTF1* deletion strains harboring ectopically integrated *GFP* were named VGB625 and VGB626.

For the *MTF1* complementation strains, the 5’ flanking region together with the *MTF1* gene were amplified using the primers IM40 and IM84. This fragment was inserted into the *Eco*RV and *Stu*I-treated pME4564 [14] backbone together with a nourseothricin resistance marker cassette and the 1,300 bp 3’ flanking region. The resulting plasmid pME5481 was used for transformation of the *MTF1* deletion strain VGB575 to obtain the *MTF1* complementation strains VGB635 and VGB636. For the *GFP-MTF1* strain, the nourseothricin resistance marker cassette, the 3’ flanking region, the backbone of pME4564 [14] and the 5’ flanking region were amplified from pME5481 with primers ML5 and IM92. *GFP* including a linker was amplified with primers ZQY10 and ZQY11 from pGreen2 [16]. Primers IM84 and IM91 were used to amplify the *MTF1* gene from wild-type genomic DNA. These fragments were ligated to yield the plasmid pME5510 which was used to transform the *MTF1* deletion strain VGB575. The resulting *GFP-MTF1* expressing strain was named VGB650. Correct integration of deletion and complementation constructs into *V*. *dahliae* was verified by Southern hybridization using *Sal*I restriction and the 5’ flanking region as probe (S3 Fig).

### Genomic DNA isolation and Southern hybridization

Genomic DNA extraction and Southern hybridization were performed as previously described [14,17]. Briefly, mycelia were harvested using Miracloth filters and homogenized into powder in liquid nitrogen. Genomic DNA was isolated with phenol from mycelium grown shaking at 25°C in liquid PDM. Amersham AlkPhos Direct Labelling and CDP-Star detection reagents (GE Healthcare) were used for Southern hybridization.

### Protein extraction and western experiments

For detection of GFP-fused proteins, 5 × 10^7^ freshly harvested spores were inoculated into liquid PDM, SXM, CDM, extracted tomato xylem sap or onto 30 ml solid SXM plates covered with nylon membrane (GE Healthcare) and incubated for indicated times. For xylem sap, a five-day preculture was performed in SXM, after which the mycelia were transferred to xylem sap and incubated for an additional 8 h. After harvesting, the mycelium was ground to powder in liquid nitrogen. Protein extracts were obtained using B* buffer, and protein concentrations were determined as previously described [39]. Western experiments were performed with 80 to 160 μg protein extracts according to the protocol described [14,17]. Prior to blocking, Ponceau S staining was applied as a loading control [39]. Membranes were incubated with a monoclonal mouse α-GFP antibody (Santa Cruz Biotechnology) and a secondary horseradish peroxidase-coupled goat α-mouse antibody (Jackson ImmunoResearch). Signals were visualized on Amersham Hyperfilm ECL films (GE Healthcare) developed with Optimax film processor (Protec).

### *In-vitro* protein pull-down and sample preparation for liquid chromatography/mass spectrometry

Liquid PDM (500 ml) was inoculated with 5 × 10^8^ freshly harvested spores and cultured for five days at 25°C with shaking. Proteins were extracted from the *V*. *dahliae* JR2 wild-type, the wild-type strain overexpressing ectopically integrated *GFP* and the *VTA3-GFP* expressing strain. The experiment was performed as previously described [31] with the following modifications: 15 μl of GFP-Trap Agarose beads were used. The three eluates were collected in one tube. Sediments were dissolved in 40 μl of sample buffer by shaking for 5 min and incubation in an ultrasonic bath for 3 min. The peptides were purified using StageTips [40,41]. Briefly, StageTips were equilibrated in four steps with (I) 100 μl methanol/0.1% formic acid, (II) 100 μl 70% acetonitrile/0.1% formic acid, (III) 100 μl water/0.1% formic acid and (IV) 100 μl water/0.1% formic acid. Samples were divided onto two StageTips and incubated on the columns for 5 min. This step was repeated after centrifugation at 4,000 rpm for 5 min. The columns were washed twice with 200 μl of water/0.1% formic acid. Elution was performed by adding 60 μl of 70% acetonitrile/0.1% formic acid, and the two parts of each sample were combined. Peptide samples were dried and liquid chromatography/mass spectrometry (LC/MS) analysis of the peptides was performed as described [31]. MaxQuant 1.6.10.43 [42] and Perseus 1.6.0.7 [43] were used to analyze MS raw data. Data were processed as described [31]. Missing values were replaced four times from the normal distribution. The *VTA3-GFP* expressing strain was used as the first group and the wild-type as the second group. Mass spectrometry proteomics data have been deposited to the ProteomeXchange Consortium via the PRIDE [44] partner repository with the dataset identifier PXD039123.

### Phenotypical analyses

Freshly harvested spores were adjusted to 5 × 10^6^ spores ml^-1^ and 10 μl were spotted onto indicated media plates. PDM, SXM, CDM, CDM with 3% cellulose as an alternative carbon source and CDM supplemented with 0.004% SDS as a stress inducing agent were used. Two independent transformants per genotype were compared with wild-type. After 10 days of incubation at 25°C, growth and microsclerotia production of the strains were assessed by binocular (SZX12-ILLB2-200, illuminated with the KL1500-LCD light source, Olympus) and light microscopy (Axiolab, Zeiss) (equipped with SC30 cameras, Olympus) with cellSens Dimension software (Olympus).

### Quantification of microsclerotia and conidia formation

The formation of microsclerotia in fungal colonies was assessed by measuring the brightness factor with the ROI Manager of ImageJ software [45] as previously described [31,39]. CDM plates containing cellulose were inoculated with 50,000 freshly harvested spores in triplicate (*n* = 1) and incubated at 25°C for 10 days. In three independent experiments, two individual transformants of the deletion and complementation strains were compared with two independent wild-type cultures each (*n* = 6). Means ± SD are shown and statistical significance was calculated using *t*-tests.

Quantification of conidia production was performed as previously described [31,39] with the following modifications: Spores were incubated for five days and each strain was inoculated in three to four technical replicates (*n* = 1). In each experiment, two independent transformants of the deletion or complementation strains were compared with two independent wild-type cultures. Significance was calculated using *t*-tests.

### *Arabidopsis thaliana* root infection

The assay for colonization of *A*. *thaliana* Col-0 roots by the indicated *V*. *dahliae* strains was performed as previously described [17,30]. Fluorescence micrographs were taken at two and five days post inoculation (dpi) with a 20×/0.5 air objective of the Axio Observer Z1 system (Zeiss) with Laser Lunch System (Model 3iL32, Intelligent Imaging Innovations), QuantEM:512SC camera (Photometrics) and the Slide Book 6.0 imaging software (Intelligent Imaging Innovations). Two independent experiments with strains constitutively expressing ectopically integrated *GFP* were performed. In each of them, two individual Δ*MTF1* strains (Δ*MTF1 GFP* OE; VGB625, VGB626) and two individual Δ*VTA3* strains (Δ*VTA3 GFP* OE; VGB184, VGB185) were compared with respective wild-type controls (WT *GFP* OE; VGB45, VGB392). Quantification of fungal root colonization was performed at 5 dpi. For each root, 10 to 15 stacks consisting of several individual pictures were acquired at randomly selected sections. Roots of two plants per treatment were considered as one biological replicate. The green fluorescence of *GFP*-expressing fungal hyphae was quantified relative to the root area using the mask statistics feature of the Slidebook 6.0 software. The means of four biological replicates ± SD are presented and a *t*-test was used for statistical analysis.

### Pathogenicity assay on tomato plants

Pathogenicity assays were performed as previously described [14,30]. In brief, *S*. *lycopersicum* (‘Moneymaker’, Kiepenkerl Bruno Nebelung) seeds were surface sterilized with 70% (v/v) Ethanol, 0.05% Tween 20. We wounded the roots of 10-day-old seedlings and inoculated them by incubation in 50 ml of 10^7^ spores ml^-1^ or water (mock) for 40 min under constant agitation. Additionally, 3 × 10^7^ spores or 3 ml water (mock) were added to the seedlings in pots containing a sand/soil mixture. Plants were incubated for 21 days in a BrightBoy GroBank (CLF PlantClimatics). Plant weight, height and longest leaf length were measured and calculated into a disease score ranking relative to the means of mock-inoculated plants (set to 100%). Values above 80% were classified as ‘healthy’, 60–80% as ‘mild symptoms’, 40–60% as ‘strong symptoms’ and below 40% as ‘very strong symptoms’. The disease scores of each plant are visualized in stack diagrams relative to the total amount of treated plants. Statistical significance was calculated using two-tailed Mann-Whitney *U* tests [46]. We also observed the discoloration of the tomato hypocotyls and tested the plants for fungal outgrowth from surface-sterilized stem sections.

*V*. *dahliae* DNA was quantified at 21 dpi in hypocotyls using primers OLG70/OLG71 [7] to amplify a specific rDNA fragment (parts of the *5*.*8S rRNA* and ITS2) relative to the tomato elongation factor-encoding gene *EF1α* [47]. For this purpose, genomic DNA was isolated from 13 to 15 hypocotyls per treatment using the NucleoSpin Plant II Kit (Macherey-Nagel). We tested 10 ng DNA in technical triplicates using SsoAdvanced Universal SYBR Green Supermix (Bio-Rad Laboratories). The $2^{{-\Delta \Delta C}_{T}}$ method [37] was applied and wild-type was used for normalization. Means of five to six biological replicates ± SE of the mean are presented and a *t*-test was used for statistical analysis.

### Extraction of plant hormones and relative signal determination

Leaves from tomato plant infection experiments were harvested at 21 dpi, frozen and ground in liquid nitrogen. Phytohormone extraction with 80% methanol was performed on approximately 50 mg (fresh weight) ground material [48]. Pipecolic acid (Pip) and salicylic acid (SA) were reversed phase-separated using an ACQUITY UPLC system (Waters) and analyzed by nanoelectrospray ionization (TriVersa Nanomate, Advion BioSciences) coupled with an AB Sciex 4000 QTRAP tandem mass spectrometer (AB Sciex) employed in scheduled multiple reaction monitoring mode as described [49]. The reversed phase separation was achieved by UPLC using an ACQUITY UPLC HSS T3 column (100 mm x 1 mm, 1.8 μm; Waters). Solvent A and B were water and acetonitrile/water (90:10, v/v), respectively, both containing 0.3 mmol l^-1^ NH_4_HCOO (adjusted to pH 3.5 with formic acid). The flow rate was 0.16 ml min^-1^ and the separation temperature was constant at 40°C. For Pip, the elution was performed isocratically for 1 min at 1% solution B, followed by a linear increase to 95% solution B in 4.5 min, this condition was held for 2.5 min. The column was re-equilibrated for start conditions in 6 min. For SA, the elution was performed isocratically for 0.5 min at 10% solution B, followed by a linear increase to 40% solution B in 1.5 min, this condition was held for 2 min, followed by a linear increase to 95% solution B in 1 min, this condition was held for 2.5 min. The column was re-equilibrated for start conditions in 3 min. For nanoelectrospray ionization-tandem mass spectrometric analysis, Pip was ionized in positive mode and SA in negative mode. The mass transitions were as follows: 130/84 (declustering potential 90 V, entrance potential 8 V, collision energy 22 V) for Pip and 137/93 (declustering potential -25 V, entrance potential -6 V, collision energy -20 V) for SA. The relative signal area per fresh weight was calculated and wild-type was set to one. Means of 15 plants per treatment from two independent experiments ± SD are presented.

### Bioinformatics methods

The Ensembl Fungi database [24] was used for annotation of *V*. *dahliae* genes. The FIMO web tool [50] was used for performing promoter analyses. Protein sequences were analyzed via the InterPro website [34]. The eventual presence of signal peptides in protein sequences was analyzed using SignalP 6.0 [51]. cNLS Mapper [52] was used to predict nuclear localization signals in the entire protein sequence. Protein sequences of other fungi mentioned in this study were obtained from NCBI [35]. Multiple sequence alignments were performed using the MegAlign Pro software (DNASTAR, version 16) with the MUSCLE algorithm for uncorrected pairwise distance alignments. Sequences of tomato genes were obtained from Ensembl Plants [24]. Functional enrichment analysis of differentially expressed genes was performed using the FungiFun2 web tool [33]. Significance (ns, not significant; *, *P* < 0.05; **, *P* < 0.01; ***, *P* < 0.001; ****, *P* < 0.0001) was calculated by independent two-sample *t*-tests using the iCalcu website (https://www.icalcu.com/stat/two-sample-t-test-calculator.html). Student’s *t*-test was used for equal variances, while Welch’s *t*-test was applied in case of unequal variances. The two-tailed Mann-Whitney *U* test [46] was performed for statistical analysis of tomato plant infections.

## Results

### Vta3 controls the expression of 1,179 *V*. *dahliae* genes including candidates involved in virulence and regulators of other genetic networks

The transcriptomes of *V*. *dahliae* wild-type and *VTA3* deletion strains were compared to assess the Vta3 genetic network with emphasis on virulence-associated functions. Fungal strains were cultivated in liquid SXM before mycelia were shifted to extracted tomato xylem sap and further incubated for 8 h. Overall, the loss of *VTA3* affected the expression of 1,179 genes. In the presence of *VTA3*, 650 transcripts were induced, while the transcription of 529 genes was reduced. The FungiFun2 web tool [33] was applied for functional enrichment analysis for all significant differentially expressed genes (log_2_(fold change) ≥ 1 or ≤ -1). Thereby, 209 of 1,179 genes were assigned to the five significantly enriched categories (i) heavy metal binding, (ii) heme binding, (iii) secondary metabolism, (iv) disease, virulence and defense and (v) virulence, disease factors (S4 Table). Among the 650 transcripts induced in the presence of *VTA3*, 15 were found in the significantly enriched category ‘disease, virulence and defense’ (S5 Table), including the SnodProt1-like proteins Cp1 and Cp2. Cerato-platanin family proteins, such as SnodProt1, are involved in parasitism, adhesion and development of fungi [53]. Whereas Cp1 contributes to virulence on cotton [54], Cp2 is dispensable for virulence on tomato plants [14]. 95 of the 529 transcripts reduced in the presence of *VTA3* were classified in five significantly enriched categories. Besides secondary metabolism, the deduced proteins are attributed to DNA damage response, repair, recombination, and topology (S6 Table).

Additionally, the deduced proteins of the most strongly induced/reduced transcripts (log_2_(fold change) ≤ -2 or ≥ 2) were manually assigned functions based on BLAST search identification of encoded conserved domains. In the presence of *VTA3*, expression of 100 genes was induced with a log_2_(fold change) ≤ -2 (Fig 1A, S7 Table) and expression of 62 genes was reduced with a log_2_(fold change) ≥ 2 (Fig 1A, S8 Table). The identified candidates have various functions, which are summarized in Fig 1A. For 42 induced and 31 reduced transcripts, no function could be assigned due to the absence of conserved domains or characterized homologs. Among these are two Vta3-induced and four reduced transcripts encoding potential effectors (≤ 200 aa, predicted signal peptide, ≥ 2% cysteines).

Expression of three genes encoding putatively extracellular virulence-associated proteins is induced in the presence of *VTA3*, including an Egh16-like virulence factor domain protein (named Elv1). We further investigated Elv1 functions because Vta3 induces its transcript level 13-fold compared with only four to six-fold for the other two candidates. The increased expression of *ELV1* in the presence of *VTA3* was confirmed by qPCR (Fig 1B). Elv1-related proteins presumably are important during early infection in the rice blast fungus *Magnaporthe oryzae* (formerly *M*. *grisea*) [55].

Expression of two potential transcription factor-encoding genes is reduced in the presence of an intact *VTA3* regulatory gene suggesting a potential direct or indirect repressor function for other genetic networks (Fig 1A). One of the deduced proteins carries only a weak potential basic-leucine zipper motif, whereas the other protein is encoded by the *bona fide MTF1* ortholog of *Aspergillus nidulans mtfA* (S8 Table). MtfA of *Aspergillus* spp. is involved in secondary metabolism and morphogenesis [56,57]. A possible virulence-associated role was suggested in the saprotrophic *Aspergillus flavus* when colonizing peanut seeds [58]. We confirmed by qPCR that Vta3 reduces *MTF1* expression in xylem sap (Fig 1C) as prerequisite for further investigation of possible Mtf1 functions in the pathogenic fungus *V*. *dahliae* during infection of tomato plants.

### The Egh16-like virulence factor Elv1 contributes to *V*. *dahliae* virulence on tomato plants but is dispensable for vegetative growth and conidiation

**Fig 2:**
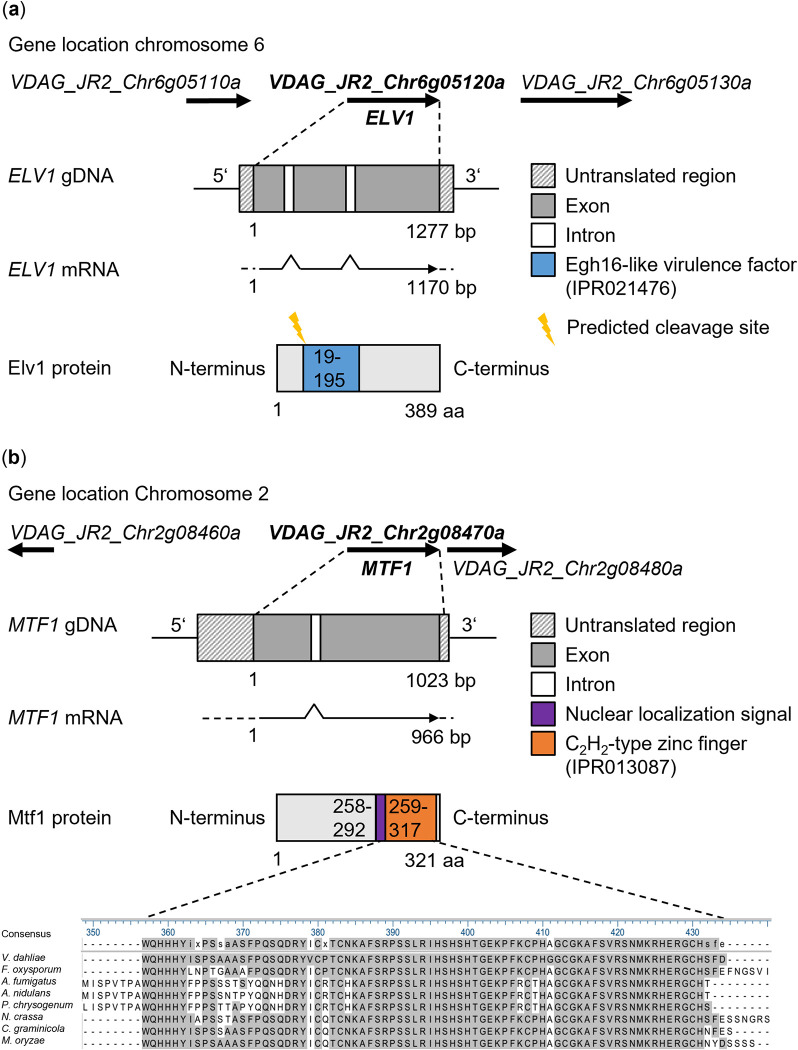
Genomic structure of the Egh16-like virulence factor Elv1 and the Mtf1 transcription factor-encoding genes in *Verticillium dahliae*. Genomic location and neighboring genes with directions of transcription are shown. Intron-exon structures were predicted by Ensembl Fungi. Predicted untranslated regions are represented by color-hatched regions. (a) *ELV1* (*VDAG_JR2_Chr6g05120a*) is located on chromosome six. The 1277 base pair (bp) *ELV1* open reading frame includes three exons (in gray) and two introns (in white). The Elv1 protein consists of 389 amino acids (aa) and contains the Egh16-like virulence factor domain (IPR021476; 19–195 aa; in blue) predicted by InterPro. A signal peptide was predicted with a cleavage site between amino acid residues 19 and 20 (probability 0.97; in yellow) using SignalP 6.0. (b) *MTF1* (*VDAG_JR2_Chr2g08470a*) is located on chromosome two. The 1023 bp *V*. *dahliae MTF1* open reading frame harbors two exons (in gray) and one intron (in white). The Mtf1 protein consists of 321 aa. It contains a putative nuclear localization signal (258–292 aa; in purple) predicted by cNLS Mapper and a highly conserved C_2_H_2_-type zinc finger domain (IPR013087; 259–317 aa; in orange) predicted by InterPro. An amino acid sequence alignment of the conserved region in different ascomycetes is shown. Similar amino acids are highlighted in gray.

Consistent with the RNA sequencing data, promoter analysis using the FIMO web tool [50] revealed that *ELV1* has consensus sequences in its 5’ regulatory region for possible binding of Crt1/RFX1 (DNA-binding motif discovered by [59]), an ortholog of Vta3 (S4A Fig). The *V*. *dahliae ELV1* ORF consists of 1,277 bp, including three exons and two introns, encoding a 389 amino acid (aa) protein with a predicted molecular weight of 38.9 kDa (Fig 2A). An N-terminal signal peptide that directs the protein for secretion was predicted by SignalP 6.0 [51]. The protein harbors an Egh16-like virulence factor domain (IPR021476; 19–195 aa). Three further proteins with this domain are annotated in JR2 and share 60–68% identity with Elv1. A BLAST search revealed that Elv1 is homologous to Egh16 originally identified from a cDNA library of germinating conidia of the powdery mildew fungus *Blumeria* (formerly *Erysiphe*) *graminis* f. sp *hordei* [60], and to its homolog Egh16h1. Other related proteins with approximately 60% similarity are the appressoria-specific virulence factors Gas1 and Gas2 of *M*. *oryzae*, which are involved in lesion development in rice and barley [55]. Functionally uncharacterized putative homologs with similarities between 21–66% were found in the phytopathogens *Colletotrichum graminicola* and *F*. *oxysporum*, as well as in *Neurospora crassa* and *A*. *fumigatus* (S5A Fig). Predicted homologs exist only in filamentous fungi, but not in yeasts such as *S*. *cerevisiae* or *Candida albicans*.

**Fig 3:**
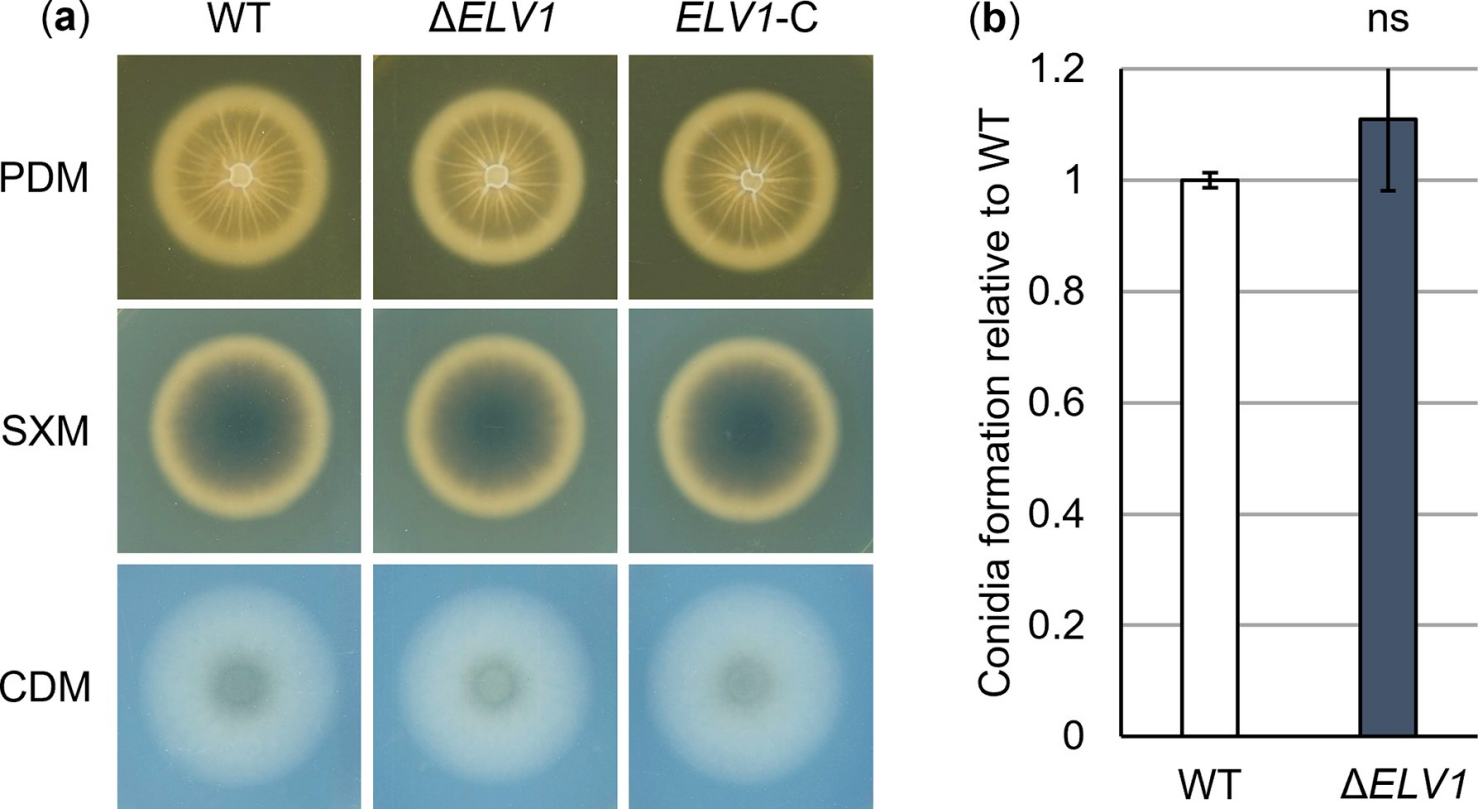
The Egh16 homolog-encoding gene *ELV1* of *Verticillium dahliae* is dispensable for vegetative growth and conidiospore formation. The *V*. *dahliae ELV1* deletion strain (Δ*ELV1*) was compared with the JR2 wild-type (WT) in terms of morphology on plate and conidia formation. (a) 50,000 spores of respective strains were point-inoculated onto plates containing simulated xylem medium (SXM), potato dextrose medium (PDM) or Czapek-Dox medium (CDM) and incubated at 25°C for 10 days. Bottom view scans depict a similar phenotype of Δ*ELV1* compared with the wild-type and complementation strain (*ELV1*-C) on all media tested. (b) Conidia formation was quantified in five-day-old cultures in liquid SXM incubated at 25°C with constant agitation after inoculation of 4,000 spores ml^-1^. Error bars represent the SD of the means of two independent experiments, each with two biological and four technical replicates (*n* = 4). No difference in the ability to form conidiospores was detected between Δ*ELV1* and the wild-type (ns, not significant; calculated using *t*-test).

We replaced the *ELV1* ORF by a nourseothricin resistance marker. In-locus complemented strains with *ELV1* alongside a hygromycin resistance marker were generated. Strains were verified by Southern hybridization (S2 Fig). A possible function of Elv1 in fungal growth outside the plant was tested by point-inoculating spores of wild-type, *ELV1* deletion and complementation strains on solid media. Conidiation was analyzed in liquid SXM. We did not observe any defects of the *ELV1* deletion strain in vegetative growth, microsclerotia or conidia formation (Fig 3A and 3B).

**Fig 4:**
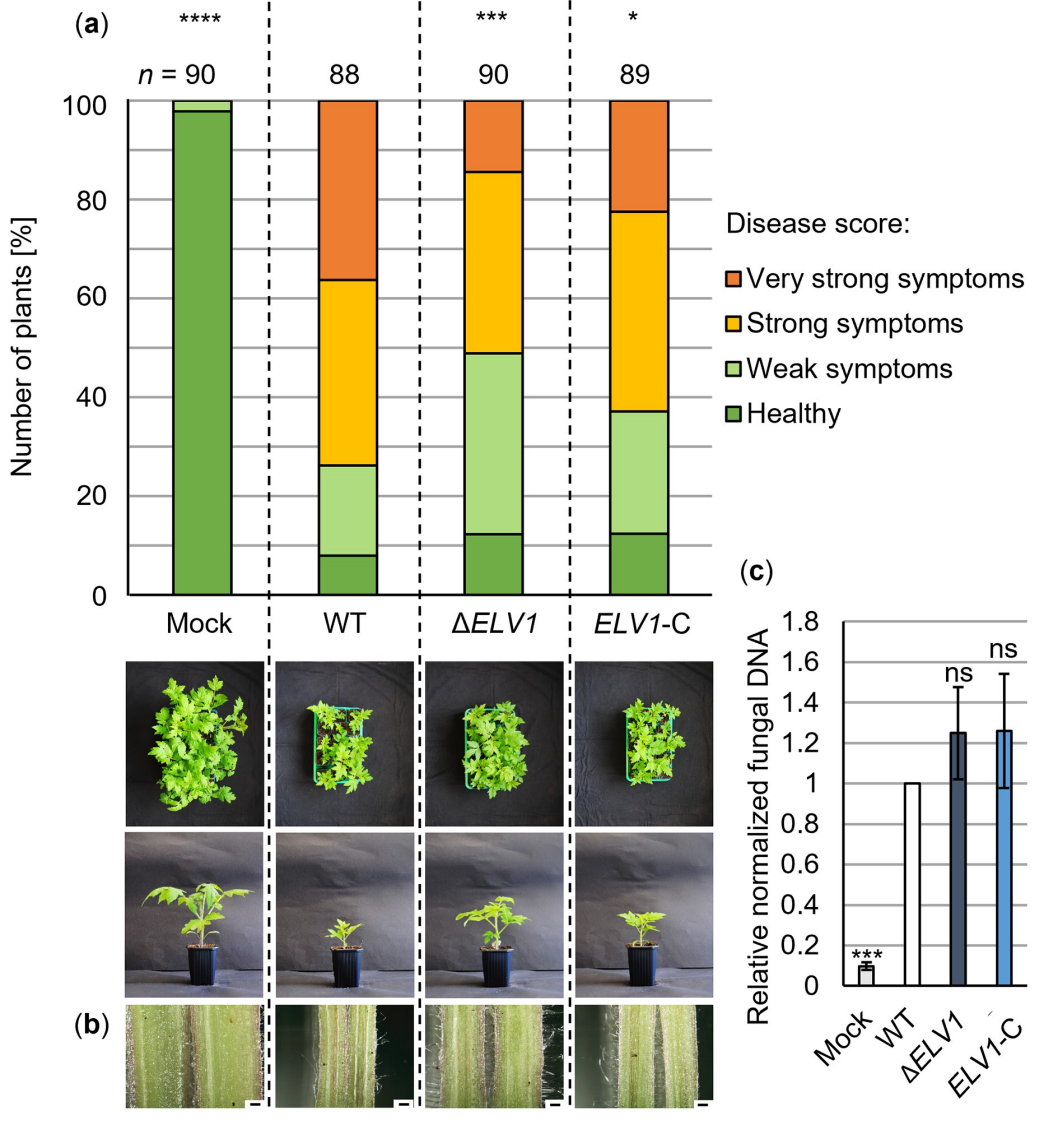
*Verticillium dahliae* Elv1 contributes to virulence on tomato plants. The *in-planta* phenotype of the *V*. *dahliae ELV1* deletion strain (Δ*ELV1*) was compared with the JR2 wild-type (WT) and the complementation strain (*ELV1*-C). Tomato seedlings (10-day-old) were treated with spores or water (mock) by root-dipping. The plants were incubated in a climate chamber under 16 h: 8 h light: dark at 22–25°C. The experiment was performed twice. (a) The disease score was determined at 21 dpi and includes plant height, longest leaf length and plant weight. The relative number of plants with certain disease scores is summarized in the stack diagram (*n* = number of treated plants). Significance was calculated using two-tailed Mann-Whitney *U* tests (*, *P* < 0.05; ***, *P* < 0.001; ****, *P* < 0.0001). Treatment of tomato seedlings with Δ*ELV1* spores resulted in approximately 25% more plants exhibiting no or only weak symptoms compared with wild-type-infected plants. An overview of plants and a single representative plant per treatment are shown. (b) Cross-sections of representative hypocotyls (scale = 500 μm) depict discoloration in plants treated with wild-type, *ELV1* deletion or complementation strains, but not in mock-inoculated plants. (c) Fungal DNA was analyzed by quantitative PCR relative to plant DNA purified from 14 to 15 hypocotyls per treatment (*n* = 1). Shown are the means of six biological replicates ± SE of the mean. Significance was calculated using *t*-tests (ns, not significant; ***, *P* < 0.001). Significantly less fungal DNA was detected in mock than in fungus-inoculated plants.

*ELV1* expression was strongly induced in the presence of *VTA3* in xylem sap (Fig 1A and 1B). Therefore, we investigated whether *V*. *dahliae* Elv1 is involved in tomato plant colonization. Disease symptoms were assessed at 21 dpi of 10-day-old tomato seedlings treated with spores of wild-type, *ELV1* deletion or complementation strains or water (mock). A general disease index was calculated from the measured plant height, weight and longest leaf length [14,30]. The diagram in Fig 4A displays the number of plants classified as healthy or with weak, strong or very strong symptoms compared with mock-inoculated plants. Deletion of *ELV1* resulted in approximately 25% more plants with no or only weak symptoms compared with wild-type-infected plants. Hypocotyl discoloration as a sign of fungal infection was observed in cross-sections of plants treated with wild-type, *ELV1* deletion or complementation strains, but not in mock-inoculated plants (Fig 4B). Fungal DNA (Fig 4C) and PR gene expression (S6 Fig) were quantified to address possible alterations in plant colonization or immune responses, respectively. Both were similar to the wild-type in plants infected by *ELV1* deletion and complementation strains. Taken together, these data suggest that Elv1 is dispensable for growth, conidiation and initial plant colonization but contributes to late and full disease development in tomato plants.

### The C_2_H_2_-type zinc-finger transcriptional regulator Mtf1 is present during vegetative growth of *V*. *dahliae*

Vta3-dependent control of the virulence factor Elv1-encoding gene supports a temporal Vta3 function in late *V*. *dahliae* plant xylem colonization. Therefore, we examined whether the reduced expression of the Vta3-dependent transcriptional regulator Mtf1 is also associated with late stages of fungus-induced tomato plant disease and the subsequent formation of survival structures. Like *ELV1*, *MTF1* also carries consensus sequences for putative binding of Vta3 as the Crt1/RFX1 counterpart of *V*. *dahliae* in its 5’ regulatory region (S4B Fig). The *A*. *nidulans* counterpart MtfA was first identified in a mutant strain that had regained the ability to produce sterigmatocystin protecting the overwintering fruiting bodies of this fungus, which is lost in *veA* deletion strains [56]. Both VeA and MtfA are master transcriptional regulators of secondary metabolism and development in *A*. *nidulans* and *A*. *fumigatus*, although different underlying genetic pathways are involved [61]. In *V*. *dahliae*, the ortholog *MTF1* is comprised of a 1,023 bp ORF including two exons and one intron. The deduced protein consists of 321 aa and has a molecular weight of approximately 34.9 kDa (Fig 2B). The Mtf1 sequence harbors a putative nuclear localization signal (258–292 aa) and a C_2_H_2_-type zinc finger domain (IPR013087) with high conservation among ascomycetes. A comparison of the *V*. *dahliae* Mtf1 sequence with putative orthologs in other fungi, including the phytopathogens *C*. *graminicola* and *M*. *oryzae*, and the model organism *N*. *crassa*, revealed similarities of 49–69% (S5B Fig). An ortholog was not found in either *S*. *cerevisiae* or humans.

We examined the production of a functional Mtf1 protein during growth and development of *V*. *dahliae* using a strain expressing *MTF1* fused to *GFP* and compared it with the production of Vta3 fused to GFP (*VTA3-GFP* strain from [17]). Phenotypic examination of the *GFP-MTF1*-expressing strain revealed that it resembles the wild-type, suggesting that the fusion protein is functional (S7 Fig). Vta3-GFP (115 kDa) was detected under condition favoring vegetative growth and conidia production as well as in minimal medium and natural plant xylem sap. On SXM plates, where increasing numbers of microsclerotia are formed over time, Vta3-GFP was visible after two days, whereas at later time points only free GFP (27 kDa) was detectable. In contrast, the GFP-Mtf1 fusion protein with a size of about 62 kDa was detectable only when *V*. *dahliae* grew predominantly vegetatively, and a weak signal corresponding to GFP-Mtf1 was visible in minimal medium (S7 Fig). Consistent with the Vta3-dependent downregulation of *MTF1* transcription (Fig 1A and 1C), strong signals corresponding to Vta3-GFP were detected when *V*. *dahliae* grew in xylem sap, whereas GFP-Mtf1 was not found (S7 Fig). These results indicate that Vta3 is produced in the plant xylem and underline a possible function of Vta3 in reducing Mtf1 levels in the plant.

### Mtf1 enhances microsclerotia formation in *V*. *dahliae*

Deletion strains were constructed carrying a hygromycin resistance marker in place of the *MTF1* ORF to investigate Mtf1 functions. In-locus complemented strains were generated by inserting *MTF1* into the deletion strain under its native promoter with a nourseothricin resistance marker. All strains were verified by Southern hybridization (S3 Fig).

**Fig 5:**
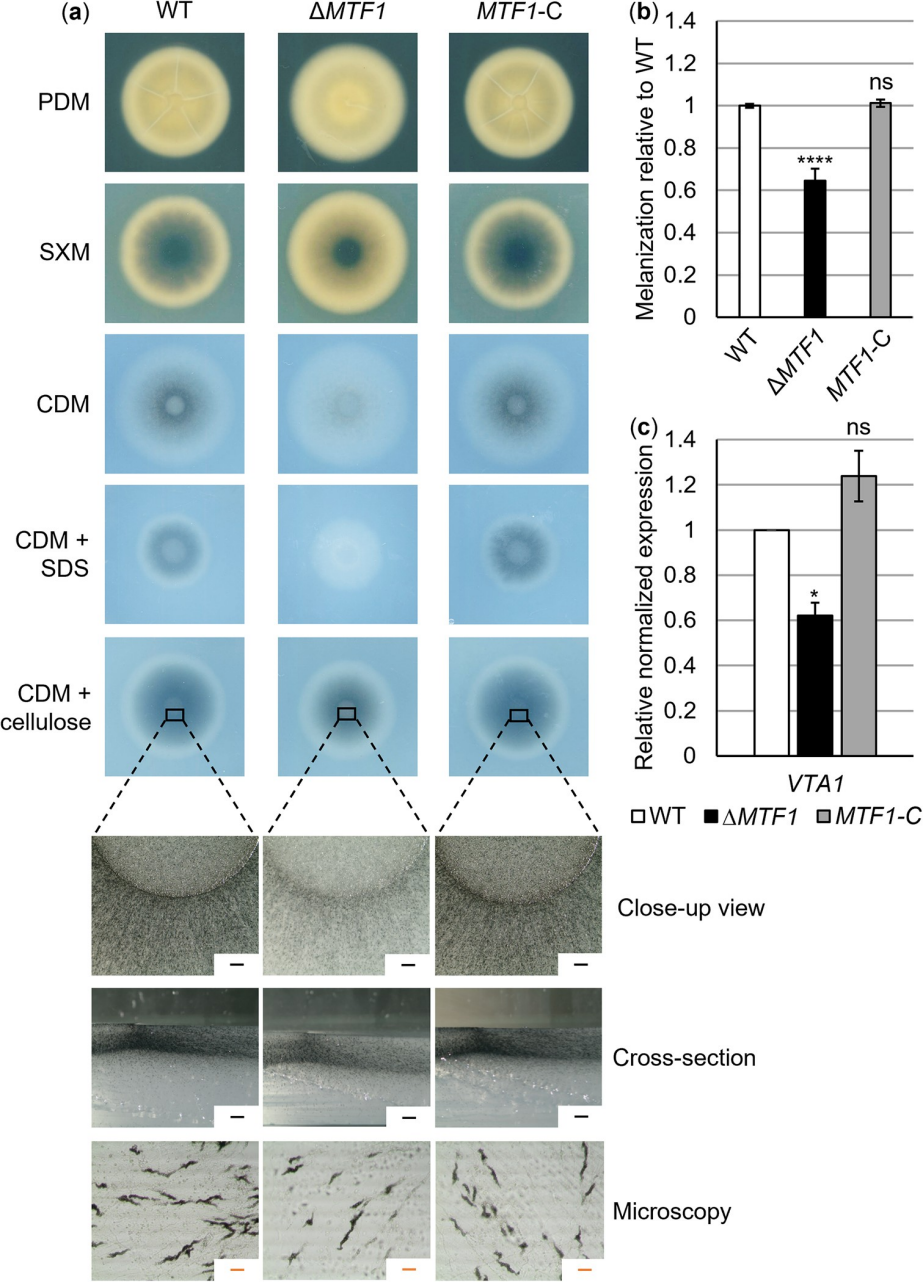
The transcriptional regulator-encoding gene *MTF1* of *Verticillium dahliae* is required for formation of microsclerotia at levels comparable to the wild-type. (a, b) The *ex-planta* phenotype of the *V*. *dahliae MTF1* deletion strain (Δ*MTF1*) was compared with the JR2 wild-type (WT) and the *MTF1* complementation strain (*MTF1*-C). 50,000 spores of respective strains were point-inoculated onto plates containing simulated xylem medium (SXM), potato dextrose medium (PDM) or Czapek-Dox medium (CDM) and incubated at 25°C for 10 days. (a) Bottom view scans depict fewer microsclerotia formed by Δ*MTF1* compared with the wild-type and *MTF1*-C on all tested media. For CDM + cellulose plates, close-up views, cross-sections of colony centers and microscopic images of fungal material from the colony centers are shown (black scale in upper two rows = 500 μm, orange scale in lower row = 50 μm). (b) Melanization of fungal colonies grown on CDM + cellulose was quantified as a measure of the number of microsclerotia formed. Pictures were taken from top view scans of the colonies after removal of aerial mycelium. The brightness factor of three colonies per transformant (*n* = 1) was calculated using the ROI Manager of ImageJ software. Three independent experiments were performed. Means ± SD are shown. Significant differences from wild-type were determined using *t*-tests. Melanization of Δ*MTF1* was reduced to approximately 60% of wild-type levels (****, *P* < 0.0001), which is rescued in *MTF1*-C (ns, not significant). (c) Expression levels of *VTA1*, which encodes the microsclerotia melanization-controlling transcription factor Vta1, were compared between wild-type, *MTF1* deletion and complementation strains. RNA was extracted from mycelia grown in liquid SXM for five days and subsequently in xylem sap for 8 h. Means of three biological replicates normalized to transcript levels of wild-type and references *H2A* and *EIF2B* with error bars representing the SE of the means are shown. *VTA1* expression was reduced to 60% in Δ*MTF1* (*, *P* < 0.05) and wild-type-like in *MTF1*-C (calculated using *t*-tests).

Microsclerotia are specialized melanized cells that enable *V*. *dahliae* to survive in the soil for several years [1,62]. Although GFP-fused Mtf1 was not detected under conditions favoring microsclerotia production (S7 Fig), the transcriptional regulator may still be involved in the induction of microsclerotia formation. The ability to form microsclerotia was compared between wild-type, *MTF1* deletion and complementation strains by point-inoculation of spores on different media. Melanization of fungal colonies decreased in the absence of *MTF1* (Fig 5A). Microscopic observations of fungal material from colony centers implied that *MTF1* is required for microsclerotia abundance but not for their size or shape. *MTF1* deletion did not result in any growth defect or other phenotypic differences compared with wild-type.

The melanization of fungal colonies grown on cellulose-containing CDM plates was quantified as a measure for the number of microsclerotia formed. There was a 40% reduction in melanization in the *MTF1* deletion strain compared with wild-type (Fig 5B). The complementation strain was able to form microsclerotia at levels comparable to the wild-type. The transcription factors Vta1 and Cmr1 are produced during microsclerotia development and are required for melanization [30,63]. *VTA1* expression was reduced by 40% in the *MTF1* deletion strain compared with wild-type (Fig 5C), while *CMR1* expression was not affected by *MTF1* deletion (S8 Fig). These results suggest that Mtf1 promotes microsclerotia formation and *VTA1* expression to favor melanization independent of Cmr1, which may act in a parallel signaling pathway.

### Colonization of *Arabidopsis* roots by *V*. *dahliae* is independent of *MTF1*

**Fig 6:**
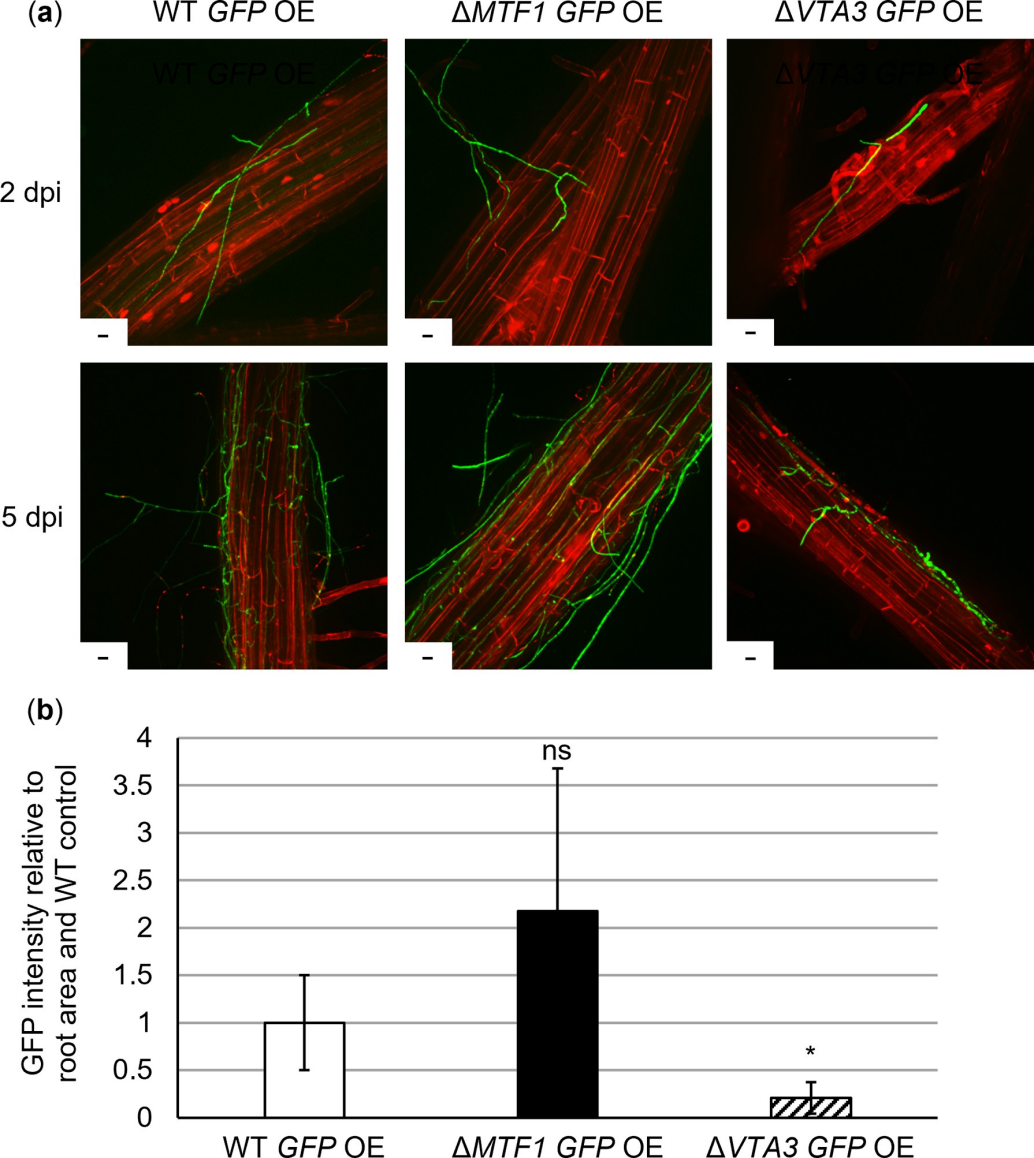
*MTF1* is dispensable for *Arabidopsis thaliana* root colonization by *Verticillium dahliae*. *A*. *thaliana* seedlings (three-week-old) were treated by root-dipping with spores of *V*. *dahliae* JR2 wild-type (WT *GFP* OE), Δ*MTF1* (Δ*MTF1 GFP* OE) or Δ*VTA3* (Δ*VTA3 GFP* OE) strains constitutively expressing (OE) ectopically integrated *GFP*. Fungal colonization of the roots was observed at two and five days post inoculation (dpi). Root cells were stained with 0.05% propidium iodide/0.01% silwet solution. Two independent experiments were performed. (a) Representative fluorescence micrographs depict similar initial (2 dpi) and complete root colonization (5 dpi) by *V*. *dahliae* wild-type or *MTF1* deletion strains compared with the Δ*VTA3* control, which did not propagate to the same extent on the root surface (scale = 10 μm). (b) Quantification of fungal root colonization was performed at 5 dpi. For each root, 10 to 15 images were taken at randomly selected sections. Roots of two plants per treatment were considered as one biological replicate. The green fluorescence of *GFP*-expressing fungal hyphae was quantified relative to the root area. Shown are the means of four biological replicates ± SD. A *t*-test was used for statistical analysis. No significant difference was detected between plant roots colonized by the wild-type or the *MTF1* deletion strain (ns, not significant), in contrast to the Δ*VTA3* control strain, which is impaired in its ability to colonize plant roots (*, *P* < 0.05).

Possible Mtf1 functions in different stages of plant infection were addressed. Roots are the entry points to the host for *V*. *dahliae* as a soil-borne pathogen [1]. *Arabidopsis thaliana* roots were treated with spores of strains expressing ectopically integrated *GFP* under control of a *gpdA* promoter. Root colonization of wild-type and *MTF1* and *VTA3* deletion strains was compared. Transcription factor-encoding *VTA3* is required for fungal propagation on *A*. *thaliana* roots [17]. By fluorescence microscopy, we show that roots were similarly colonized by wild-type and *MTF1* deletion strains (Fig 6A). Initial colonization of the root surface was observed by 2 dpi, and whole roots were covered with hyphae of wild-type or *MTF1* deletion strains by 5 dpi. In contrast, the *VTA3* deletion strain was significantly impaired in propagation on *A*. *thaliana* roots (*P* < 0.05; Fig 6B). These results indicate that *MTF1* is not necessary for attachment and initial growth on root surface and that the regulation of root colonization by Vta3 is an Mtf1-independent process.

### Mtf1 increases the virulence of *V*. *dahliae* on tomato plants

**Fig 7:**
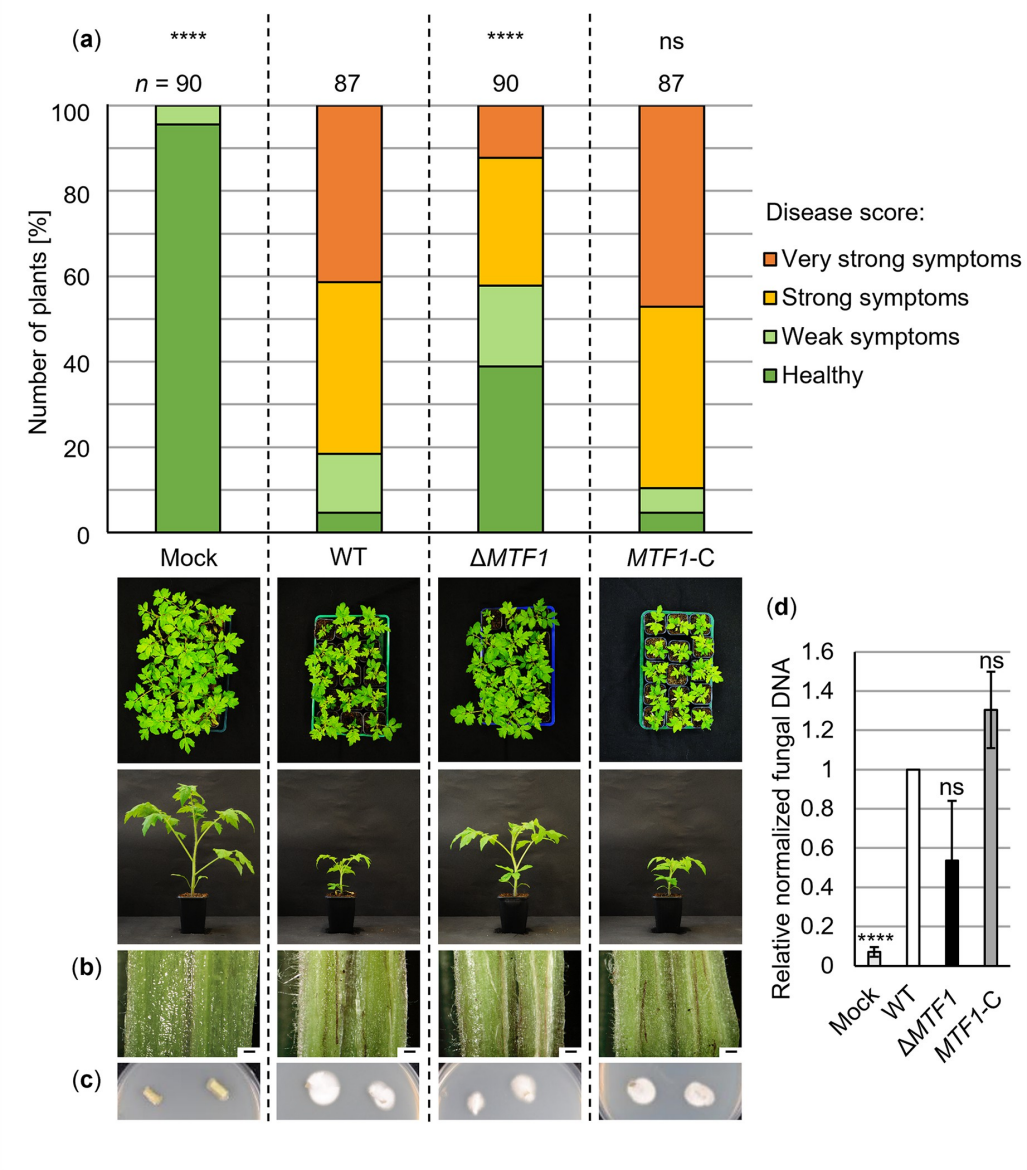
*Verticillium dahliae* Mtf1 increases disease symptom severity in tomato plants. Tomato seedlings (10-day-old) were root-inoculated with spores of the JR2 wild-type (WT), *MTF1* deletion (Δ*MTF1*) and complementation strains (*MTF1*-C), respectively. Water-treated plants (mock) served as controls. The plants were incubated in a climate chamber under 16 h: 8 h light: dark at 22–25°C. (a) The disease score was determined at 21 dpi and includes plant height, longest leaf length and plant weight. The relative number of plants with certain disease scores from three independent experiments is summarized in the stack diagram (*n* = number of treated plants). Plants treated with Δ*MTF1* were healthier and less individuals had very strong disease symptoms than plants infected by the wild-type or *MTF1*-C (****, *P* < 0.0001; ns, not significant; calculated using two-tailed Mann-Whitney *U* tests). An overview of plants and a single representative plant per treatment are shown. (b) Cross-sections of representative hypocotyls (scale = 500 μm) depict discoloration in plants treated with wild-type, *MTF1* deletion or complementation strains, but not in mock-inoculated plants. (c) Surface-sterilized stem sections of plants treated with all tested strains, but not of mock-inoculated plants, displayed fungal outgrowth after incubation on potato dextrose medium with chloramphenicol at 25°C for 7 d. (d) Fungal DNA was analyzed by quantitative PCR relative to plant DNA purified from hypocotyls. Hypocotyls of 13 to 15 plants per treatment were pooled (*n* = 1). Means of five to six biological replicates ± SE of the mean are shown. Significance was calculated using *t*-tests. Significantly less fungal DNA was detected in mock than in fungus-inoculated plants.

The functions of Mtf1 in later stages of plant colonization and symptom induction were examined by pathogenicity assays with the host plant tomato. Roots of 10-day-old tomato seedlings were treated with spores of wild-type, *MTF1* deletion or complementation strains and disease symptoms were assessed at 21 dpi. The general disease scoring revealed that more than 80% of plants displayed strong and very strong symptoms when infected by either wild-type or *MTF1* complementation strains, while less than 20% remained healthy or showed only weak symptoms. In contrast, this disease rate was halved to approximately 40% of plants displaying strong and very strong symptoms when infected by the *MTF1* deletion strain. Nearly 60% of plants treated with the *MTF1* deletion strain were either healthy or displayed only a mild stunting phenotype (Fig 7A). This suggests a strong contribution of Mtf1 to *V*. *dahliae* virulence.

Fungal infection leads to discoloration of the plant hypocotyl visible in cross-sections (Fig 7B). Brownish streaks were observed in 95% of wild-type-infected plants, in 91% of *MTF1* complementation strain-infected plants and in 85% of *MTF1* deletion strain-treated plants. No discoloration was evident in hypocotyls of mock-inoculated plants. The presence of *V*. *dahliae* inside the plant was analyzed by re-isolating mycelium from stem sections on PDM plates and by quantifying fungal DNA in hypocotyls. No fungus grew from stems of mock-inoculated plants. Fungal material was successfully re-isolated from 75% of wild-type-infected stem sections and from 74% of plants infected by the *MTF1* complementation strain. In plants inoculated with the *MTF1* deletion strain, the fungus was re-isolated from 62% of stem sections (Fig 7C). Fungal DNA in hypocotyls was analyzed by qPCR relative to plant DNA. Although less fungal DNA was measured in *MTF1* deletion strain-inoculated plants compared with wild-type-inoculated plants, the reduction was not significant (Fig 7D).

Coupled with the observations that the *MTF1* deletion strain is not impaired in hyphal growth (Figs 5 and 6) or conidiospore production (S9 Fig), our data indicate that Mtf1 is involved in virulence and increases symptom severity in tomato plants by acting at later stages of plant colonization.

### Mtf1 acts in the sequential Vta3-Vta2 network required for plant disease

**Fig 8:**
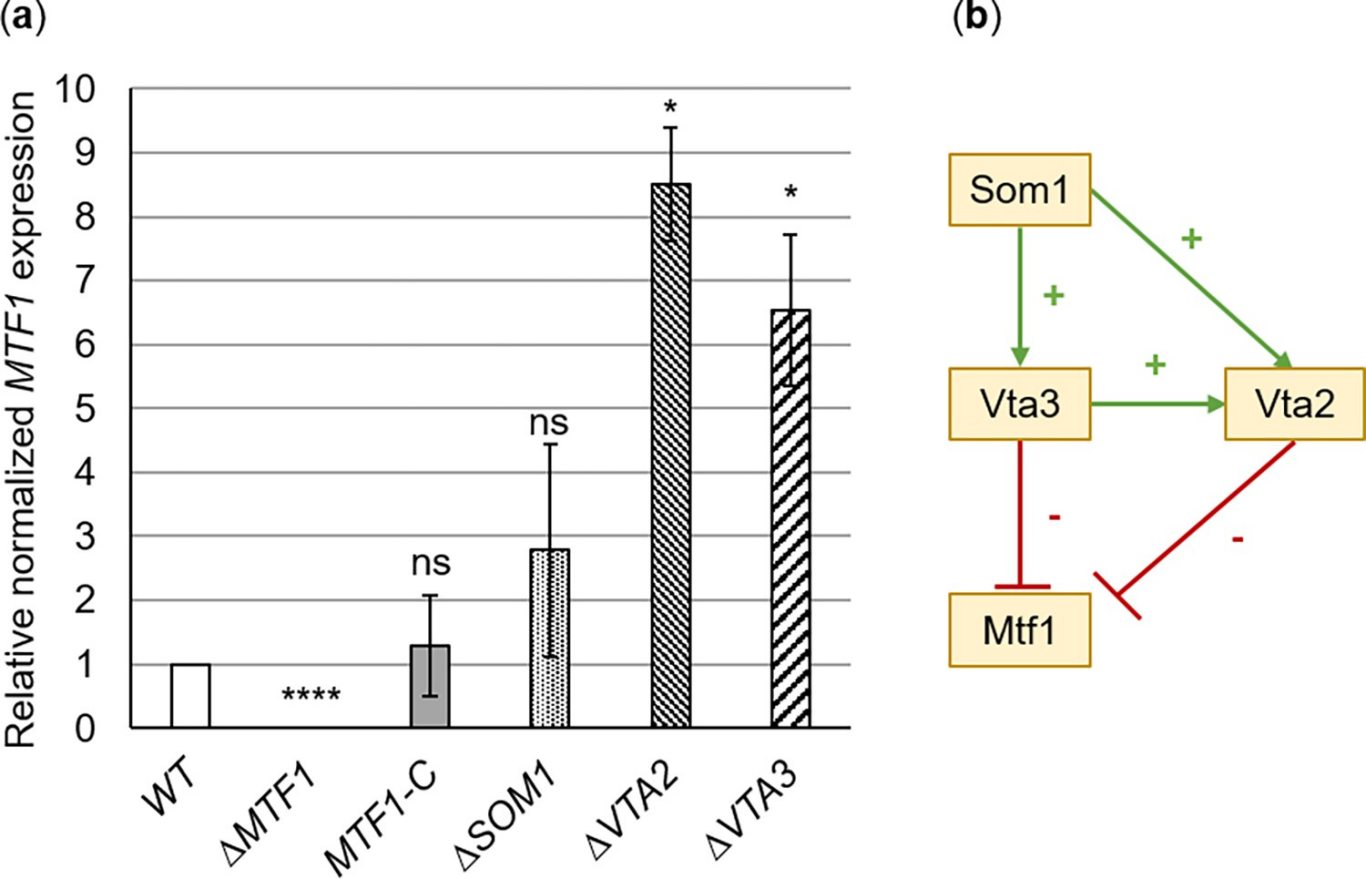
*MTF1* expression is affected by other pathogenesis-related transcription factors in *Verticillium dahliae*. (a) *MTF1* transcript levels were analyzed by reverse transcription-quantitative PCR in JR2 wild-type (WT), *MTF1* complementation (*MTF1*-C), *MTF1* deletion (Δ*MTF1*) and other gene deletion strains, which were cultured in extracted tomato xylem sap for 8 h following preculture in simulated xylem medium. *MTF1* expression was normalized to the transcript levels of reference genes *H2A* and *EIF2B* and gene expression levels in wild-type were set to one. Significant differences from wild-type were determined using *t*-tests. Means of three independent experiments are shown with error bars representing the SE of the means. *MTF1* expression was not measured in Δ*MTF1* (****, *P* < 0.0001) but was wild-type-like in *MTF1*-C (ns, not significant). *MTF1* transcript levels were not significantly increased in the *SOM1* deletion strain (Δ*SOM1*). In the *VTA2* deletion strain (Δ*VTA2*), expression of *MTF1* was induced eight-fold, and six-fold in the *VTA3* deletion strain (Δ*VTA3*) (*, *P* < 0.05). (b) The schematic displays the control network of pathogenesis-related transcription factors in *V*. *dahliae*. Connections indicate direct or indirect control. Som1 induces expression of *VTA3* and *VTA2*, whereas Vta3 induces *VTA2* expression [17]. *MTF1* expression is reduced in the presence of *VTA3* (Figs 1A, 1C and 8A) and *VTA2* (Fig 8A), respectively.

Transcriptional profiling of *V*. *dahliae* strains cultivated in xylem sap revealed that the transcriptional regulator Vta3 reduces *MTF1* expression either as a repressor or indirectly through a yet elusive molecular mechanism (Fig 1A and 1C). A possible interplay of *MTF1* expression with other prominent transcription factors involved in pathogenicity (Som1 and Vta2) was examined in corresponding deletion strains using qPCR and brought into context with the Vta3-dependently reduced expression of *MTF1* (Fig 8A). As shown previously in Fig 1C, *MTF1* expression was reduced six-fold in the presence of *VTA3*. Som1 and Vta3 control genetic networks for sequential steps of plant root penetration and infection prior to Vta2-controlled genes [16,17]. *MTF1* expression was induced eight-fold in the *VTA2* deletion strain in comparison with the wild-type, whereas no *MTF1* expression was measured in the *MTF1* deletion strain. *MTF1* transcript levels were not significantly elevated in the *SOM1* deletion strain. Notably, the transcription of *SOM1*, *VTA3* and *VTA2* does not change significantly when an intact *MTF1* gene is present or not (S10B and S10C Fig).

Thus, *MTF1* expression is affected by the concerted and presumably sequential action of Vta3 and Vta2, a second regulatory protein controlling adhesion and plant root colonization. This reflects a complex network in which Som1 induces *VTA3* and *VTA2* expression. Vta3 in turn induces *VTA2* expression [17] (Fig 8B). Mtf1 is therefore integrated into a regulatory network required for fungal development and virulence.

### Mtf1 induces expression of fungal effectors and tomato defense signaling

**Fig 9:**
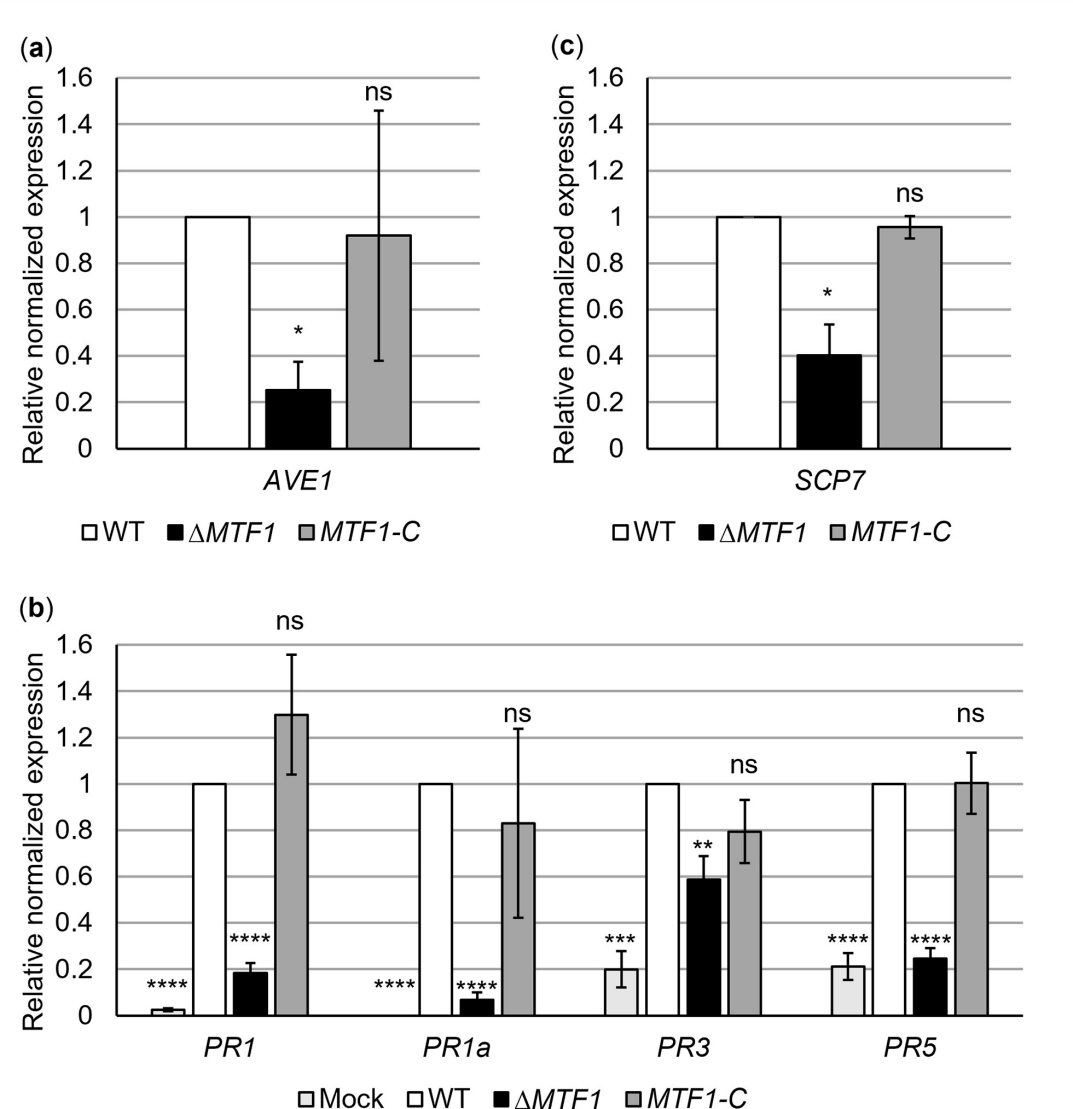
Expression of fungal effector genes and pathogenesis-related protein (PR) genes in tomato plants is increased in the presence of *Verticillium dahliae MTF1*. Transcript levels were analyzed by reverse transcription-quantitative PCR and gene expression levels in wild-type were set to one. Significant differences from wild-type (WT) were determined using *t*-tests. Means of three independent experiments are shown with error bars representing the SE of the means. (a,c) Strains were cultured in extracted tomato xylem sap for 8 h following preculture in simulated xylem medium. Means of three biological replicates normalized to the transcript levels of reference genes *H2A* and *EIF2B* are shown. (a) In the absence of *MTF1* (Δ*MTF1*), expression of the virulence factor Ave1-encoding gene was reduced by almost 80% compared with wild-type and the *MTF1* complementation strain (*MTF1*-C) (*, *P* < 0.05; ns, not significant). (b) Expression levels of tomato PR genes were quantified in hypocotyls 21 days after inoculation with *V*. *dahliae* JR2 wild-type, Δ*MTF1* or *MTF1-*C spores. Water-treated plants (mock) served as controls. Hypocotyls from 13 to 15 plants per treatment were pooled (*n* = 1). Means of six biological replicates normalized to the transcript levels of reference genes *EF1α* and *αTUB* are shown. The expression levels of *PR1*, *PR1a*, *PR3* and *PR5* did not differ significantly between wild-type and *MTF1-*C-infected plants. In plants treated with water or Δ*MTF1*, the expression levels of PR genes were significantly lower (**, *P* < 0.01; ***, *P* < 0.001; ****, *P* < 0.0001). (c) The transcript level of the Scp7 effector-encoding gene was significantly reduced in Δ*MTF1* to 40% of the wild-type level.

Fungal pathogens secrete effector proteins for successful colonization and manipulation of plant defense responses. The Ave1 effector is recognized by the cell-surface receptor-like protein Ve1 but acts as a virulence factor on tomato plants lacking Ve1 [64]. *AVE1* transcript levels were compared between wild-type, *MTF1* deletion and complementation strains cultured in xylem sap. *AVE1* expression was reduced to 25% in the absence of *MTF1* (Fig 9A). This Mtf1-mediated activation of Ave1 might explain the reduced aggressiveness of the *MTF1* deletion strain on tomato cultivars that lack *Ve1* (Fig 7).

PR gene expression was examined in tomato hypocotyls by qPCR at 21 dpi to investigate variations in host responses to colonization by *V*. *dahliae*. Mock-inoculated plants served as controls. SA-inducible *PR1a* expression was previously used as a marker of defense signals in tomato plants [65,66]. Expression of three other putative PR genes (*PR1*, *PR3* and *PR5*) is known to be elevated in symptomatic lettuce leaves after *V*. *dahliae* infection [67]. Transcript levels of all tested PR genes were strongly induced in plants infected by the wild type strain of *V*. *dahliae*, suggesting that fungal infection activates SA signaling in tomato. PR gene expression in plants infected by the *MTF1* complementation strain was comparable to wild-type. In plants inoculated with the *MTF1* deletion strain, *PR1*, *PR1a* and *PR5* expression was similar to mock-inoculated plants and significantly reduced (*P* < 0.0001) compared with wild-type-infected plants. *PR3* expression was decreased to 60% of the wild-type level in plants treated with the *MTF1* deletion strain (Fig 9B).

The small, secreted, cysteine-containing protein Scp7 from *V*. *dahliae* induces expression of the SA-marker genes *PR1* and *PR2* in *Nicotiana benthamiana*, suggesting activation of the SA signaling pathway [68]. We examined *SCP7* transcript levels during xylem sap cultivation in the *MTF1* deletion strain, which cannot fully induce PR gene expression in tomato plants. In the absence of *MTF1*, *SCP7* transcript levels were reduced to 40% of wild-type (Fig 9C). *SCP7* expression was similar to the wild-type in the *MTF1* complementation strain. These results suggest that Mtf1 induces gene expression of the Scp7 effector, leading to the induction of PR gene expression in tomato plants.

**Fig 10:**
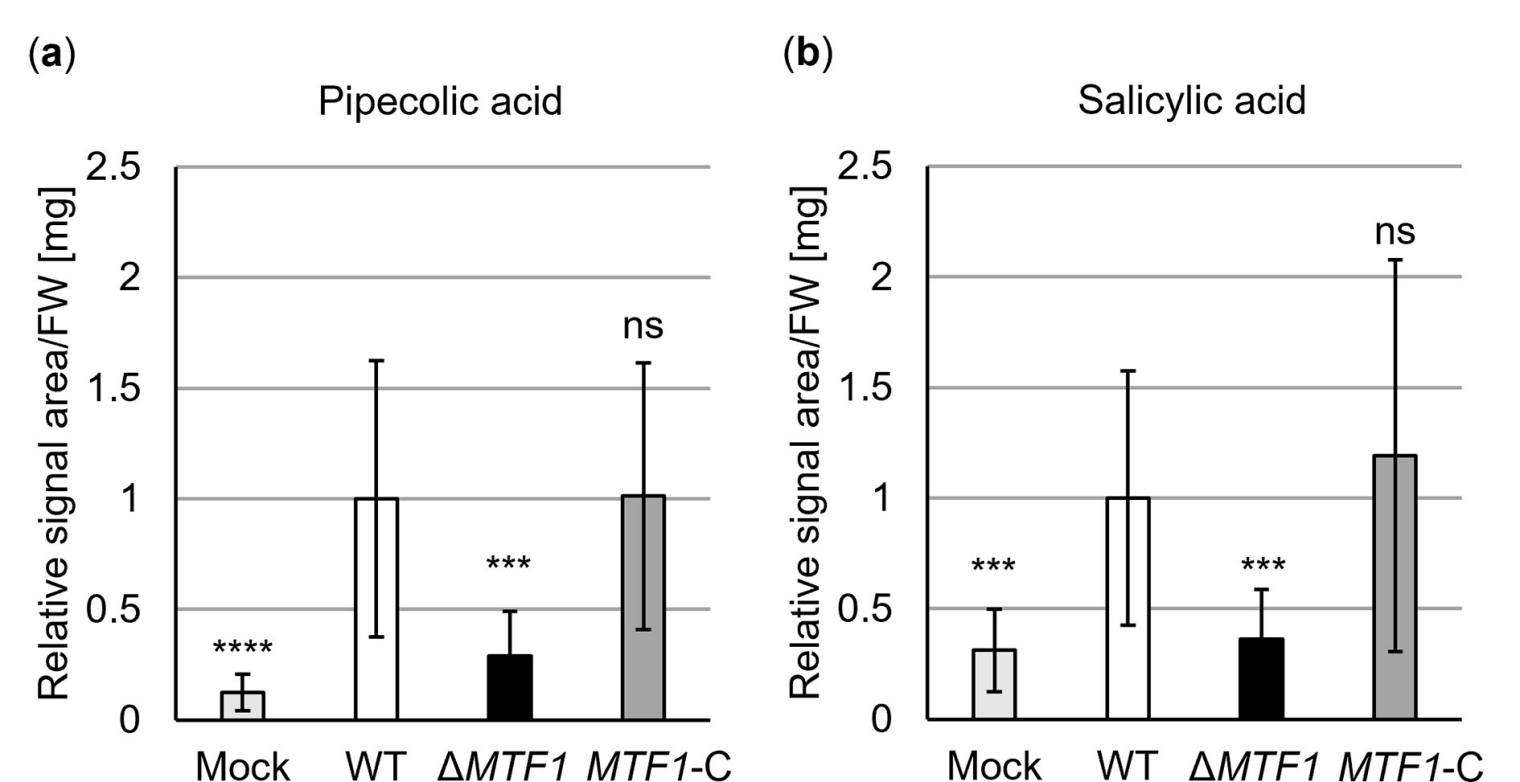
The levels of (a) pipecolic acid and (b) salicylic acid in tomato leaves are elevated after infection with wild-type *Verticillium dahliae* but not after inoculation with *MTF1* deletion strains. Phytohormones were analyzed in leaves 21 days after root treatment with water (mock) or spores of indicated strains. The relative signal area per fresh weight (FW) was calculated and normalized to wild-type (set to one). Means of 15 plants per treatment from two independent experiments are shown with error bars representing SD. Significant differences from wild-type (WT) were determined using *t*-tests. Plant infection by wild-type or *MTF1* complementation (*MTF1*-C) strains led to the induction of pipecolic acid and salicylic acid levels compared with mock, whereas treatment with spores of the *MTF1* deletion strain (Δ*MTF1*) resulted in mock-like phytohormone levels that were significantly lower compared with wild-type (ns, not significant; **, *P* < 0.01; ***, *P* < 0.001; ****, *P* < 0.0001).

An additional phytohormone analysis was performed on tomato leaves after root treatment with water or *V*. *dahliae* spores to emphasize the analysis of PR gene expression. Wild-type, *MTF1* deletion and complementation strains were used and the relative signal area per fresh weight was calculated. We analyzed levels of Pip, which induces resistance of tomato plants to *Botrytis cinerea* and *Pseudomonas syringae* pv. *tomato* [69] and levels of SA, which functions in tomato defense against various pathogens [70]. Plant infection by *V*. *dahliae* wild-type or *MTF1* complementation strains resulted in elevated Pip and SA levels in systemic tissue compared with mock-inoculated plants. In contrast, after treatment with spores of the *MTF1* deletion strain, Pip and SA levels were comparable to water treatment and significantly lower compared with wild-type (*P* < 0.001) or complementation strains (Fig 10). Taken together, these data demonstrate that infection by *V*. *dahliae* requires an intact *MTF1* gene to activate defense responses in tomato plants.

## Discussion

Vta3 exhibits a dual regulatory function important for late stages of the *V*. *dahliae* life cycle, namely plant xylem colonization and resting structure formation for survival in the soil. Fungal pathogens require signaling pathways for successful plant colonization allowing adaptation to changing environmental stimuli, such as host immune responses [71]. *V*. *dahliae* invades through the roots and colonizes the host’s xylem vessels [1,2]. The fungus senses differences in its environment and responds specifically to plant xylem sap by adjusting its secretion [14]. Transcription factors are important downstream elements in the signaling cascades that control fungal cellular responses to the plant environment through expression of genes required for adaptation [71]. Characterization of transcriptional regulators in phytopathogens and identification of their targets therefore contributes to a better understanding of fungal virulence and interactions with host plants.

In this work, we studied Vta3 genetic networks during xylem sap cultivation of *V*. *dahliae*. Vta3 induces expression of *FLO1* and *FLO11* encoding adhesins, thereby rescuing adhesion in a non-adherent yeast strain. In *V*. *dahliae*, *VTA3* is required for root colonization and tomato plant infection. The transcriptional regulator Som1 promotes adhesion, is required earlier during root penetration and induces *VTA3* expression. Som1 and Vta3 both promote conidiation and vegetative growth [17]. Since growth and spore release are crucial for plant colonization [1], these could influence virulence. Nonetheless, Som1 and Vta3 induce gene expression of putative virulence factors, such as Cap20, Nlp2 and the Pr1-like protein Pry1. Vta3 and Som1 also induce gene expression of the transcriptional regulator Vta2, which positively affects conidiation and systemic infection via plant roots [17]. Vta2 affects gene expression of putative secreted proteins and adhesins potentially involved in early plant infection [16]. This study revealed 1,179 genes attributed to various cellular functions that depend on Vta3 for expression (Fig 1A). Vta3 induces gene expression of uncharacterized as well as known candidates, such as the pigment biosynthesis protein Ayg1 [72], the transcription factor Sge1 [73] and the SnodProt1-like protein Cp1 [54], all involved in virulence.

None of the Vta3-dependently reduced transcripts could be assigned a virulence function. However, we identified the transcription factor Mtf1 as a new member reduced in its expression dependent on Vta3 within a network important for disease induction in tomato plants. *MTF1* is dispensable for early colonization and penetration of plant roots but is important in xylem sap for full disease development (Figs 6 and 7). The *MTF1* deletion strain can grow in tomato plants but appears to be less virulent than wild-type. We found no growth or conidiation defect of *MTF1* deletion strains (Figs 5–7 and S9), suggesting that the less severe disease symptoms in tomato plants are due to a specific virulence effect of Mtf1, possibly through regulation of effector gene expression. In this regard, we showed that *AVE1* and *SCP7* expression is induced in xylem sap in the presence of *MTF1* (Fig 9A and 9C). Ave1 increases *V*. *dahliae* aggressiveness on plants lacking the *Ve1* resistance gene. In tomato plants carrying *Ve1*, the receptor-like cell-surface protein Ve1 recognizes Ave1 and triggers a hypersensitive response resulting in resistance. It was hypothesized that Ave1 may increase water flux in the xylem, potentially facilitating host colonization by *V*. *dahliae* [64]. Scp7 is secreted by *V*. *dahliae*, targets the plant nucleus and induces immunity via SA and jasmonate signaling. The authors hypothesize that there may be a plant *R* protein that recognizes Scp7, which triggers an immune response [68]. Infection by biotrophic or hemibiotrophic pathogens such as *V*. *dahliae* usually induces SA signaling as part of plant immunity [74]. A few other *V*. *dahliae* effectors capable of modulating plant immune responses have been described. For example, the glycoside hydrolase 11 family member 424Y, like Scp7, localizes to the host nucleus, where it activates SA and jasmonate signaling [75]. The apoplastic effector Cut11 induces defense responses in *N*. *benthamiana*, cotton and tomato [76]. Scp41 and the fungal isochorismatase Ics1 can suppress plant immunity during host colonization [74,77]. Although Mtf1 is not involved in expression of these effector genes (S10A Fig), plants colonized by the *MTF1* deletion strain show no defense reactions as PR gene expression is not fully induced (Fig 9B) and levels of the plant defense hormones Pip and SA are comparable to uninfected plants (Fig 10). In tomato, lettuce or *A*. *thaliana*, *PR1*, *PR1a*, *PR3* and *PR5* expression levels are elevated after pathogen infection and a defense function is assumed [65–67,78]. According to the ‘growth–defense tradeoff’ hypothesis, plants treated with the *MTF1* deletion strain may also grow taller because they do not invest in defense [79]. *MTF1* transcription is reduced in a Vta3-dependent manner, consequently *VTA3* deletion strains overexpress *MTF1* (Figs 1C and 8A). Plants treated with the *VTA3* deletion strain exhibit no defense reactions (PR gene expression is not induced, S11 Fig), as deletion of *VTA3* leads to a growth defect of these strains on plant roots, resulting in failure already at early infection stages [17].

**Fig 11:**
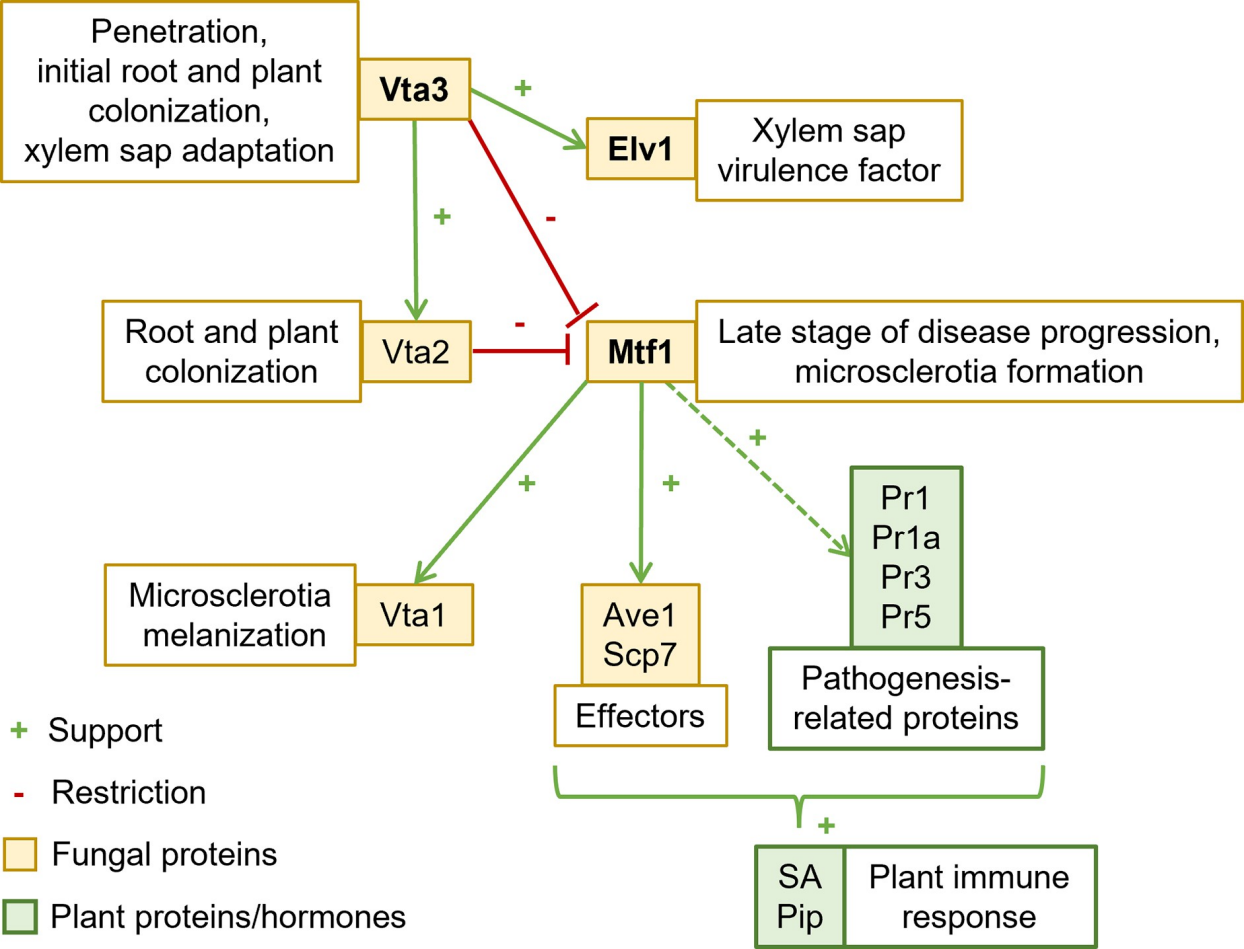
Model of cellular functions and control of the *Verticillium dahliae* Vta-Mtf1 network in microsclerotia formation, plant colonization and immune response. Expression of the transcription factor Mtf1-encoding gene is induced in the absence of the transcriptional regulators Vta3 and Vta2. Vta3 induces *VTA2* expression [17]. Expression of the xylem sap virulence factor Elv1-encoding gene is induced in the presence of *VTA3*. Mtf1 promotes microsclerotia formation and the expression of *VTA1* to favor melanization. Expression of pathogenesis-related protein (PR) genes and levels of phytohormones such as salicylic acid (SA) and pipecolic acid (Pip) in tomato plants are increased in the presence of *MTF1*, supporting plant disease and immune responses. Expression of *AVE1* and *SCP7*, encoding *V*. *dahliae* effectors, is elevated in the presence of *MTF1*.

To our knowledge, a role of Mtf1 in virulence of phytopathogenic fungi has not previously been described. In *Aspergillus* spp., MtfA is known for its functions in morphogenesis and secondary metabolism [56–58]. The cellular functions of Mtf1 in the soil-borne plant pathogen *V*. *dahliae* and its targets are summarized in Fig 11. *MTF1* belongs to a genetic network with the transcriptional regulators Vta3 and Vta2, as the presence of both *VTA3* and *VTA2* reduces *MTF1* transcription (Fig 8). Mtf1 probably acts genetically downstream of Som1, Vta3 and Vta2, whose gene expression is in turn not controlled by Mtf1 (S10B and S10C Fig). Vta3 and Mtf1 also control their own distinct targets. For example, *AVE1* and *SCP7* expression is induced in the presence of *MTF1*, whereas Vta3 does not affect their transcript levels in xylem sap (S12 Fig). Although Mtf1 and Vta3 are both involved in microsclerotia formation (Fig 5) and plant disease (Fig 7), the pathways differ as many factors involved in these processes are affected by Vta3 [17] but not by Mtf1 (S10B and S10C Fig). For example, *VTA3* presence induces gene expression of the Egh16-like virulence factor Elv1 (Fig 1A and 1B), which does not change significantly in the absence or presence of *MTF1* (S10B Fig). Egh16-like virulence factors are found in many pathogenic filamentous fungi (S5A Fig) and are thought to play a role in host interaction. Homologs are present in entomopathogenic fungi [80,81], in *M*. *oryzae* [55] and in the obligate biotrophic fungus *B*. *graminis* f. sp. *hordei*, where their gene expression is induced upon penetration into barley leaves [60,82]. In powdery mildew fungi, different Egh16-like virulence factors are involved to varying degrees in fungal growth, the infection process and host defense. Chitinase activity was suggested as possible mode of action suppressing chitin perception and plant immunity [83]. We found that Elv1, as one of several factors whose gene expression is Vta3-dependently induced in *V*. *dahliae*, enhances tomato plant disease development (Fig 4) without affecting fungal morphogenesis (Fig 3) and PR gene expression (S6 Fig). The global regulator Vta3 is involved in the control of several processes. *VTA3* deletion strains already fail during initial colonization of plant roots [17]. Overexpression of *ELV1* is presumably not sufficient to overcome the virulence deficiency of *VTA3* deletion strains, because an intact *VTA3* gene is required for hyphae to reach the plant xylem sap, where *ELV1* expression is induced.

In summary, we describe *ELV1* and *MTF1* as novel targets of the developmental and pathogenicity regulator Vta3 in the vascular plant pathogen *V*. *dahliae*. Further experiments could help to clarify the complex regulatory networks and the mode of action of the regulatory proteins. For example, possible direct binding of Vta3 or Mtf1 in the promoter region of potential target genes could be investigated using chromatin immunoprecipitation assays. Protein pull-downs were performed with GFP-fused Vta3 to investigate whether there is evidence for a direct interaction of the regulators Vta3 and Mtf1. *V*. *dahliae* was cultured in liquid PDM for five days when both proteins were visible in western experiments (S7 Fig). Ribosomal proteins predominantly co-enriched with Vta3, and the data set did not include Mtf1 as interaction partner of Vta3 (S13 Fig, S10 Table).

The Egh16-like virulence factor Elv1 contributes to virulence, while the regulatory protein Mtf1 is required for late stages of plant infection and host immune responses. Furthermore, Mtf1 promotes microsclerotia formation enabling fungal survival in the soil and re-infection of host plants. Our data indicate that Vta3 is particularly important as a transient factor that initiates processes and terminates others during fungal growth in plant xylem sap and in preparing for survival in the soil after plant death. In future studies, it will be interesting to compare the expression patterns of *ELV1*, *MTF1* and *VTA3* during infection of tomato plants by *V*. *dahliae*. This will require more sensitive approaches, as a first set of pilot experiments with different plant parts and at different time-points after inoculation of the plants with fungal spores resulted in such low levels of fungal RNA compared with plant RNAs that expression of *ELV1*, *MTF1* and *VTA3* could not be detected. Fungal transcription factors are not encoded by highly transcribed genes and can be highly instable [84]. Furthermore, it will be important to determine whether inhibiting Vta3-controlled components, such as Elv1 and Mtf1, leads to development of new strategies reducing both fungal survival in the plant and its decades-long survival in the soil, thereby better mitigating the currently increasing *Verticillium*-caused agricultural damage.

## Supporting information

S1 Fig RNA samples of *Verticillium dahliae* wild-type and *VTA3* deletion strain are more similar within groups than between groups. (DOCX)

S2 Fig Verification of *Verticillium dahliae ELV1* deletion and complementation strains. (DOCX)

S3 Fig Verification of *Verticillium dahliae MTF1* deletion, *MTF1* deletion overexpressing ectopically integrated *GFP* and *MTF1* complementation strains as well as the *GFP-MTF1* strain. (DOCX)

S4 Fig *In silico* discovery of putative Vta3 DNA-binding motifs in the promoters of *ELV1* and *MTF1*. (DOCX)

S5 Fig Comparison of the Egh16-like virulence factor Elv1 and the transcriptional regulator Mtf1 of *Verticillium dahliae* with corresponding proteins from different ascomycetes. (DOCX)

S6 Fig *Verticillium dahliae ELV1* is dispensable for inducing expression of pathogenesis-related protein (PR) genes in tomato plants. (DOCX)

S7 Fig Presence of Mtf1 and Vta3 during growth and development of *V*. *dahliae* in different culture conditions. (DOCX)

S8 Fig *Verticillium dahliae* Mtf1 does not affect *CMR1* expression. (DOCX)

S9 Fig The transcriptional regulator-encoding gene *MTF1* is dispensable for wild-type-like conidiospore levels in *Verticillium dahliae*. (DOCX)

S10 Fig *Verticillium dahliae* Mtf1 does not affect gene expression of several putative targets for virulence, microsclerotia formation and development. (DOCX)

S11 Fig Tomato pathogenesis-related proteins (PR) gene expression is activated after treatment with spores of *Verticillium dahliae* wild-type (WT) but not *VTA3* deletion strain (Δ*VTA3*). (DOCX)

S12 Fig *Verticillium dahliae* Vta3 does not affect the expression of effector genes *AVE1* and *SCP7*. (DOCX)

S13 Fig Proteins interacting with Vta3-GFP during vegetative growth. (DOCX)

S1 Table Primer oligonucleotides used in this study. (DOCX)

S2 Table Plasmids used in this study. (DOCX)

S3 Table Fungal and bacterial strains used in this study. (DOCX)

S4 Table List of Vta3-dependently controlled genes in *Verticillium dahliae* with a log_2_(fold change) ≥ 1 or ≤ -1 found in significantly enriched categories by FunCat analysis. (DOCX)

S5 Table List of *Verticillium dahliae* genes induced in their transcription dependent on Vta3 with a log_2_(fold change) ≤ -1 found in significantly enriched categories by FunCat analysis. (DOCX)

S6 Table List of *Verticillium dahliae* genes reduced in their transcription dependent on Vta3 with a log_2_(fold change) ≥ 1 found in significantly enriched categories by FunCat analysis. (DOCX)

S7 Table List of *Verticillium dahliae* genes induced in their transcription dependent on Vta3 with domains of derived proteins and their putative function. (DOCX)

S8 Table List of *Verticillium dahliae* genes reduced in their transcription dependent on Vta3 with domains of derived proteins and their putative function. (DOCX)

S9 Table qPCR primer oligonucleotides used in this study. (DOCX)

S10 Table Proteins significantly enriched in three replicates of Vta3-GFP versus wild-type with LFQ intensities, MS/MS count, unique peptides, sequence coverage and predicted domains. (DOCX)

S1 Data Raw data for the figures of this study. (XLSX)
