# Supplementary material for: Verticillium dahliae Vta3 promotes ELV1 virulence factor gene expression in xylem sap, but tames Mtf1-mediated late stages of fungus-plant interactions and microsclerotia formation
Source: PLoS Pathog. 2023 Jan 30;19(1):e1011100. doi: 10.1371/journal.ppat.1011100 (PMC9910802; doi:10.1371/journal.ppat.1011100)
Supplement: S1 Fig — (DOCX) [file ppat.1011100.s001.docx]

**S1 Fig**

**
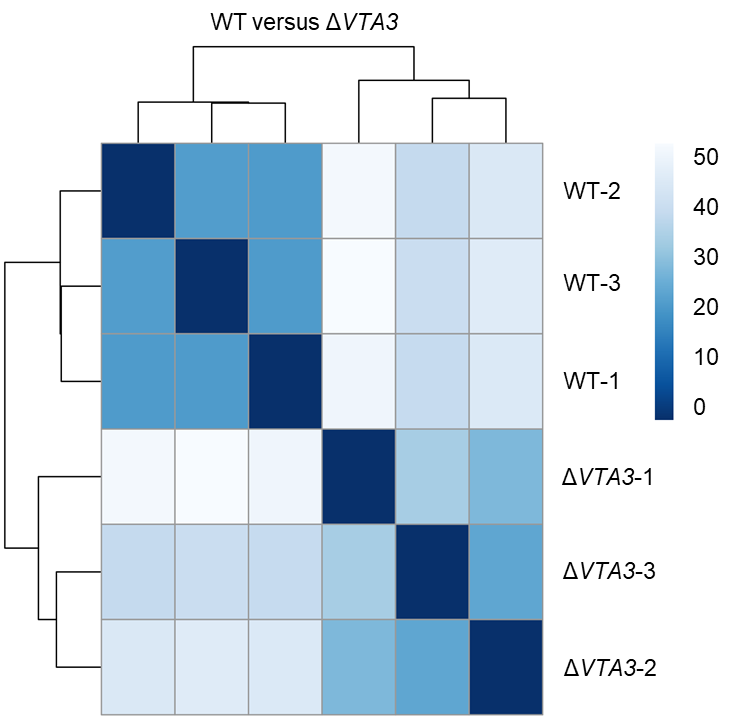
**

**S1 Fig. RNA samples of *Verticillium dahliae* wild-type and *VTA3* deletion strain are more similar within groups than between groups.** Three biological replicates of the JR2 wild-type (WT) and a *VTA3* deletion strain (Δ*VTA3*) were cultured in extracted tomato xylem sap for 8 h following preculture in simulated xylem medium. RNA was extracted from mycelium and sequenced. Sample distances were measured based on the expression values of each sample to determine the similarities within and between groups. The shorter the distance, the more closely related the samples are. Shorter sample distances are represented by darker blue, while lighter blue indicates longer sample distances. The three wild-type replicates are more similar to each other than to the three Δ*VTA3* samples and vice versa*.*
