# Supplementary material for: Verticillium dahliae Vta3 promotes ELV1 virulence factor gene expression in xylem sap, but tames Mtf1-mediated late stages of fungus-plant interactions and microsclerotia formation
Source: PLoS Pathog. 2023 Jan 30;19(1):e1011100. doi: 10.1371/journal.ppat.1011100 (PMC9910802; doi:10.1371/journal.ppat.1011100)
Supplement: S2 Fig — (DOCX) [file ppat.1011100.s002.docx]

**S2 Fig**

**
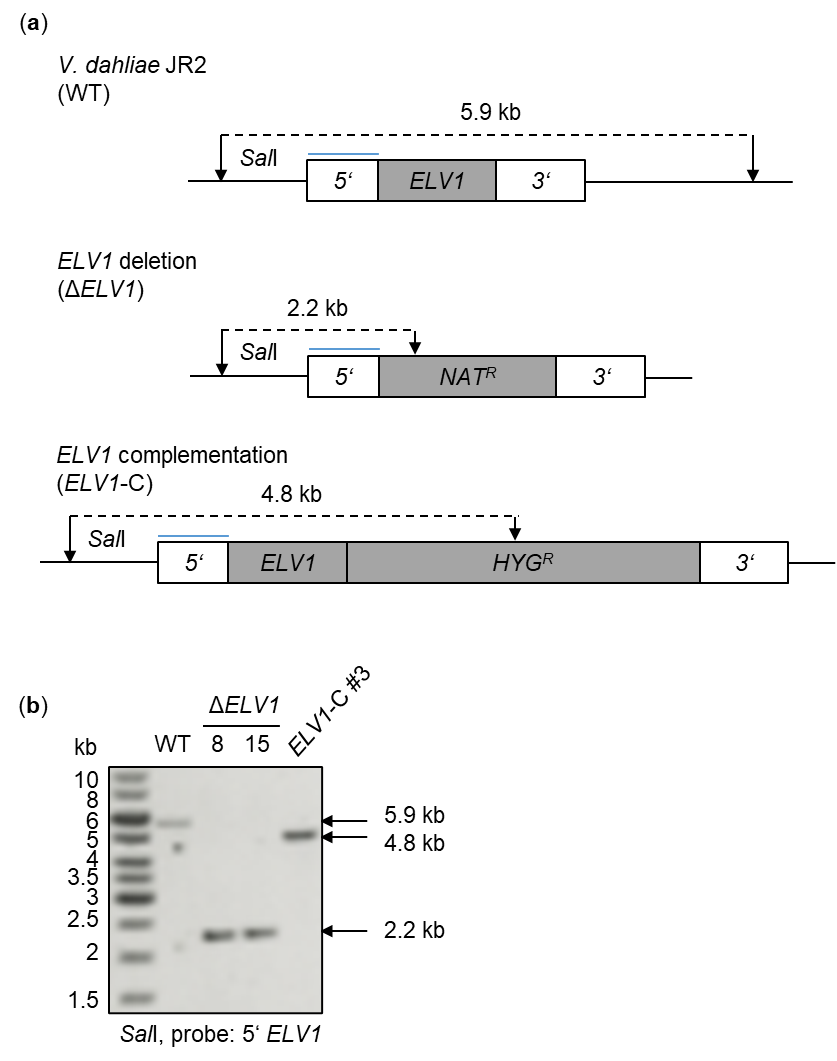
**

*The figure legend is on the next page*.

**S2 Fig. Verification of *Verticillium dahliae ELV1* deletion and complementation strains.** (a) Scheme of restriction sites used for Southern hybridization. *V. dahliae ELV1* deletion (Δ*ELV1*) and complementation (*ELV1*-C) strains were constructed by *Agrobacterium tumefaciens*-mediated transformation. For the deletion strains, the genomic region was replaced against a nourseothricin resistance marker under control of a *gpdA* promoter and a *trpC* terminator (*NAT^R^*) by homologous recombination of the up- and downstream flanking regions. For the complementation strains, the *ELV1* gene together with a hygromycin B resistance marker under control of a *gpdA* promoter and a *trpC* terminator (*HYG^R^*) was reintroduced in the Δ*ELV1* transformant number 8 at the genomic locus. Arrows indicate restriction sites of *Sal*I. The expected fragment lengths labeled with the 5´flanking region as a probe (indicated in blue) are given. (b) Southern hybridization was performed for confirmation of the constructed strains: Δ*ELV1* transformant number 8 (VGB670) and 15 (VGB671) and *ELV1*-C complementation number 3 (VGB694). Genomic DNA of *V. dahliae* JR2 wild-type (WT) served as control. The 5´flanking region of *ELV1* was used as a probe and genomic DNA was cut using the restriction enzyme *Sal*I. The predicted signals corresponding to a fragment length of 5.9 kb for the wild-type, 2.2 kb for the deletion and 4.8 kb for the complementation strain were observed.
