# Supplementary material for: Verticillium dahliae Vta3 promotes ELV1 virulence factor gene expression in xylem sap, but tames Mtf1-mediated late stages of fungus-plant interactions and microsclerotia formation
Source: PLoS Pathog. 2023 Jan 30;19(1):e1011100. doi: 10.1371/journal.ppat.1011100 (PMC9910802; doi:10.1371/journal.ppat.1011100)
Supplement: S3 Fig — (DOCX) [file ppat.1011100.s003.docx]

**S3 Fig**


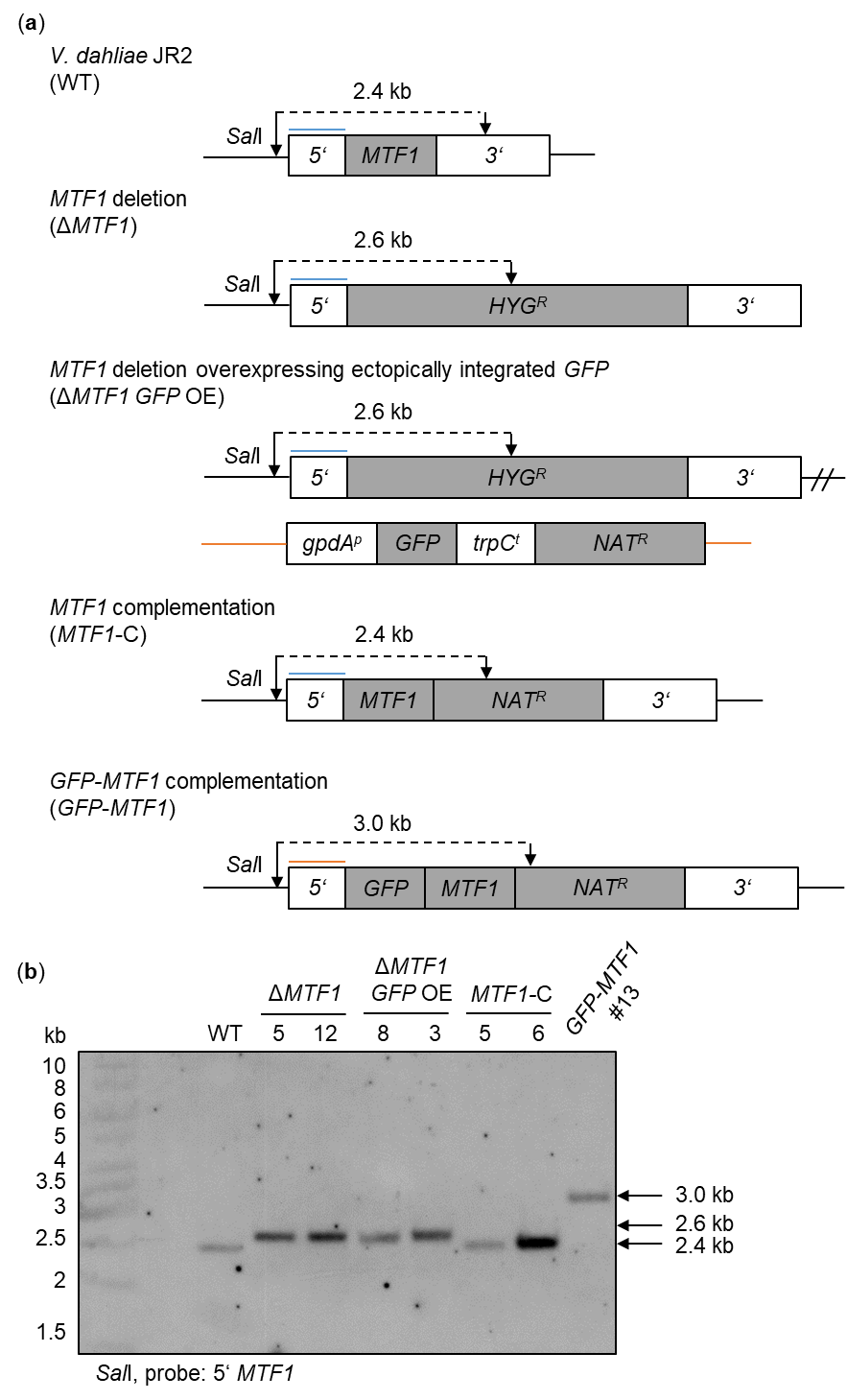


*The figure legend is on the next page*.

**S3 Fig. Verification of *Verticillium dahliae MTF1* deletion, *MTF1* deletion overexpressing ectopically integrated *GFP* and *MTF1* complementation strains as well as the *GFP-MTF1* strain.** (a) Scheme of restriction sites used for Southern hybridization. *V. dahliae MTF1* deletion (Δ*MTF1* and Δ*MTF1 GFP* OE) and complementation (*MTF1*-C and *GFP-MTF1*) strains were constructed by *Agrobacterium tumefaciens*-mediated transformation. In the Δ*MTF1* and Δ*MTF1 GFP* OE strains, the gene is replaced against a hygromycin B resistance marker under control of a *gpdA* promoter and a *trpC* terminator (*HYG^R^*) by homologous recombination of the up- and downstream flanking regions. Ectopic integration sites of *MTF1* deletion strains overexpressing *GFP* (Δ*MTF1 GFP* OE) are visualized by // at the end of the endogenous locus and an orange line for the ectopic locus. *MTF1*-C and *GFP-MTF1* strains were generated by homologous recombination between the *MTF1*-C or *GFP-MTF1* constructs, respectively, and the Δ*MTF1* transformant number 5. The complementation constructs *MTF1*-C and *GFP-MTF1* are driven by the native promotor and terminator and carry the nourseothricin resistance marker under control of a *gpdA* promoter and a *trpC* terminator (*NAT^R^*). Arrows indicate restriction sites of *Sal*I. The expected fragment lengths labeled with the 5´flanking region as a probe (indicated in blue) are given. (b) Southern hybridization was performed for confirmation of the constructed strains: Δ*MTF1* transformant number 5 (VGB575) and 12 (VGB576), Δ*MTF1 GFP* OE transformant number 8 (VGB625) and 3 (VGB626), *MTF1*-C complementation number 5 (VGB635) and 6 (VGB636), and *GFP-MTF1* complementation number 13 (VGB650). Genomic DNA of *V. dahliae* JR2 wild-type (WT) served as control. The 5´flanking region of *MTF1* was used as a probe and genomic DNA was cut using the restriction enzyme *Sal*I. The predicted signals corresponding to a fragment length of 2.4 kb for wild-type and *MTF1*-C, 2.6 kb for the deletion strains and 3.0 kb for *GFP-MTF1* were observed.
