# Supplementary material for: Verticillium dahliae Vta3 promotes ELV1 virulence factor gene expression in xylem sap, but tames Mtf1-mediated late stages of fungus-plant interactions and microsclerotia formation
Source: PLoS Pathog. 2023 Jan 30;19(1):e1011100. doi: 10.1371/journal.ppat.1011100 (PMC9910802; doi:10.1371/journal.ppat.1011100)
Supplement: S4 Fig — (DOCX) [file ppat.1011100.s004.docx]

**S4 Fig**


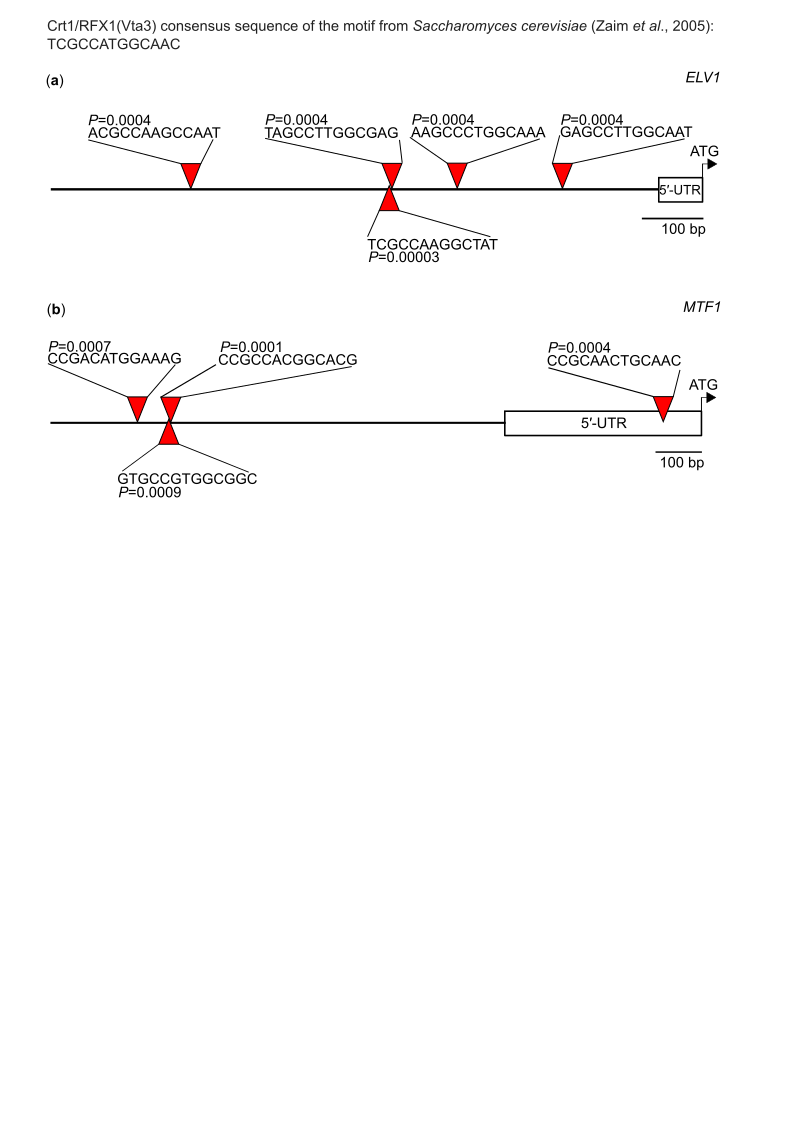


**S4 Fig. *In silico* discovery of putative Vta3 DNA-binding motifs in the promoters of *ELV1* and *MTF1*.** Promoter analysis was performed using the FIMO web tool for (a) *ELV1* and (b) *MTF1*. The motif searched for in both cases was the putative DNA-binding site of Crt1/RFX1 from *Saccharomyces cerevisiae* as the Vta3 counterpart (as discovered by Zaim *et al*., 2005 [1]). Red triangles depict the discovered putative Vta3 motifs in the 5’ regulatory region of both genes with their corresponding *P*-values. The sequences of the corresponding promoter and the 5′ untranslated regions (UTR) were retrieved from Ensembl Fungi.

**References**

1. Zaim J, Speina E, Kierzek AM. Identification of new genes regulated by the Crt1 transcription factor, an effector of the DNA damage checkpoint pathway in *Saccharomyces cerevisiae*. J Biol Chem. 2005;280: 28–37. doi:10.1074/jbc.M404669200
