# Supplementary material for: Verticillium dahliae Vta3 promotes ELV1 virulence factor gene expression in xylem sap, but tames Mtf1-mediated late stages of fungus-plant interactions and microsclerotia formation
Source: PLoS Pathog. 2023 Jan 30;19(1):e1011100. doi: 10.1371/journal.ppat.1011100 (PMC9910802; doi:10.1371/journal.ppat.1011100)
Supplement: S5 Fig — (DOCX) [file ppat.1011100.s005.docx]

**S5 Fig**

**
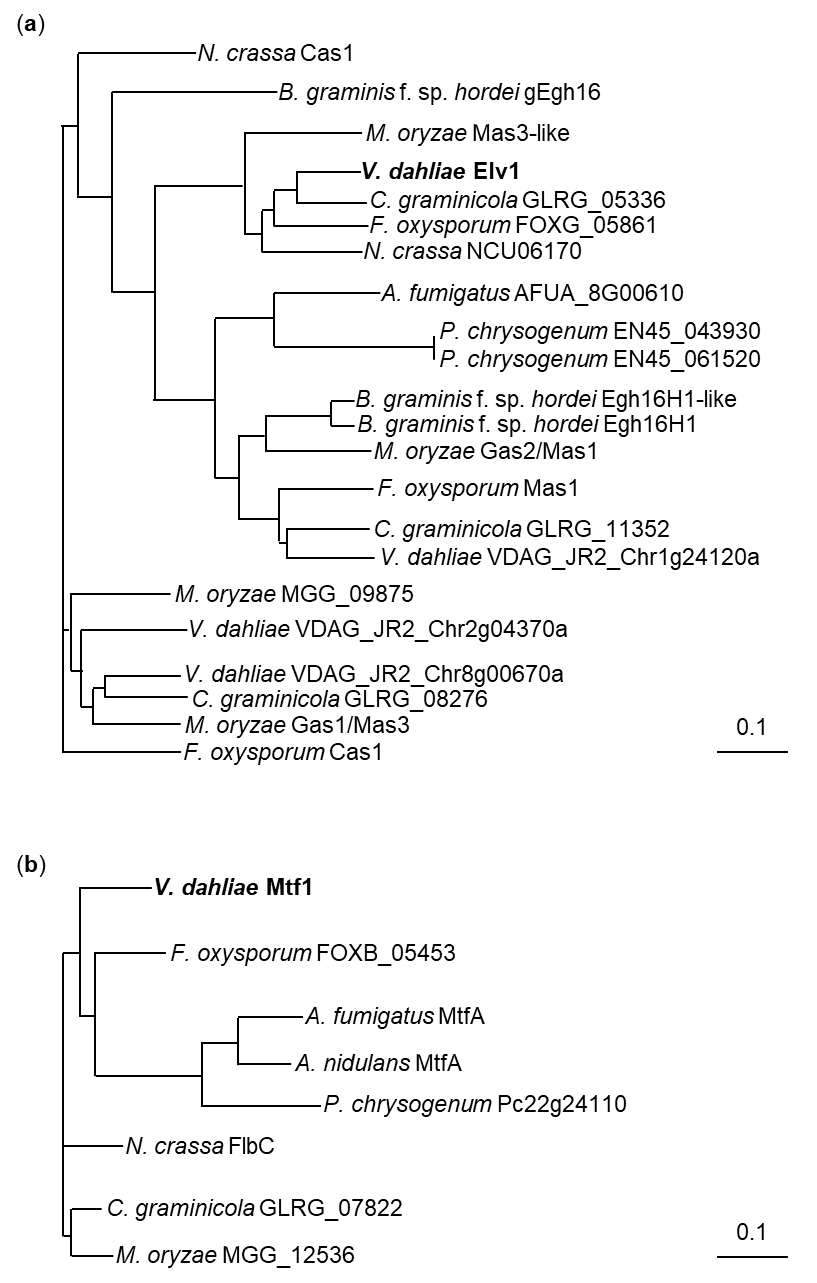
**

*The figure legend is on the next page*.

**S5 Fig. Comparison of the Egh16-like virulence factor Elv1 and the transcriptional regulator Mtf1 of *Verticillium dahliae* with corresponding proteins from different ascomycetes.** A phylogenetic tree was drawn in the MegAlign Pro software using the MUSCLE algorithm for uncorrected pairwise distance alignments. The scale represents the average number of amino acid substitutions per site. (a) Elv1 homologs exist only in filamentous fungi with similarities of 21-66%. The following protein sequences were used: *V. dahliae* Elv1 (VDAG_JR2_Chr6g05120a), VDAG_JR2_Chr1g24120a, VDAG_JR2_Chr2g04370a, VDAG_JR2_Chr8g00670a, *Aspergillus fumigatus* Mas1 (AFUA_8G00610, XP_747144.2), *Blumeria graminis*f. sp. *hordei* Egh16h1 (BLGH_05475), Egh16h1-like (BLGH_05469), gEgh16 (BLGH_01739), *Colletotrichum graminicola* GLRG_05336 (XP_008094212.1), GLRG_08276 (XP_008097152.1), GLRG_11352 (XP_008100227.1), *Fusarium oxysporum* Cas1 (FOXG_10173, XP_018247679.1), Mas1 (FOXG_02712, XP_018236388.1), FOXG_05861, *Magnaporthe oryzae* Cas1 domain-containing MGG_09875 (XP_003720359.1), Gas1/Mas3 (MGG_12337, XP_003715279.1), Gas2/Mas1 (MGG_04202, XP_003719620.1), Mas3-like (MGG_00703, XP_003718303.1), *Neurospora crassa* Cas1 *(*NCU08038*,* XP_962708.1), NCU06170 (XP_960032.1), *Penicillium chrysogenum* EN45_043930 and EN45_061520. (b) For the Mtf1 comparison, the following amino acid sequences were used: *V. dahliae* Mtf1 (VDAG_JR2_Chr2g08470a), *F. oxysporum* FOXB_05453 (EGU84033.1), *A. fumigatus* MtfA (AFUA_6G02690, XP_747808.1), *A. nidulans* MtfA (ANIA_08741, XP_682010.1), *P. chrysogenum* Pc22g24110 (XP_002566301.1), *N. crassa* FlbC (NCU03184, XP_964590.1), *C. graminicola* GLRG_07822 (XP_008096698.1) and *M. oryzae* MGG_12536 (XP_003720663). Protein similarities range from 49-69%. Orthologs do not exist in yeast or humans.
