# Supplementary material for: Verticillium dahliae Vta3 promotes ELV1 virulence factor gene expression in xylem sap, but tames Mtf1-mediated late stages of fungus-plant interactions and microsclerotia formation
Source: PLoS Pathog. 2023 Jan 30;19(1):e1011100. doi: 10.1371/journal.ppat.1011100 (PMC9910802; doi:10.1371/journal.ppat.1011100)
Supplement: S6 Fig — (DOCX) [file ppat.1011100.s006.docx]

**S6 Fig**

**
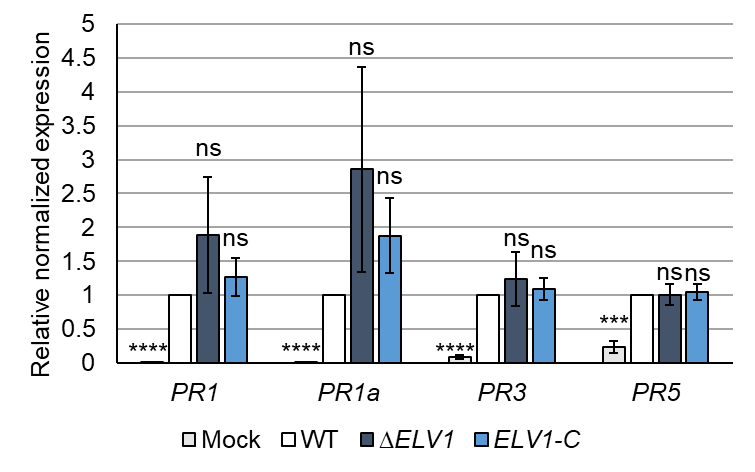
**

**S6 Fig. *Verticillium dahliae ELV1* is dispensable for inducing expression of pathogenesis-related protein (PR) genes in tomato plants.** Transcript levels of tomato PR genes were analyzed by quantitative PCR in hypocotyls 21 days after inoculation with spores of *V. dahliae* JR2 wild-type (WT), *ELV1* deletion (Δ*ELV1*) or complementation (*ELV1-*C) strains. Water-treated plants (mock) served as controls. Hypocotyls of 14 to 15 plants per treatment were pooled (*n* = 1). Shown are the means of six biological replicates from two independent experiments, with error bars representing the SE of the mean. Normalization to transcript levels of wild-type and references *EF1α* and *αTUB* was performed. Significant differences from wild-type were determined by *t*-test. The expression levels of *PR1*, *PR1a*, *PR3* and *PR5* were not significantly different between wild-type, deletion or complementation strain-infected plants (ns, not significant). In plants treated with water, the expression of PR genes was significantly lower (***, *P* < 0.001; ****, *P*< 0.0001).
