# Supplementary material for: Verticillium dahliae Vta3 promotes ELV1 virulence factor gene expression in xylem sap, but tames Mtf1-mediated late stages of fungus-plant interactions and microsclerotia formation
Source: PLoS Pathog. 2023 Jan 30;19(1):e1011100. doi: 10.1371/journal.ppat.1011100 (PMC9910802; doi:10.1371/journal.ppat.1011100)
Supplement: S7 Fig — (DOCX) [file ppat.1011100.s007.docx]

**S7 Fig**

**
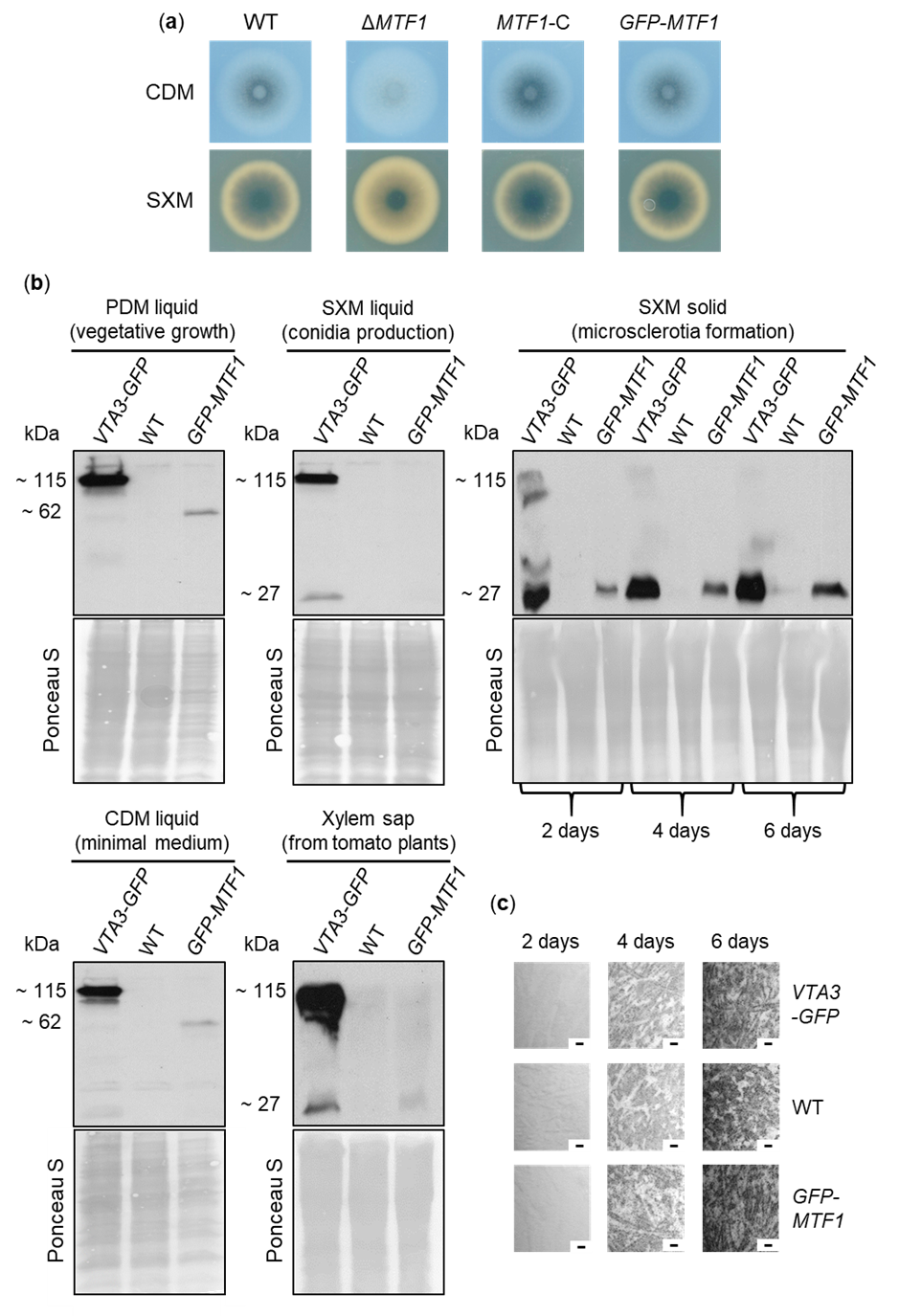
**

*The figure legend is on the next page*.

**S7 Fig. Presence of Mtf1 and Vta3 during growth and development of *V*. *dahliae* in different culture conditions.** (a) Plates containing Czapek-Dox medium (CDM) or simulated xylem medium (SXM) were point-inoculated with 50,000 spores of *V. dahliae* wild-type (WT), *MTF1* deletion (Δ*MTF1*), *MTF1* complementation (*MTF1*-C) and *GFP-MTF1* expressing (*GFP-MTF1*) strains and incubated at 25 °C for 10 days. Bottom view scans show that the complementation and *GFP-MTF1* expressing strains resemble the wild-type, while the *MTF1* deletion strain appears less melanized. (b) *V. dahliae VTA3-GFP*, *GFP-MTF1* and wild-type strains were cultured in potato dextrose medium (PDM) to stimulate vegetative growth, in liquid SXM to promote conidia production or on solid SXM (plates covered by a nylon membrane) for microsclerotia formation. CDM was used as minimal medium and xylem sap from tomato plants was used to reflect the real situation inside the plant host. The media were inoculated with 5 x 10^7^ fresh spores, and the mycelia were harvested after five days and 8 h. For solid SXM, mycelia were harvested after two, four and six days. For xylem sap, mycelia were precultured in SXM for five days, then transferred to xylem sap and incubated for an additional 8 h, which corresponded to the culture conditions used for the RNA sequencing experiment. Western hybridization was performed with an α-GFP antibody to detect Vta3 or Mtf1 fused to GFP. Ponceau S staining served as a loading control. Extracts from the wild-type were used as negative controls. Signals corresponding to Vta3-GFP (115 kDa) were detected in PDM, liquid SXM, CDM, xylem sap and on solid SXM after two days. Signals corresponding to GFP-Mtf1 (62 kDa) were detected only in PDM and CDM. Free GFP (27 kDa) was detected when *V. dahliae* grew on solid SXM. (c) Spores of indicated strains were spread on SXM plates covered with nylon membrane and incubated at 25 °C for the stated times. Binocular images demonstrate increasing amount of microsclerotia over time, which is similar for all strains (scale = 500 µm).
