# Supplementary material for: Verticillium dahliae Vta3 promotes ELV1 virulence factor gene expression in xylem sap, but tames Mtf1-mediated late stages of fungus-plant interactions and microsclerotia formation
Source: PLoS Pathog. 2023 Jan 30;19(1):e1011100. doi: 10.1371/journal.ppat.1011100 (PMC9910802; doi:10.1371/journal.ppat.1011100)
Supplement: S8 Fig — (DOCX) [file ppat.1011100.s008.docx]

**S8 Fig**

**
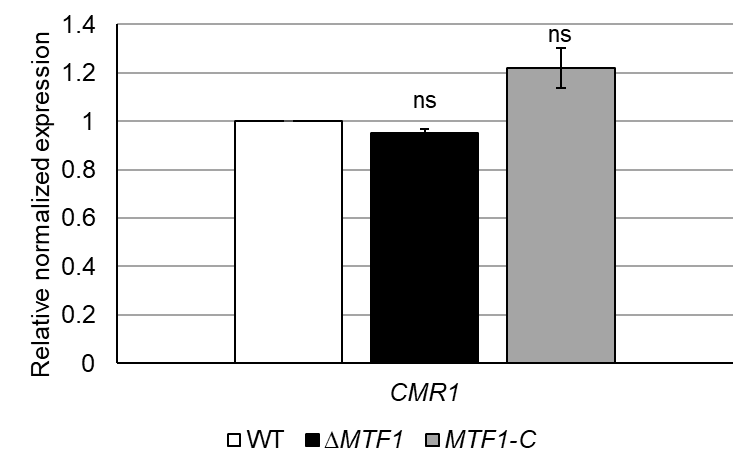
**

**S8 Fig. *Verticillium dahliae* Mtf1 does not affect *CMR1* expression*.*** Transcript levels were analyzed by quantitative PCR in JR2 wild-type (WT), *MTF1* deletion (Δ*MTF1*) and complementation (*MTF1*-C) strains cultured in extracted tomato xylem sap for 8 h following preculture in simulated xylem medium. Transcript levels of references *H2A* and *EIF2B* were used for normalization and *CMR1* expression in wild-type was set to one. Means of three independent experiments ± SE of the mean are shown. Expression of *CMR1*, a gene coding for the melanin cluster-regulating transcription factor Cmr1, did not change significantly in the absence or presence of *MTF1* (ns, not significant, calculated using *t*-test).
