# Supplementary material for: Verticillium dahliae Vta3 promotes ELV1 virulence factor gene expression in xylem sap, but tames Mtf1-mediated late stages of fungus-plant interactions and microsclerotia formation
Source: PLoS Pathog. 2023 Jan 30;19(1):e1011100. doi: 10.1371/journal.ppat.1011100 (PMC9910802; doi:10.1371/journal.ppat.1011100)
Supplement: S9 Fig — (DOCX) [file ppat.1011100.s009.docx]

**S9 Fig**

**
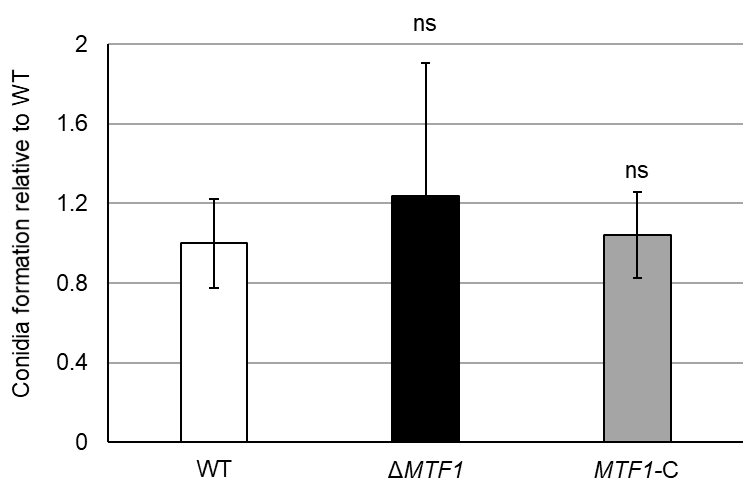
**

**S9 Fig. The transcriptional regulator-encoding gene *MTF1* is dispensable for wild-type-like conidiospore levels in *Verticillium dahliae*.** Conidia formation was quantified in five-day-old cultures in liquid simulated xylem medium incubated at 25 °C with constant agitation after inoculation of 4,000 spores ml^-1^. Significance was calculated using *t*-tests (ns, not significant). Three independent experiments were performed with two biological replicates (*n* = 6) and three technical replicates each. Error bars represent the SD of the mean values. No difference in the ability to form conidiospores was observed between wild-type (WT), *MTF1* deletion (Δ*MTF1*) and *MTF1* complementation (*MTF1*-C) strains.
