# Supplementary material for: Verticillium dahliae Vta3 promotes ELV1 virulence factor gene expression in xylem sap, but tames Mtf1-mediated late stages of fungus-plant interactions and microsclerotia formation
Source: PLoS Pathog. 2023 Jan 30;19(1):e1011100. doi: 10.1371/journal.ppat.1011100 (PMC9910802; doi:10.1371/journal.ppat.1011100)
Supplement: S10 Fig — (DOCX) [file ppat.1011100.s010.docx]

**S10 Fig**

**
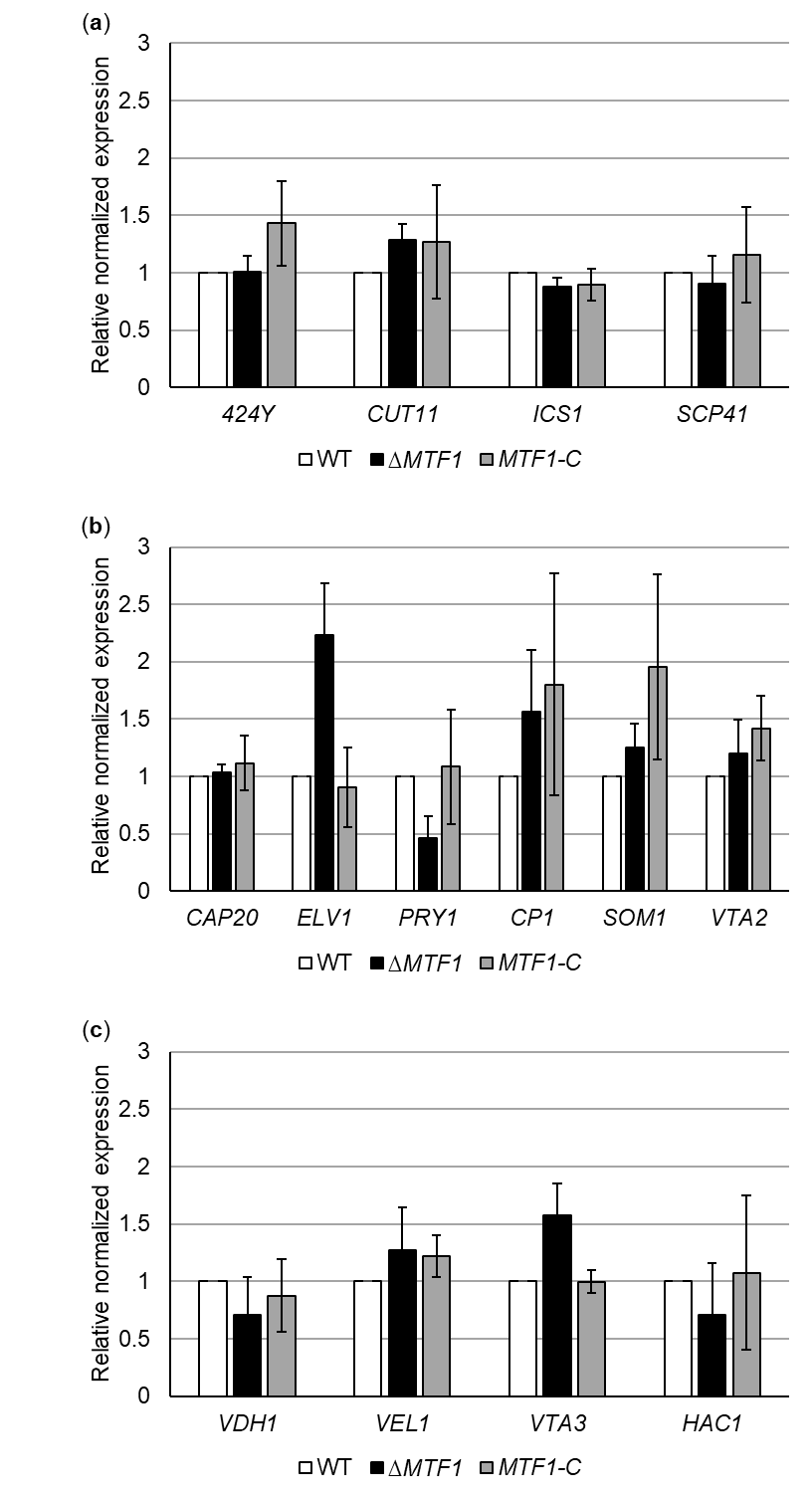
**

*The figure legend is on the next page*.

**S10 Fig. *Verticillium dahliae* Mtf1 does not affect gene expression of several putative targets for virulence, microsclerotia formation and development.** Transcript levels were analyzed by quantitative PCR in JR2 wild-type (WT), *MTF1* deletion (Δ*MTF1*) and *MTF1* complementation (*MTF1*-C) strains. The strains were cultured in extracted tomato xylem sap for 8 h following preculture in simulated xylem medium. Transcript levels of references *H2A* and *EIF2B* were used for normalization and gene expression in wild-type was set to one. Significant differences from wild-type were determined by *t*-test. Means of three independent experiments are shown with error bars indicating the SE of the mean. Mtf1 does not control expression of several putative targets for (a) plant immunity [1,2,3] and (b) virulence [4,5,6,7,8]. (c) Expression levels of *VDH1* for microsclerotia formation [9] and *VEL1*, *VTA3* and *HAC1* for development and virulence [5,10,11] are not significantly affected by the absence or presence of *MTF1*.

**References**

1. Liu L, Wang Z, Li J, Wang Y, Yuan J, Zhan J, et al. *Verticillium dahliae* secreted protein Vd424Y is required for full virulence, targets the nucleus of plant cells, and induces cell death. Mol Plant Pathol. 2021;22: 1109–1120. doi:10.1111/mpp.13100
2. Gui Y-J, Zhang W-Q, Zhang D-D, Zhou L, Short DPG, Wang J, et al. A *Verticillium dahliae* extracellular cutinase modulates plant immune responses. Mol Plant Microbe Interact. 2018;31: 260–273. doi:10.1094/MPMI-06-17-0136-R
3. Qin J, Wang K, Sun L, Xing H, Wang S, Li L, et al. The plant-specific transcription factors CBP60g and SARD1 are targeted by a *Verticillium* secretory protein VdSCP41 to modulate immunity. Elife. 2018;7: e34902. doi:10.7554/eLife.34902.001
4. Tran V-T, Braus-Stromeyer SA, Kusch H, Reusche M, Kaever A, Kühn A, et al. *Verticillium* transcription activator of adhesion Vta2 suppresses microsclerotia formation and is required for systemic infection of plant roots. New Phytol. 2014;202: 565–581. doi:10.1111/nph.12671
5. Bui T-T, Harting R, Braus-Stromeyer SA, Tran V-T, Leonard M, Höfer A, et al. *Verticillium dahliae* transcription factors Som1 and Vta3 control microsclerotia formation and sequential steps of plant root penetration and colonisation to induce disease. New Phytol. 2019;221: 2138–2159. doi:10.1111/nph.15514
6. Zhang Y, Gao Y, Liang Y, Dong Y, Yang X, Yuan J, et al. The *Verticillium dahliae* SnodProt1-like protein VdCP1 contributes to virulence and triggers the plant immune system. Front Plant Sci. 2017;8: 1880. doi:10.3389/fpls.2017.01880
7. Hwang C-S, Flaishman MA, Kolattukudy PE. Cloning of a gene expressed during appressorium formation by *Colletotrichum gloeosporioides* and a marked decrease in virulence by disruption of this gene. Plant Cell. 1995;7: 183–193. doi:10.1105/tpc.7.2.183
8. Prados-Rosales RC, Roldán-Rodríguez R, Serena C, López-Berges MS, Guarro J, Martínez-del-Pozo Á, et al. A PR-1-like protein of *Fusarium oxysporum* functions in virulence on mammalian hosts. J Biol Chem. 2012;287: 21970–21979. doi:10.1074/jbc.M112.364034
9. Klimes A, Dobinson KF. A hydrophobin gene, *VDH1*, is involved in microsclerotial development and spore viability in the plant pathogen *Verticillium dahliae*. Fungal Genetics and Biology. 2006;43: 283–294. doi:10.1016/j.fgb.2005.12.006
10. Höfer AM, Harting R, Aßmann NF, Gerke J, Schmitt K, Starke J, et al. The velvet protein Vel1 controls initial plant root colonization and conidia formation for xylem distribution in Verticillium wilt. PLoS Genet. 2021;17: e1009434. doi:10.1371/journal.pgen.1009434
11. Starke J, Harting R, Maurus I, Leonard M, Bremenkamp R, Heimel K, et al. Unfolded protein response and scaffold independent pheromone MAP kinase signaling control *Verticillium dahliae* growth, development, and plant pathogenesis. J Fungi (Basel). 2021;7: 305. doi:10.3390/jof7040305
