# Supplementary material for: Verticillium dahliae Vta3 promotes ELV1 virulence factor gene expression in xylem sap, but tames Mtf1-mediated late stages of fungus-plant interactions and microsclerotia formation
Source: PLoS Pathog. 2023 Jan 30;19(1):e1011100. doi: 10.1371/journal.ppat.1011100 (PMC9910802; doi:10.1371/journal.ppat.1011100)
Supplement: S11 Fig — (DOCX) [file ppat.1011100.s011.docx]

**S11 Fig**

**
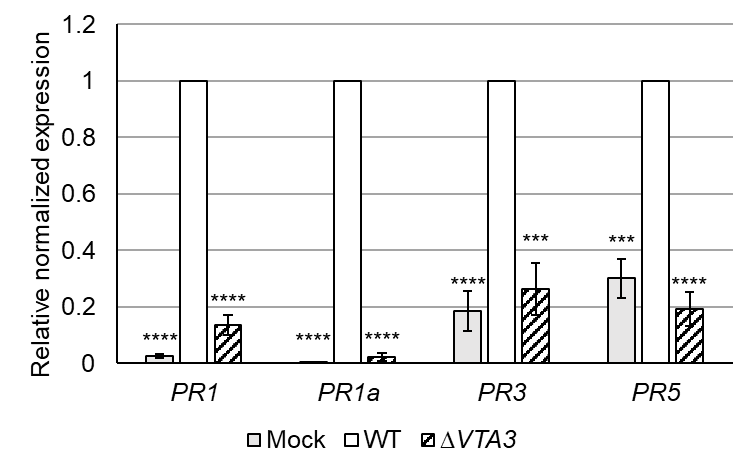
**

**S11 Fig.** **Tomato pathogenesis-related proteins (PR) gene expression is activated after treatment with spores of *Verticillium dahliae* wild-type (WT) but not *VTA3* deletion strain (Δ*VTA3*).** Transcript levels of PR genes were analyzed by quantitative PCR in tomato hypocotyls 21 days after inoculation. Water-treated plants (mock) served as controls. Hypocotyls of 14 to 15 plants per treatment were pooled (*n* = 1). Shown are the means of six biological replicates from three independent experiments, with error bars representing the SE of the mean. Normalization to transcript levels of wild-type and references *EF1α* and *αTUB* was performed. Significant differences from wild-type were determined by *t*-test. Expression levels of *PR1*, *PR1a*, *PR3* and *PR5* were significantly lower in plants treated with water or Δ*VTA3* compared with wild-type-infected plants (***, *P* < 0.001; ****, *P*< 0.0001).
