# Supplementary material for: Verticillium dahliae Vta3 promotes ELV1 virulence factor gene expression in xylem sap, but tames Mtf1-mediated late stages of fungus-plant interactions and microsclerotia formation
Source: PLoS Pathog. 2023 Jan 30;19(1):e1011100. doi: 10.1371/journal.ppat.1011100 (PMC9910802; doi:10.1371/journal.ppat.1011100)
Supplement: S12 Fig — (DOCX) [file ppat.1011100.s012.docx]

**S12 Fig**

**
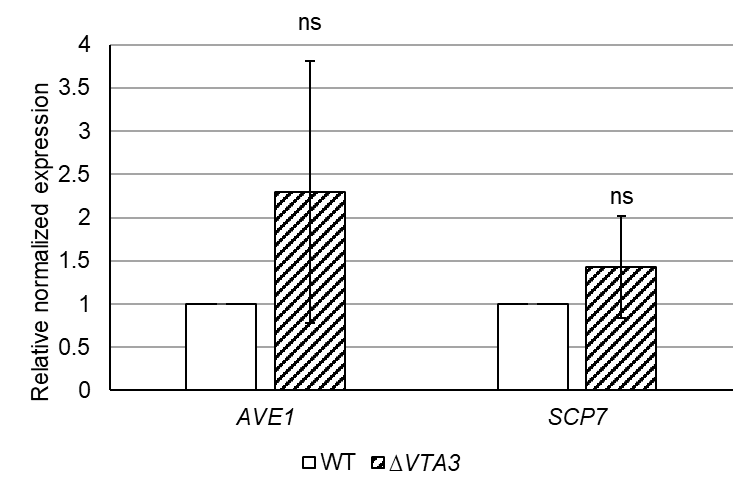
**

**S12 Fig. *Verticillium dahliae* Vta3 does not affect the expression of effector genes *AVE1* and *SCP7.*** Gene expression was analyzed by quantitative PCR in JR2 wild-type (WT) and a *VTA3* deletion strain (Δ*VTA3*). Strains were cultured in extracted tomato xylem sap for 8 h following preculture in simulated xylem medium. Transcript levels of references *H2A* and *EIF2B* were used for normalization and gene expression in wild-type was set to one. Means of three independent experiments ± SE of the mean are shown. Although expression levels of *AVE1* and *SCP7* were elevated in the absence of *VTA3*, calculation by *t*-test did not reveal a statistically significant difference compared with wild-type (ns, not significant).
