# Supplementary material for: Verticillium dahliae Vta3 promotes ELV1 virulence factor gene expression in xylem sap, but tames Mtf1-mediated late stages of fungus-plant interactions and microsclerotia formation
Source: PLoS Pathog. 2023 Jan 30;19(1):e1011100. doi: 10.1371/journal.ppat.1011100 (PMC9910802; doi:10.1371/journal.ppat.1011100)
Supplement: S13 Fig — (DOCX) [file ppat.1011100.s013.docx]

**S13 Fig**

**
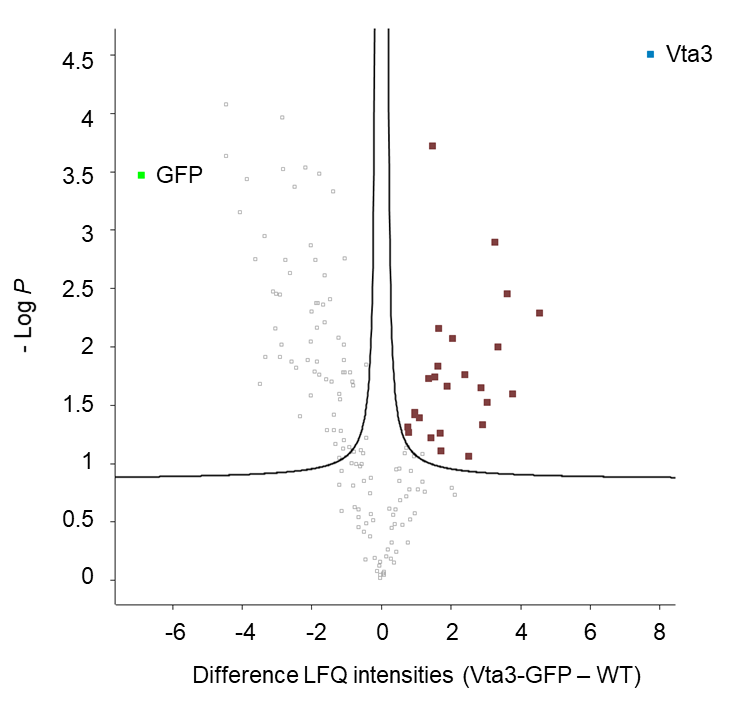
**

**S13 Fig. Proteins interacting with Vta3-GFP during vegetative growth.** Spores of the *V. dahliae* wild-type, the wild-type overexpressing ectopically integrated *GFP* and the *VTA3-GFP* expressing strain were grown in liquid potato-dextrose medium for five days. Ground mycelia of the wild-type strain and the wild-type strain overexpressing ectopically integrated *GFP* were mixed before protein extraction (2/3 wild-type and 1/3 wild-type strain overexpressing ectopically integrated *GFP*). Proteins were subjected to GFP-trap pull-downs and digested with trypsin. The resulting peptides were analyzed by LC/MS. The Volcano plot depicts the mean of three independent replicates. LFQ intensity differences of proteins identified with the Vta3-GFP fusion protein compared with wild-type (WT) are shown on the x-axis and -Log *P* values of the *t*-test on the y-axis. The replacement of missing values by imputed values and the subsequent *t*-test were performed independently four times to obtain reliable candidates of interaction. The Volcano plot displays the result of one of these four repetitions. The 25 proteins found to be significantly co-enriched with Vta3 in all four repetitions are highlighted in brown. Vta3 is highlighted in blue. Free GFP (as control) is indicated in light green. Further details can be found in S10 Table.
