# Supplementary material for: Verticillium dahliae Vta3 promotes ELV1 virulence factor gene expression in xylem sap, but tames Mtf1-mediated late stages of fungus-plant interactions and microsclerotia formation
Source: PLoS Pathog. 2023 Jan 30;19(1):e1011100. doi: 10.1371/journal.ppat.1011100 (PMC9910802; doi:10.1371/journal.ppat.1011100)
Supplement: S1 Table — (DOCX) [file ppat.1011100.s014.docx]

**S1 Table. Primer oligonucleotides used in this study.**

| **Primer name** | **Primer sequence (5’ → 3’)** | **Length (-mer)** | **Overhang to** |
| --- | --- | --- | --- |
| IM32 | **ATT CTT AAT TAA GAT** TAC CTA ATT AAT CAC CAG AAA | 36 | pME4564 |
| IM34 | **AGG TAA TCC TTC TTT** GCG CCG AAT ATT CAT ATT | 33 | *trpC^t^* |
| IM35 | **AGG ACT TCT AGA AGG** GTT CAC GCA GGT ACA GTC | 33 | pME4564 |
| IM40 | **ATT CTT AAT TAA GAT** CAG GGC TAC CCA CCT ACA GTC | 36 | pME4564 |
| IM41 | **AGA TCC CCG GGT ACC** GAT GTC CGG CAC TGG CGC | 33 | *^p^gpdA* |
| IM42 | **AGG TAA TCC TTC TTT** TTC GGC AGC GGA GAC GTG | 33 | *trpC^t^* |
| IM43 | **AGG ACT TCT AGA AGG** TCT CGC CAC TTG GGA TGA | 33 | pME4564 |
| IM84 | **ACC GGT CAC TGT ACA** TCA GGA GAC GGG CGC CG | 32 | *^p^gpdA* |
| IM85 | **GGT GAA AGA AAC CAT** TTT CTA TGT GTT CAA GGA TGT | 36 | *ELV1* gene |
| IM86 | ATG GTT TCT TTC ACC GAT TTC | 21 | ^-^ |
| IM91 | **GGT GGT AGC GGT GGT** ATG CAT CAC TAC ATC AGC AAC ACC | 39 | Linker with *GFP* |
| IM92 | **GCC CTT GCT CAC CAT** GAT GTC CGG CAC TGG CGC | 33 | *GFP* |
| IM105 | **ACC GGT CAC TGT ACA** TTT CTA TGT GTT CAA GGA TGT | 36 | *^p^gpdA* |
| IM143 | **AGA TCC CCG GGT ACC** TTA TGC CAT CCT GTT AGA C | 34 | *^p^gpdA* |
| ML5 | TGTACAGTGACCGGTGACTCTT | 22 | - |
| ML8 | AAA GAA GGA TTA CCT CTA AAC AA | 23 | - |
| ML9 | TGT ACA GTG ACC GGT GAC | 18 | - |
| RO3 | GGT ACC CGG GGA TCT TTC G | 19 | - |
| ZQY10 | ATGGTGAGCAAGGGCGAG | 18 | - |
| ZQY11 | ACCACCGCTACCACCCTTGTACAGCTCGTCCATGC | 35 | - |

*^p^*: promoter, *^t^*: terminator; bold: overhangs for Seamless Cloning.
