# Supplementary material for: Verticillium dahliae Vta3 promotes ELV1 virulence factor gene expression in xylem sap, but tames Mtf1-mediated late stages of fungus-plant interactions and microsclerotia formation
Source: PLoS Pathog. 2023 Jan 30;19(1):e1011100. doi: 10.1371/journal.ppat.1011100 (PMC9910802; doi:10.1371/journal.ppat.1011100)
Supplement: S2 Table — (DOCX) [file ppat.1011100.s015.docx]

**S2 Table. Plasmids used in this study.**

| **Plasmid name** | **Description** | **Reference** |
| --- | --- | --- |
| pGreen2 | *^p^gpdA:GFP:trpC^t^:^p^gpdA:HYG^R^:trpC^t^; KAN^R^* | [1] |
| pPK2 | Cloning vector with *KAN^R^* and *HYG^R^*; left and right border for *Agrobacterium tumefaciens*-mediated transformation | [2] |
| pME4564 | Cloning vector with *KAN^R^* and *HYG^R^*; left and right border for *A. tumefaciens*-mediated transformation | [3] |
| pME4815 | *^p^gpdA:NAT^R^:trpC^t^* in pME4564 | [3] |
| pME4819 | *^p^*gpdA:*GFP*:trpC*^t^* in pME4815 | [4] |
| pME5480 | *^p^MTF1:^p^gpdA:HYG^R^:trpC^t^:MTF1^t^* in pME4564 | This study |
| pME5481 | *^p^MTF1:MTF1:^p^gpdA:NAT^R^:trpC^t^:MTF1^t^* in pME4564 | This study |
| pME5482 | *^p^ELV1:^p^gpdA:NAT^R^:trpC^t^:ELV1^t^* in pME4564 | This study |
| pME5483 | *^p^ELV1:ELV1:^p^gpdA:HYG^R^:trpC^t^:ELV1^t^* in pME4564 | This study |
| pME5510 | *^p^MTF1:GFP:MTF1:^p^gpdA:NAT^R^:trpC^t^:MTF1^t^*  in pME4564 | This study |

^p^: promoter, ^t^: terminator, HYG^R^: hygromycin B resistance marker, *KAN^R^*: kanamycin resistance marker, NAT^R^: nourseothricin resistance marker.

**References**

1. Tran V-T, Braus-Stromeyer SA, Kusch H, Reusche M, Kaever A, Kühn A, et al. *Verticillium* transcription activator of adhesion Vta2 suppresses microsclerotia formation and is required for systemic infection of plant roots. New Phytol. 2014;202: 565–581. doi:10.1111/nph.12671
2. Covert SF, Kapoor P, Lee M, Briley A, Nairn CJ. *Agrobacterium tumefaciens*-mediated transformation of *Fusarium circinatum*. Mycol Res. 2001;105: 259–264. doi:10.1017/S0953756201003872
3. Leonard M, Kühn A, Harting R, Maurus I, Nagel A, Starke J, et al. *Verticillium longisporum* elicits media-dependent secretome responses with capacity to distinguish between plant-related environments. Front Microbiol. 2020;11: 1876. doi:10.3389/fmicb.2020.01876
4. Starke J, Harting R, Maurus I, Leonard M, Bremenkamp R, Heimel K, et al. Unfolded protein response and scaffold independent pheromone MAP kinase signaling control *Verticillium dahliae* growth, development, and plant pathogenesis. J Fungi (Basel). 2021;7: 305. doi:10.3390/jof7040305
