# Supplementary material for: Verticillium dahliae Vta3 promotes ELV1 virulence factor gene expression in xylem sap, but tames Mtf1-mediated late stages of fungus-plant interactions and microsclerotia formation
Source: PLoS Pathog. 2023 Jan 30;19(1):e1011100. doi: 10.1371/journal.ppat.1011100 (PMC9910802; doi:10.1371/journal.ppat.1011100)
Supplement: S3 Table — (DOCX) [file ppat.1011100.s016.docx]

**S3 Table. Fungal and bacterial strains used in this study.**

| **Strain name** | **Description** | **Reference** |
| --- | --- | --- |
| ***Agrobacterium tumefaciens*** | | |
| AGL1 | Strain for *Agrobacterium tumefaciens*-mediated transformation of *Verticillium dahliae* | [1] |
| ***Escherichia coli*** | | |
| DH5α | Strain for cloning and extraction of plasmids | Invitrogen Thermo Fisher Scientific |
| ***Verticillium dahliae*** | | |
| JR2/WT | *Solanum lycopersicum* isolate | [2] |
| WT *GFP* OE  (VGB45/  VGB392) | JR2 overexpressing ectopically integrated *GFP* (*^p^gpdA:GFP:trpC^t^:^p^gpdA:HYG^R^:trpC^t^*/ *^p^gpdA:GFP:trpC^t^:^p^gpdA:NAT^R^:trpC^t^*) | [3,4] |
| Δ*SOM1*  (VGB1/  VGB2) | *SOM1* deletion strain (Δ*SOM1::^p^trpC:NAT^R^*) | [5] |
| Δ*VTA3*  (VGB9) | *VTA3* deletion strain (Δ*VTA3::^p^trpC:NAT^R^*) | [5] |
| Δ*VTA2*  (VGB88) | *VTA2* deletion strain (Δ*VTA2::^p^gpdA:NAT^R^*) | [3] |
| Δ*VTA3 GFP* OE  (VGB184/ VGB185) | *VTA3* deletion strain overexpressing ectopically integrated *GFP* (Δ*VTA3:: ^p^trpC:NAT^R^,* *^p^gpdA:GFP:trpC^t^:^p^gpdA:HYG^R^:trpC^t^*) | [5] |
| *VTA3-GFP*  (VGB284) | *VTA3* deletion strain expression ectopically integrated *VTA3-GFP* (Δ*VTA3::^p^trpC:NAT^R^, ^p^VTA3:VTA3:GFP:trpC^t^*, *^p^gpdA:HYG^R^:trpC^t^*) | [5] |
| Δ*MTF1*  (VGB575/  VGB576) | *MTF1* deletion strain (Δ*MTF1::^p^gpdA:HYG^R^:trpC^t^*) | This study |
| Δ*MTF1 GFP* OE  (VGB625/  VGB626) | *MTF1* deletion strain overexpressing ectopically integrated *GFP* (Δ*MTF1::^p^gpdA:HYG^R^:trpC^t^, ^p^gpdA:GFP:trpC^t^:^p^gpdA:NAT^R^:trpC^t^*) | This study |
| *MTF1*-C  (VGB635/  VGB636) | *MTF1* complementation strain (Δ*MTF1::^p^MTF1:MTF1:^p^gpdA:NAT^R^:trpC^t^:MTF1^t^*) | This study |
| *GFP-MTF1* (VGB650) | *GFP-MTF1 expressing strain* (Δ*MTF1::^p^MTF1:GFP:MTF1:^p^gpdA:NAT^R^:trpC^t^:MTF1^t^*) | This study |
| Δ*ELV1*  (VGB670/  VGB671) | *ELV1* deletion strain (Δ*ELV1::^p^gpdA:NAT^R^:trpC^t^*) | This study |
| *ELV1*-C  (VGB694) | *ELV1* complementation strain (Δ*ELV1::^p^ELV1:ELV1:^p^gpdA:HYG^R^:trpC^t^:ELV1^t^*) | This study |

^p^: promoter, ^t^: terminator, HYG^R^: hygromycin B resistance marker, NAT^R^: nourseothricin resistance marker; two VGB numbers for one genotype indicate two independent transformants.

**References**

1. Lazo GR, Stein PA, Ludwig RA. A DNA transformation-competent *Arabidopsis* genomic library in *Agrobacterium*. Biotechnology (N Y). 1991;9: 963–967. doi:10.1038/nbt1091-963
2. Fradin EF, Zhang Z, Juarez Ayala JC, Castroverde CDM, Nazar RN, Robb J, et al. Genetic dissection of Verticillium wilt resistance mediated by tomato Ve1. Plant Physiol. 2009;150: 320–332. doi:10.1104/pp.109.136762
3. Tran V-T, Braus-Stromeyer SA, Kusch H, Reusche M, Kaever A, Kühn A, et al. *Verticillium* transcription activator of adhesion Vta2 suppresses microsclerotia formation and is required for systemic infection of plant roots. New Phytol. 2014;202: 565–581. doi:10.1111/nph.12671
4. Starke J, Harting R, Maurus I, Leonard M, Bremenkamp R, Heimel K, et al. Unfolded protein response and scaffold independent pheromone MAP kinase signaling control *Verticillium dahliae* growth, development, and plant pathogenesis. J Fungi (Basel). 2021;7: 305. doi:10.3390/jof7040305
5. Bui T-T, Harting R, Braus-Stromeyer SA, Tran V-T, Leonard M, Höfer A, et al. *Verticillium dahliae* transcription factors Som1 and Vta3 control microsclerotia formation and sequential steps of plant root penetration and colonisation to induce disease. New Phytol. 2019;221: 2138–2159. doi:10.1111/nph.15514
