# Supplementary material for: Verticillium dahliae Vta3 promotes ELV1 virulence factor gene expression in xylem sap, but tames Mtf1-mediated late stages of fungus-plant interactions and microsclerotia formation
Source: PLoS Pathog. 2023 Jan 30;19(1):e1011100. doi: 10.1371/journal.ppat.1011100 (PMC9910802; doi:10.1371/journal.ppat.1011100)
Supplement: S4 Table — (DOCX) [file ppat.1011100.s017.docx]

**S4 Table. List of Vta3-dependently controlled genes in *Verticillium dahliae* with a log_2_(fold change) ≥ 1 or ≤ -1 found in significantly enriched categories by FunCat analysis.**

| **Gene** **identifier** | **Protein name** | **FunCat description** | **log_2_(fold change)** |
| --- | --- | --- | --- |
| *VDAG_JR2_Chr1g11720a* | Putative uncharacterized protein | Disease, virulence and defense | 1.09 |
| *VDAG_JR2_Chr1g12490a* | Putative uncharacterized protein | Disease, virulence and defense | 1.02 |
| *VDAG_JR2_Chr2g02970a* | Integral membrane protein | Disease, virulence and defense | -1.16 |
| *VDAG_JR2_Chr2g05500a* | CFEM domain-containing protein | Disease, virulence and defense | 1.99 |
| *VDAG_JR2_Chr2g07000a* | SnodProt1 (Cp2) | Disease, virulence and defense | -1.36 |
| *VDAG_JR2_Chr2g11980a* | Sulfate adenylyltransferase (EC 2.7.7.4) (ATP-sulfurylase) (Sulfate adenylate transferase) | Disease, virulence and defense | -2.05 |
| *VDAG_JR2_Chr3g09940a* | Endo-1,3(4)-beta-glucanase | Disease, virulence and defense | -3.96 |
| *VDAG_JR2_Chr3g11660a* | Integral membrane protein | Disease, virulence and defense | -1.20 |
| *VDAG_JR2_Chr4g04680a* | L-aminoadipate-semialdehyde dehydrogenase large subunit | Disease, virulence and defense | 1.09 |
| *VDAG_JR2_Chr4g05930a* | Putative uncharacterized protein | Disease, virulence and defense | -1.09 |
| *VDAG_JR2_Chr4g11930a* | Integral membrane protein | Disease, virulence and defense | -1.44 |
| *VDAG_JR2_Chr5g03450a* | Putative uncharacterized protein | Disease, virulence and defense | -1.56 |
| *VDAG_JR2_Chr5g03870a* | Putative uncharacterized protein | Disease, virulence and defense | -1.08 |
| *VDAG_JR2_Chr6g10960a* | Indoleamine 2,3-dioxygenase family protein | Disease, virulence and defense | -1.14 |
| *VDAG_JR2_Chr7g00860a* | SnodProt1 (Cp1) | Disease, virulence and defense | -1.45 |
| *VDAG_JR2_Chr7g02720a* | Integral membrane protein | Disease, virulence and defense | -2.64 |
| *VDAG_JR2_Chr8g05600a* | Integral membrane protein | Disease, virulence and defense | -2.36 |
| *VDAG_JR2_Chr8g10640a* | Integral membrane protein | Disease, virulence and defense | -1.86 |
| *VDAG_JR2_Chr8g11120a* | Integral membrane protein | Disease, virulence and defense | -1.59 |
| *VDAG_JR2_Chr1g00545a* | AN1-type zinc finger protein | Heavy metal binding (Cu, Fe, Zn) | -2.30 |
| *VDAG_JR2_Chr1g01670a* | Cytosine-specific methyltransferase (EC 2.1.1.37) | Heavy metal binding (Cu, Fe, Zn) | 1.49 |
| *VDAG_JR2_Chr1g01700a* | Alkaline phosphatase (EC 3.1.3.1) | Heavy metal binding (Cu, Fe, Zn) | 2.06 |
| *VDAG_JR2_Chr1g02360a* | Histone deacetylase clr3 | Heavy metal binding (Cu, Fe, Zn) | 1.00 |
| *VDAG_JR2_Chr1g02550a* | Cytochrome c | Heavy metal binding (Cu, Fe, Zn) | -1.11 |
| *VDAG_JR2_Chr1g05880a* | Coproporphyrinogen III oxidase | Heavy metal binding (Cu, Fe, Zn) | -1.53 |
| *VDAG_JR2_Chr1g07420a* | Sentrin-specific protease | Heavy metal binding (Cu, Fe, Zn) | 1.05 |
| *VDAG_JR2_Chr1g08890a* | Putative uncharacterized protein | Heavy metal binding (Cu, Fe, Zn) | 1.15 |
| *VDAG_JR2_Chr1g09730a* | Pre-mRNA-splicing factor ATP-dependent RNA helicase PRP43 | Heavy metal binding (Cu, Fe, Zn) | 1.00 |
| *VDAG_JR2_Chr1g11070a* | Zinc finger protein | Heavy metal binding (Cu, Fe, Zn) | 1.87 |
| *VDAG_JR2_Chr1g11370a* | Cytochrome P450 61 | Heavy metal binding (Cu, Fe, Zn) | -1.01 |
| *VDAG_JR2_Chr1g12840a* | Delta-aminolevulinic acid dehydratase (EC 4.2.1.24) | Heavy metal binding (Cu, Fe, Zn) | -1.01 |
| *VDAG_JR2_Chr1g14700a* | Cytochrome P450 3A2 | Heavy metal binding (Cu, Fe, Zn) | -1.52 |
| *VDAG_JR2_Chr1g16460a* | C-5 sterol desaturase | Heavy metal binding (Cu, Fe, Zn) | -1.21 |
| *VDAG_JR2_Chr1g19700a* | Sorbitol dehydrogenase | Heavy metal binding (Cu, Fe, Zn) | 1.22 |
| *VDAG_JR2_Chr1g21000a* | Paraplegin | Heavy metal binding (Cu, Fe, Zn) | 1.03 |
| *VDAG_JR2_Chr1g22770a* | Neutral protease 2 (EC 3.4.24.39) (Deuterolysin) | Heavy metal binding (Cu, Fe, Zn) | -1.95 |
| *VDAG_JR2_Chr1g23020a* | RING finger protein | Heavy metal binding (Cu, Fe, Zn) | -1.22 |
| *VDAG_JR2_Chr1g26350a* | Ferric reductase | Heavy metal binding (Cu, Fe, Zn) | 1.28 |
| *VDAG_JR2_Chr1g27940a* | Polyamine oxidase | Heavy metal binding (Cu, Fe, Zn) | -1.24 |
| *VDAG_JR2_Chr1g29660a* | Zinc finger protein | Heavy metal binding (Cu, Fe, Zn) | 1.15 |
| *VDAG_JR2_Chr2g00060a* | TRI15 protein | Heavy metal binding (Cu, Fe, Zn) | 1.09 |
| *VDAG_JR2_Chr2g01550a* | Fatty acid desaturase | Heavy metal binding (Cu, Fe, Zn) | -1.08 |
| *VDAG_JR2_Chr2g01930a* | Ubiquitin carboxyl-terminal hydrolase | Heavy metal binding (Cu, Fe, Zn) | -1.09 |
| *VDAG_JR2_Chr2g03540a* | STAM binding protein | Heavy metal binding (Cu, Fe, Zn) | 1.36 |
| *VDAG_JR2_Chr2g04930a* | Ferric reductase transmembrane component 5 | Heavy metal binding (Cu, Fe, Zn) | 1.48 |
| *VDAG_JR2_Chr2g05690a* | Branchpoint-bridging protein | Heavy metal binding (Cu, Fe, Zn) | 1.18 |
| *VDAG_JR2_Chr2g08470a* | Putative uncharacterized protein | Heavy metal binding (Cu, Fe, Zn) | 2.08 |
| *VDAG_JR2_Chr2g10530a* | Integrin beta-1-binding protein | Heavy metal binding (Cu, Fe, Zn) | 3.52 |
| *VDAG_JR2_Chr2g11640a* | Sister chromatid cohesion protein Eso1 | Heavy metal binding (Cu, Fe, Zn) | 1.10 |
| *VDAG_JR2_Chr2g12410a* | RING-9 protein | Heavy metal binding (Cu, Fe, Zn) | -1.18 |
| *VDAG_JR2_Chr3g00970a* | Putative uncharacterized protein | Heavy metal binding (Cu, Fe, Zn) | -1.81 |
| *VDAG_JR2_Chr3g01800a* | Cytochrome c1 | Heavy metal binding (Cu, Fe, Zn) | -1.03 |
| *VDAG_JR2_Chr3g02560a* | Cytochrome P450 51 | Heavy metal binding (Cu, Fe, Zn) | -1.51 |
| *VDAG_JR2_Chr3g03520a* | Chaperone protein dnaJ 2 | Heavy metal binding (Cu, Fe, Zn) | -2.10 |
| *VDAG_JR2_Chr3g04790a* | Putative uncharacterized protein | Heavy metal binding (Cu, Fe, Zn) | 1.86 |
| *VDAG_JR2_Chr3g08650a* | Tyrosinase | Heavy metal binding (Cu, Fe, Zn) | -1.04 |
| *VDAG_JR2_Chr3g09680a* | Nitrite reductase | Heavy metal binding (Cu, Fe, Zn) | -1.04 |
| *VDAG_JR2_Chr3g10110a* | Farnesyltransferase subunit beta | Heavy metal binding (Cu, Fe, Zn) | 1.08 |
| *VDAG_JR2_Chr4g02640a* | Alcohol dehydrogenase | Heavy metal binding (Cu, Fe, Zn) | -1.36 |
| *VDAG_JR2_Chr4g02930a* | DNA damage-responsive transcriptional repressor RPH1 | Heavy metal binding (Cu, Fe, Zn) | -1.07 |
| *VDAG_JR2_Chr4g05680a* | Inositolphosphorylceramide-B C-26 hydroxylase | Heavy metal binding (Cu, Fe, Zn) | -1.61 |
| *VDAG_JR2_Chr4g06310a* | RING-2 protein | Heavy metal binding (Cu, Fe, Zn) | 1.89 |
| *VDAG_JR2_Chr4g07690a* | Cytochrome c oxidase polypeptide VIb | Heavy metal binding (Cu, Fe, Zn) | -1.22 |
| *VDAG_JR2_Chr4g07740a* | C-4 methylsterol oxidase | Heavy metal binding (Cu, Fe, Zn) | -1.40 |
| *VDAG_JR2_Chr4g08020a* | Putative uncharacterized protein | Heavy metal binding (Cu, Fe, Zn) | 1.29 |
| *VDAG_JR2_Chr4g08190a* | Acyl-CoA desaturase | Heavy metal binding (Cu, Fe, Zn) | -1.79 |
| *VDAG_JR2_Chr4g10510a* | Putative uncharacterized protein | Heavy metal binding (Cu, Fe, Zn) | -1.75 |
| *VDAG_JR2_Chr4g10750a* | Homogentisate 1,2-dioxygenase | Heavy metal binding (Cu, Fe, Zn) | -1.10 |
| *VDAG_JR2_Chr4g11050a* | Carboxypeptidase B | Heavy metal binding (Cu, Fe, Zn) | -1.48 |
| *VDAG_JR2_Chr5g01660a* | Zinc carboxypeptidase A | Heavy metal binding (Cu, Fe, Zn) | -1.51 |
| *VDAG_JR2_Chr5g01690a* | Amine oxidase | Heavy metal binding (Cu, Fe, Zn) | -1.12 |
| *VDAG_JR2_Chr5g09240a* | DNA ligase (EC 6.5.1.1) | Heavy metal binding (Cu, Fe, Zn) | 1.28 |
| *VDAG_JR2_Chr6g00360a* | Calpain clp-1 | Heavy metal binding (Cu, Fe, Zn) | 1.53 |
| *VDAG_JR2_Chr6g02750a* | DNA polymerase (EC 2.7.7.7) | Heavy metal binding (Cu, Fe, Zn) | -1.13 |
| *VDAG_JR2_Chr6g03470a* | Sphingolipid C4-hydroxylase SUR2 | Heavy metal binding (Cu, Fe, Zn) | -1.32 |
| *VDAG_JR2_Chr6g08940a* | Cytochrome P450 | Heavy metal binding (Cu, Fe, Zn) | 1.02 |
| *VDAG_JR2_Chr6g10960a* | Indoleamine 2,3-dioxygenase family protein | Heavy metal binding (Cu, Fe, Zn) | -1.14 |
| *VDAG_JR2_Chr7g02680a* | DNA polymerase kappa | Heavy metal binding (Cu, Fe, Zn) | 1.69 |
| *VDAG_JR2_Chr7g03720a* | Mitochondrial-processing peptidase subunit beta | Heavy metal binding (Cu, Fe, Zn) | -1.02 |
| *VDAG_JR2_Chr7g04380a* | Cytochrome b-245 heavychain subunit beta | Heavy metal binding (Cu, Fe, Zn) | 1.05 |
| *VDAG_JR2_Chr7g04540a* | Maintenance of ploidy protein mob1 | Heavy metal binding (Cu, Fe, Zn) | -1.10 |
| *VDAG_JR2_Chr7g05330a* | S-(Hydroxymethyl)glutathione dehydrogenase | Heavy metal binding (Cu, Fe, Zn) | 1.34 |
| *VDAG_JR2_Chr7g05410a* | Ubiquitin carboxyl-terminal hydrolase (EC 3.4.19.12) | Heavy metal binding (Cu, Fe, Zn) | 1.04 |
| *VDAG_JR2_Chr7g05810a* | Heavy metal tolerance protein | Heavy metal binding (Cu, Fe, Zn) | -1.66 |
| *VDAG_JR2_Chr7g06940a* | C-5 sterol desaturase | Heavy metal binding (Cu, Fe, Zn) | -1.90 |
| *VDAG_JR2_Chr8g03350a* | Reticulon-4-interacting protein | Heavy metal binding (Cu, Fe, Zn) | 2.24 |
| *VDAG_JR2_Chr8g03620a* | Alcohol dehydrogenase | Heavy metal binding (Cu, Fe, Zn) | 1.08 |
| *VDAG_JR2_Chr8g10150a* | NAD-dependent deacetylase sirtuin-7 | Heavy metal binding (Cu, Fe, Zn) | 2.68 |
| *VDAG_JR2_Chr1g02550a* | Cytochrome c | Heme binding | -1.11 |
| *VDAG_JR2_Chr1g11370a* | Cytochrome P450 61 | Heme binding | -1.01 |
| *VDAG_JR2_Chr1g14700a* | Cytochrome P450 3A2 | Heme binding | -1.52 |
| *VDAG_JR2_Chr1g17930a* | Nitrate reductase | Heme binding | 1.09 |
| *VDAG_JR2_Chr2g01550a* | Fatty acid desaturase | Heme binding | -1.08 |
| *VDAG_JR2_Chr3g01800a* | Cytochrome c1 | Heme binding | -1.03 |
| *VDAG_JR2_Chr3g02560a* | Cytochrome P450 51 | Heme binding | -1.51 |
| *VDAG_JR2_Chr4g05680a* | Inositolphosphorylceramide-B C-26 hydroxylase | Heme binding | -1.61 |
| *VDAG_JR2_Chr4g07690a* | Cytochrome c oxidase polypeptide VIb | Heme binding | -1.22 |
| *VDAG_JR2_Chr4g10780a* | Cytochrome P450 76A2 | Heme binding | -1.91 |
| *VDAG_JR2_Chr6g02570a* | Catalase (EC 1.11.1.6) | Heme binding | 1.72 |
| *VDAG_JR2_Chr6g08530a* | Ent-kaurene oxidase | Heme binding | 1.39 |
| *VDAG_JR2_Chr6g08940a* | Cytochrome P450 | Heme binding | 1.02 |
| *VDAG_JR2_Chr6g10960a* | Indoleamine 2,3-dioxygenase family protein | Heme binding | -1.14 |
| *VDAG_JR2_Chr7g04380a* | Cytochrome b-245 heavychain subunit beta | Heme binding | 1.05 |
| *VDAG_JR2_Chr1g04280a* | NAD(P)H-dependent D-xylose reductase | Secondary metabolism | 1.49 |
| *VDAG_JR2_Chr1g04920a* | Kynurenine 3-monooxygenase (EC 1.14.13.9) (Biosynthesis of nicotinic acid protein 4) (Kynurenine 3-hydroxylase) | Secondary metabolism | -1.02 |
| *VDAG_JR2_Chr1g05220a* | Putative uncharacterized protein | Secondary metabolism | 1.50 |
| *VDAG_JR2_Chr1g05420a* | Putative uncharacterized protein | Secondary metabolism | -1.05 |
| *VDAG_JR2_Chr1g10990a* | Putative uncharacterized protein | Secondary metabolism | 1.03 |
| *VDAG_JR2_Chr1g14700a* | Cytochrome P450 3A2 | Secondary metabolism | -1.52 |
| *VDAG_JR2_Chr1g17080a* | IBR finger domain-containing protein | Secondary metabolism | -1.19 |
| *VDAG_JR2_Chr1g17090a* | Putative uncharacterized protein | Secondary metabolism | 1.27 |
| *VDAG_JR2_Chr1g17200a* | Conidial yellow pigment biosynthesis polyketide synthase | Secondary metabolism | 1.44 |
| *VDAG_JR2_Chr1g22830a* | Allantoate permease | Secondary metabolism | 1.27 |
| *VDAG_JR2_Chr1g23310a* | Putative uncharacterized protein | Secondary metabolism | 1.00 |
| *VDAG_JR2_Chr1g23320a* | 4-nitrophenylphosphatase | Secondary metabolism | 1.00 |
| *VDAG_JR2_Chr1g24600a* | 4-hydroxyacetophenone monooxygenase | Secondary metabolism | -3.10 |
| *VDAG_JR2_Chr1g24800a* | Ent-kaurene oxidase | Secondary metabolism | -1.60 |
| *VDAG_JR2_Chr1g25130a* | Glyoxylate reductase | Secondary metabolism | 1.06 |
| *VDAG_JR2_Chr1g25460a* | Aflatoxin B1 aldehyde reductase member 3 | Secondary metabolism | 2.85 |
| *VDAG_JR2_Chr1g26700a* | 2-ketogluconate reductase | Secondary metabolism | 1.06 |
| *VDAG_JR2_Chr1g26890a* | Pleiotropic ABC multiple drug efflux transporter | Secondary metabolism | 1.11 |
| *VDAG_JR2_Chr1g27940a* | Polyamine oxidase | Secondary metabolism | -1.24 |
| *VDAG_JR2_Chr1g28250a* | FAD binding domain-containing protein | Secondary metabolism | -1.87 |
| *VDAG_JR2_Chr1g28420a* | Polyamine transporter 3 | Secondary metabolism | -1.39 |
| *VDAG_JR2_Chr1g29150a* | Glyoxalase/bleomycin resistance protein/dioxygenase | Secondary metabolism | 1.09 |
| *VDAG_JR2_Chr2g00060a* | TRI15 protein | Secondary metabolism | 1.09 |
| *VDAG_JR2_Chr2g02180a* | Riboflavin transporter MCH5 | Secondary metabolism | -2.04 |
| *VDAG_JR2_Chr2g03090a* | ATP-dependent permease MDL1 | Secondary metabolism | 1.06 |
| *VDAG_JR2_Chr2g03180a* | Epoxide hydrolase | Secondary metabolism | 2.04 |
| *VDAG_JR2_Chr2g04160a* | 4-coumarate-CoA ligase | Secondary metabolism | 1.07 |
| *VDAG_JR2_Chr2g04760a* | TRI14 protein | Secondary metabolism | -2.42 |
| *VDAG_JR2_Chr2g06040a* | Putative uncharacterized protein | Secondary metabolism | 1.47 |
| *VDAG_JR2_Chr2g08250a* | O-acetylhomoserine (Thiol)-lyase | Secondary metabolism | 1.69 |
| *VDAG_JR2_Chr2g08790a* | Ferulic acid esterase A | Secondary metabolism | 1.21 |
| *VDAG_JR2_Chr3g00980a* | Aflatoxin biosynthesis ketoreductase nor-1 | Secondary metabolism | -1.10 |
| *VDAG_JR2_Chr3g02560a* | Cytochrome P450 51 | Secondary metabolism | -1.51 |
| *VDAG_JR2_Chr3g08570a* | Ankyrin repeat protein | Secondary metabolism | 1.35 |
| *VDAG_JR2_Chr3g08640a* | Putative uncharacterized protein | Secondary metabolism | -1.52 |
| *VDAG_JR2_Chr3g09580a* | Putative uncharacterized protein | Secondary metabolism | -1.34 |
| *VDAG_JR2_Chr3g09770a* | Siderophore iron transporter mirA | Secondary metabolism | -1.29 |
| *VDAG_JR2_Chr3g10150a* | Phenazine biosynthesis protein | Secondary metabolism | -2.31 |
| *VDAG_JR2_Chr3g10430a* | Lactose permease | Secondary metabolism | -1.05 |
| *VDAG_JR2_Chr3g11120a* | Putative uncharacterized protein | Secondary metabolism | -1.14 |
| *VDAG_JR2_Chr4g00210a* | Bilirubin oxidase | Secondary metabolism | -1.66 |
| *VDAG_JR2_Chr4g01630a* | Aerobactin siderophore biosynthesis protein iucB | Secondary metabolism | -1.11 |
| *VDAG_JR2_Chr4g02070a* | Polyamine transporter 1 | Secondary metabolism | 1.56 |
| *VDAG_JR2_Chr4g02260a* | Putative uncharacterized protein | Secondary metabolism | -1.09 |
| *VDAG_JR2_Chr4g02590a* | Cytochrome P450 52A11 | Secondary metabolism | -1.62 |
| *VDAG_JR2_Chr4g03020a* | Bifunctional P-450:NADPH-P450 reductase | Secondary metabolism | 1.62 |
| *VDAG_JR2_Chr4g03530a* | Siderophore iron transporter mirB | Secondary metabolism | 2.01 |
| *VDAG_JR2_Chr4g03830a* | Putative uncharacterized protein | Secondary metabolism | 4.00 |
| *VDAG_JR2_Chr4g04340a* | Putative uncharacterized protein | Secondary metabolism | -1.25 |
| *VDAG_JR2_Chr4g04640a* | Aromatic and neutral aliphatic amino acid permease | Secondary metabolism | -1.15 |
| *VDAG_JR2_Chr4g04680a* | L-aminoadipate-semialdehyde dehydrogenase large subunit | Secondary metabolism | 1.09 |
| *VDAG_JR2_Chr4g05940a* | Alpha-glucosides permease MPH2/3 | Secondary metabolism | -2.13 |
| *VDAG_JR2_Chr4g06340a* | Putative uncharacterized protein | Secondary metabolism | 1.14 |
| *VDAG_JR2_Chr4g06660a* | Fumarylacetoacetate hydrolase domain-containing protein | Secondary metabolism | 1.25 |
| *VDAG_JR2_Chr4g06920a* | Putative uncharacterized protein | Secondary metabolism | -1.50 |
| *VDAG_JR2_Chr4g09680a* | Putative uncharacterized protein | Secondary metabolism | 1.04 |
| *VDAG_JR2_Chr4g09790a* | Endoglucanase-1 | Secondary metabolism | -1.02 |
| *VDAG_JR2_Chr4g10620a* | Salicylate hydroxylase | Secondary metabolism | -3.31 |
| *VDAG_JR2_Chr4g10730a* | Stress responsive A/B barrel domain-containing protein | Secondary metabolism | -1.82 |
| *VDAG_JR2_Chr4g10760a* | Choline transport protein | Secondary metabolism | -1.64 |
| *VDAG_JR2_Chr4g10770a* | Amine oxidase | Secondary metabolism | -1.06 |
| *VDAG_JR2_Chr4g10780a* | Cytochrome P450 76A2 | Secondary metabolism | -1.91 |
| *VDAG_JR2_Chr4g10990a* | Sulphydryl oxidase | Secondary metabolism | -1.06 |
| *VDAG_JR2_Chr4g11360a* | Quinate permease | Secondary metabolism | 1.35 |
| *VDAG_JR2_Chr4g11610a* | Putative uncharacterized protein | Secondary metabolism | -1.03 |
| *VDAG_JR2_Chr4g11940a* | Alpha/beta hydrolase | Secondary metabolism | -1.00 |
| *VDAG_JR2_Chr5g00970a* | Choline monooxygenase | Secondary metabolism | 1.26 |
| *VDAG_JR2_Chr5g01070a* | Multidrug resistance protein | Secondary metabolism | 2.46 |
| *VDAG_JR2_Chr5g01440a* | FAD binding domain-containing protein | Secondary metabolism | 1.58 |
| *VDAG_JR2_Chr5g01690a* | Amine oxidase | Secondary metabolism | -1.12 |
| *VDAG_JR2_Chr5g02800a* | Putative uncharacterized protein | Secondary metabolism | 1.83 |
| *VDAG_JR2_Chr5g03130a* | Glucose oxidase | Secondary metabolism | -1.34 |
| *VDAG_JR2_Chr5g03740a* | Lactose permease | Secondary metabolism | -1.19 |
| *VDAG_JR2_Chr5g04070a* | Cytochrome P450 71B28 | Secondary metabolism | 1.28 |
| *VDAG_JR2_Chr5g05090a* | Putative uncharacterized protein | Secondary metabolism | 1.32 |
| *VDAG_JR2_Chr5g05200a* | Drug transporter | Secondary metabolism | -1.06 |
| *VDAG_JR2_Chr5g05220a* | Putative uncharacterized protein | Secondary metabolism | -1.26 |
| *VDAG_JR2_Chr5g08040a* | Phenylalanine ammonia-lyase | Secondary metabolism | 1.08 |
| *VDAG_JR2_Chr5g10110a* | MFS gliotoxin efflux transporter GliA | Secondary metabolism | 1.84 |
| *VDAG_JR2_Chr5g10770a* | 1-aminocyclopropane-1-carboxylate synthase | Secondary metabolism | 1.01 |
| *VDAG_JR2_Chr5g10870a* | Benzoate 4-monooxygenase cytochrome P450 | Secondary metabolism | 1.76 |
| *VDAG_JR2_Chr5g11050a* | Cellulose-binding protein | Secondary metabolism | 1.20 |
| *VDAG_JR2_Chr5g11650a* | D-xylose-proton symporter | Secondary metabolism | 1.07 |
| *VDAG_JR2_Chr5g11730a* | MFS transporter | Secondary metabolism | -1.47 |
| *VDAG_JR2_Chr5g11790a* | Putative uncharacterized protein | Secondary metabolism | 1.42 |
| *VDAG_JR2_Chr6g01130a* | FAD binding domain-containing protein | Secondary metabolism | -2.25 |
| *VDAG_JR2_Chr6g02300a* | Choline dehydrogenase | Secondary metabolism | 1.11 |
| *VDAG_JR2_Chr6g02980a* | Acetylcholinesterase | Secondary metabolism | 1.44 |
| *VDAG_JR2_Chr6g04570a* | Het-eN | Secondary metabolism | 1.03 |
| *VDAG_JR2_Chr6g05120a* | Putative uncharacterized protein (Elv1) | Secondary metabolism | -3.68 |
| *VDAG_JR2_Chr6g05160a* | Putative uncharacterized protein | Secondary metabolism | -1.17 |
| *VDAG_JR2_Chr6g06060a* | 4-trimethylaminobutyraldehyde dehydrogenase | Secondary metabolism | 1.71 |
| *VDAG_JR2_Chr6g08420a* | Amine oxidase B | Secondary metabolism | 1.00 |
| *VDAG_JR2_Chr6g08530a* | Ent-kaurene oxidase | Secondary metabolism | 1.39 |
| *VDAG_JR2_Chr6g08650a* | Phenylacetone monooxygenase | Secondary metabolism | 1.52 |
| *VDAG_JR2_Chr6g08940a* | Cytochrome P450 | Secondary metabolism | 1.02 |
| *VDAG_JR2_Chr6g09100a* | Putative uncharacterized protein | Secondary metabolism | -1.00 |
| *VDAG_JR2_Chr6g10570a* | MFS transporter | Secondary metabolism | 2.82 |
| *VDAG_JR2_Chr6g10890a* | Putative uncharacterized protein | Secondary metabolism | -1.54 |
| *VDAG_JR2_Chr7g02120a* | Methyltransferase | Secondary metabolism | 1.04 |
| *VDAG_JR2_Chr7g03200a* | Putative uncharacterized protein | Secondary metabolism | -1.81 |
| *VDAG_JR2_Chr7g03400a* | Phthalate transporter | Secondary metabolism | 1.11 |
| *VDAG_JR2_Chr7g08670a* | Putative uncharacterized protein | Secondary metabolism | 1.03 |
| *VDAG_JR2_Chr7g10470a* | Putative uncharacterized protein (Fragment) | Secondary metabolism | 1.27 |
| *VDAG_JR2_Chr8g00310a* | LolT-1 | Secondary metabolism | 1.39 |
| *VDAG_JR2_Chr8g01170a* | D-alanine-poly(Phosphoribitol) ligase subunit 1 | Secondary metabolism | 1.55 |
| *VDAG_JR2_Chr8g01650a* | 3-carboxy-cis,cis-muconate cycloisomerase | Secondary metabolism | 1.27 |
| *VDAG_JR2_Chr8g01660a* | Putative uncharacterized protein | Secondary metabolism | 1.23 |
| *VDAG_JR2_Chr8g02070a* | Flavin-containing monooxygenase (EC 1.-.-.-) | Secondary metabolism | -1.98 |
| *VDAG_JR2_Chr8g02280a* | Vitamin H transporter 1 | Secondary metabolism | -1.57 |
| *VDAG_JR2_Chr8g02550a* | SGE1 protein | Secondary metabolism | -1.29 |
| *VDAG_JR2_Chr8g04330a* | Choline transport protein | Secondary metabolism | 1.02 |
| *VDAG_JR2_Chr8g07520a* | NADP:D-xylose dehydrogenase | Secondary metabolism | -1.62 |
| *VDAG_JR2_Chr8g09050a* | Averantin oxidoreductase | Secondary metabolism | -1.14 |
| *VDAG_JR2_Chr8g10320a* | Putative uncharacterized protein | Secondary metabolism | -2.71 |
| *VDAG_JR2_Chr8g10540a* | Aflatoxin biosynthesis ketoreductase nor-1 | Secondary metabolism | -2.08 |
| *VDAG_JR2_Chr8g11120a* | Integral membrane protein | Secondary metabolism | -1.59 |
| *VDAG_JR2_Chr1g04240a* | CHIP6 protein | Virulence, disease factors | 1.55 |
| *VDAG_JR2_Chr1g11720a* | Putative uncharacterized protein | Virulence, disease factors | 1.09 |
| *VDAG_JR2_Chr1g12490a* | Putative uncharacterized protein | Virulence, disease factors | 1.02 |
| *VDAG_JR2_Chr1g22650a* | D-arabinono-1,4-lactone oxidase | Virulence, disease factors | -1.07 |
| *VDAG_JR2_Chr2g02970a* | Integral membrane protein | Virulence, disease factors | -1.16 |
| *VDAG_JR2_Chr2g02980a* | Feruloyl esterase B | Virulence, disease factors | -2.75 |
| *VDAG_JR2_Chr2g05500a* | CFEM domain-containing protein | Virulence, disease factors | 1.99 |
| *VDAG_JR2_Chr2g08480a* | Putative uncharacterized protein | Virulence, disease factors | 1.42 |
| *VDAG_JR2_Chr3g00980a* | Aflatoxin biosynthesis ketoreductase nor-1 | Virulence, disease factors | -1.10 |
| *VDAG_JR2_Chr3g11660a* | Integral membrane protein | Virulence, disease factors | -1.20 |
| *VDAG_JR2_Chr4g07970a* | Feruloyl esterase B | Virulence, disease factors | 1.64 |
| *VDAG_JR2_Chr4g09730a* | Putative uncharacterized protein | Virulence, disease factors | 1.98 |
| *VDAG_JR2_Chr4g11930a* | Integral membrane protein | Virulence, disease factors | -1.44 |
| *VDAG_JR2_Chr5g03450a* | Putative uncharacterized protein | Virulence, disease factors | -1.56 |
| *VDAG_JR2_Chr5g03870a* | Putative uncharacterized protein | Virulence, disease factors | -1.08 |
| *VDAG_JR2_Chr5g07050a* | CHIP6 protein | Virulence, disease factors | -1.15 |
| *VDAG_JR2_Chr5g07850a* | Candidapepsin-3 | Virulence, disease factors | 1.60 |
| *VDAG_JR2_Chr5g10780a* | Cyanide hydratase | Virulence, disease factors | 1.15 |
| *VDAG_JR2_Chr7g01210a* | Acetylxylan esterase | Virulence, disease factors | -1.27 |
| *VDAG_JR2_Chr7g02720a* | Integral membrane protein | Virulence, disease factors | -2.64 |
| *VDAG_JR2_Chr8g00830a* | Putative uncharacterized protein | Virulence, disease factors | -1.81 |
| *VDAG_JR2_Chr8g01660a* | Putative uncharacterized protein | Virulence, disease factors | 1.23 |
| *VDAG_JR2_Chr8g05600a* | Integral membrane protein | Virulence, disease factors | -2.36 |
| *VDAG_JR2_Chr8g10310a* | Fatty acid synthase S-acetyltransferase | Virulence, disease factors | -3.29 |
| *VDAG_JR2_Chr8g10540a* | Aflatoxin biosynthesis ketoreductase nor-1 | Virulence, disease factors | -2.08 |
| *VDAG_JR2_Chr8g10640a* | Integral membrane protein | Virulence, disease factors | -1.86 |
| *VDAG_JR2_Chr8g11120a* | Integral membrane protein | Virulence, disease factors | -1.59 |

Candidates mentioned in the article are highlighted in yellow.
