# Supplementary material for: Verticillium dahliae Vta3 promotes ELV1 virulence factor gene expression in xylem sap, but tames Mtf1-mediated late stages of fungus-plant interactions and microsclerotia formation
Source: PLoS Pathog. 2023 Jan 30;19(1):e1011100. doi: 10.1371/journal.ppat.1011100 (PMC9910802; doi:10.1371/journal.ppat.1011100)
Supplement: S5 Table — (DOCX) [file ppat.1011100.s018.docx]

**S5 Table. List of *Verticillium dahliae* genes induced in their transcription dependent on Vta3 with a log_2_(fold change) ≤ -1 found in significantly enriched categories by FunCat analysis.**

| **Gene identifier** | **Protein name** | **FunCat description** | **log_2_(fold change)** |
| --- | --- | --- | --- |
| *VDAG_JR2_Chr2g02970a* | Integral membrane protein | Disease, virulence and defense | -1.16 |
| *VDAG_JR2_Chr2g07000a* | SnodProt1 (Cp2) | Disease, virulence and defense | -1.36 |
| *VDAG_JR2_Chr2g11980a* | Sulfate adenylyltransferase (EC 2.7.7.4) (ATP-sulfurylase) (Sulfate adenylate transferase) | Disease, virulence and defense | -2.05 |
| *VDAG_JR2_Chr3g09940a* | Endo-1,3(4)-beta-glucanase | Disease, virulence and defense | -3.96 |
| *VDAG_JR2_Chr3g11660a* | Integral membrane protein | Disease, virulence and defense | -1.20 |
| *VDAG_JR2_Chr4g05930a* | Putative uncharacterized protein | Disease, virulence and defense | -1.09 |
| *VDAG_JR2_Chr4g11930a* | Integral membrane protein | Disease, virulence and defense | -1.44 |
| *VDAG_JR2_Chr5g03450a* | Putative uncharacterized protein | Disease, virulence and defense | -1.56 |
| *VDAG_JR2_Chr5g03870a* | Putative uncharacterized protein | Disease, virulence and defense | -1.08 |
| *VDAG_JR2_Chr6g10960a* | Indoleamine 2,3-dioxygenase family protein | Disease, virulence and defense | -1.14 |
| *VDAG_JR2_Chr7g00860a* | SnodProt1 (Cp1) | Disease, virulence and defense | -1.45 |
| *VDAG_JR2_Chr7g02720a* | Integral membrane protein | Disease, virulence and defense | -2.64 |
| *VDAG_JR2_Chr8g05600a* | Integral membrane protein | Disease, virulence and defense | -2.36 |
| *VDAG_JR2_Chr8g10640a* | Integral membrane protein | Disease, virulence and defense | -1.86 |
| *VDAG_JR2_Chr8g11120a* | Integral membrane protein | Disease, virulence and defense | -1.59 |

Candidates mentioned in the article are highlighted in yellow.
