# Supplementary material for: Verticillium dahliae Vta3 promotes ELV1 virulence factor gene expression in xylem sap, but tames Mtf1-mediated late stages of fungus-plant interactions and microsclerotia formation
Source: PLoS Pathog. 2023 Jan 30;19(1):e1011100. doi: 10.1371/journal.ppat.1011100 (PMC9910802; doi:10.1371/journal.ppat.1011100)
Supplement: S6 Table — (DOCX) [file ppat.1011100.s019.docx]

**S6 Table. List of *Verticillium dahliae* genes reduced in their transcription dependent on Vta3 with a log_2_(fold change) ≥ 1 found in significantly enriched categories by FunCat analysis.**

| **Gene identifier** | **Protein name** | **FunCat description** | **log_2_(fold change)** |
| --- | --- | --- | --- |
| *VDAG_JR2_Chr1g00540a* | DNA repair protein rhp41 | DNA damage response | 1.22 |
| *VDAG_JR2_Chr1g03380a* | DNA-repair protein rad13 | DNA damage response | 1.29 |
| *VDAG_JR2_Chr1g06270a* | DNA repair protein Rad7 | DNA damage response | 1.11 |
| *VDAG_JR2_Chr1g06280a* | DNA repair protein RAD16 | DNA damage response | 1.29 |
| *VDAG_JR2_Chr2g06600a* | Protein kinase rad3 | DNA damage response | 1.95 |
| *VDAG_JR2_Chr2g11640a* | Sister chromatid cohesion protein Eso1 | DNA damage response | 1.10 |
| *VDAG_JR2_Chr3g00150a* | Putative uncharacterized protein | DNA damage response | 1.03 |
| *VDAG_JR2_Chr3g00210a* | Cullin-3 | DNA damage response | 1.08 |
| *VDAG_JR2_Chr3g07260a* | DNA repair protein RAD51 | DNA damage response | 1.50 |
| *VDAG_JR2_Chr3g07390a* | DNA mismatch repair protein mutL | DNA damage response | 1.42 |
| *VDAG_JR2_Chr3g12420a* | Ankyrin repeat and SOCS box protein | DNA damage response | 1.40 |
| *VDAG_JR2_Chr4g09750a* | Fibronectin type 3 and ankyrin repeat domains protein | DNA damage response | 1.52 |
| *VDAG_JR2_Chr5g05650a* | DNA repair helicase RAD3 | DNA damage response | 1.02 |
| *VDAG_JR2_Chr5g09240a* | DNA ligase (EC 6.5.1.1) | DNA damage response | 1.28 |
| *VDAG_JR2_Chr7g02680a* | DNA polymerase kappa | DNA damage response | 1.69 |
| *VDAG_JR2_Chr8g04050a* | Ku70 | DNA damage response | 1.31 |
| *VDAG_JR2_Chr8g06530a* | Helicase C-terminal domain-containing protein | DNA damage response | 1.09 |
| *VDAG_JR2_Chr1g06280a* | DNA repair protein RAD16 | DNA recombination | 1.29 |
| *VDAG_JR2_Chr1g09730a* | Pre-mRNA-splicing factor ATP-dependent RNA helicase PRP43 | DNA recombination | 1.00 |
| *VDAG_JR2_Chr1g15680a* | DEAD/DEAH box helicase | DNA recombination | 2.61 |
| *VDAG_JR2_Chr3g00150a* | Putative uncharacterized protein | DNA recombination | 1.03 |
| *VDAG_JR2_Chr3g03620a* | Polymerase | DNA recombination | 1.79 |
| *VDAG_JR2_Chr3g07260a* | DNA repair protein RAD51 | DNA recombination | 1.50 |
| *VDAG_JR2_Chr4g07930a* | Activating signal cointegrator 1 complex subunit 3 | DNA recombination | 1.14 |
| *VDAG_JR2_Chr5g02960a* | ATP-dependent DNA helicase RecQ | DNA recombination | 1.83 |
| *VDAG_JR2_Chr5g05650a* | DNA repair helicase RAD3 | DNA recombination | 1.02 |
| *VDAG_JR2_Chr5g09240a* | DNA ligase (EC 6.5.1.1) | DNA recombination | 1.28 |
| *VDAG_JR2_Chr6g06220a* | DNA ligase | DNA recombination | 1.83 |
| *VDAG_JR2_Chr7g02680a* | DNA polymerase kappa | DNA recombination | 1.69 |
| *VDAG_JR2_Chr8g06530a* | Helicase C-terminal domain-containing protein | DNA recombination | 1.09 |
| *VDAG_JR2_Chr1g00540a* | DNA repair protein rhp41 | DNA repair | 1.22 |
| *VDAG_JR2_Chr1g03380a* | DNA-repair protein rad13 | DNA repair | 1.29 |
| *VDAG_JR2_Chr1g06270a* | DNA repair protein Rad7 | DNA repair | 1.11 |
| *VDAG_JR2_Chr1g06280a* | DNA repair protein RAD16 | DNA repair | 1.29 |
| *VDAG_JR2_Chr1g09730a* | Pre-mRNA-splicing factor ATP-dependent RNA helicase PRP43 | DNA repair | 1.00 |
| *VDAG_JR2_Chr1g15520a* | DNA excision repair protein ERCC-6 | DNA repair | 1.23 |
| *VDAG_JR2_Chr1g15680a* | DEAD/DEAH box helicase | DNA repair | 2.61 |
| *VDAG_JR2_Chr2g06600a* | Protein kinase rad3 | DNA repair | 1.95 |
| *VDAG_JR2_Chr2g11640a* | Sister chromatid cohesion protein Eso1 | DNA repair | 1.10 |
| *VDAG_JR2_Chr3g00150a* | Putative uncharacterized protein | DNA repair | 1.03 |
| *VDAG_JR2_Chr3g00210a* | Cullin-3 | DNA repair | 1.08 |
| *VDAG_JR2_Chr3g01570a* | Ubiquitin-conjugating enzyme | DNA repair | 1.07 |
| *VDAG_JR2_Chr3g03620a* | Polymerase | DNA repair | 1.79 |
| *VDAG_JR2_Chr3g07260a* | DNA repair protein RAD51 | DNA repair | 1.50 |
| *VDAG_JR2_Chr3g07390a* | DNA mismatch repair protein mutL | DNA repair | 1.42 |
| *VDAG_JR2_Chr3g10400a* | Formamidopyrimidine-DNA glycosylase | DNA repair | 1.71 |
| *VDAG_JR2_Chr3g12420a* | Ankyrin repeat and SOCS box protein | DNA repair | 1.40 |
| *VDAG_JR2_Chr4g06310a* | RING-2 protein | DNA repair | 1.89 |
| *VDAG_JR2_Chr4g07930a* | Activating signal cointegrator 1 complex subunit 3 | DNA repair | 1.14 |
| *VDAG_JR2_Chr4g11330a* | UV-damage endonuclease | DNA repair | 1.96 |
| *VDAG_JR2_Chr5g02960a* | ATP-dependent DNA helicase RecQ | DNA repair | 1.83 |
| *VDAG_JR2_Chr5g05650a* | DNA repair helicase RAD3 | DNA repair | 1.02 |
| *VDAG_JR2_Chr5g06450a* | DNA repair and recombination protein RAD26 | DNA repair | 1.85 |
| *VDAG_JR2_Chr5g09240a* | DNA ligase (EC 6.5.1.1) | DNA repair | 1.28 |
| *VDAG_JR2_Chr6g03590a* | DNA repair helicase RAD25 | DNA repair | 1.73 |
| *VDAG_JR2_Chr6g06220a* | DNA ligase | DNA repair | 1.83 |
| *VDAG_JR2_Chr7g02680a* | DNA polymerase kappa | DNA repair | 1.69 |
| *VDAG_JR2_Chr8g04050a* | Ku70 | DNA repair | 1.31 |
| *VDAG_JR2_Chr8g06530a* | Helicase C-terminal domain-containing protein | DNA repair | 1.09 |
| *VDAG_JR2_Chr8g07040a* | Crossover junction endonuclease MUS81 | DNA repair | 1.26 |
| *VDAG_JR2_Chr1g01670a* | Cytosine-specific methyltransferase (EC 2.1.1.37) | DNA topology | 1.49 |
| *VDAG_JR2_Chr1g06280a* | DNA repair protein RAD16 | DNA topology | 1.29 |
| *VDAG_JR2_Chr1g09730a* | Pre-mRNA-splicing factor ATP-dependent RNA helicase PRP43 | DNA topology | 1.00 |
| *VDAG_JR2_Chr1g15680a* | DEAD/DEAH box helicase | DNA topology | 2.61 |
| *VDAG_JR2_Chr1g17940a* | Hrq1p | DNA topology | 1.33 |
| *VDAG_JR2_Chr2g11040a* | Putative uncharacterized protein | DNA topology | 1.26 |
| *VDAG_JR2_Chr3g03620a* | Polymerase | DNA topology | 1.79 |
| *VDAG_JR2_Chr4g07930a* | Activating signal cointegrator 1 complex subunit 3 | DNA topology | 1.14 |
| *VDAG_JR2_Chr5g02960a* | ATP-dependent DNA helicase RecQ | DNA topology | 1.83 |
| *VDAG_JR2_Chr5g05650a* | DNA repair helicase RAD3 | DNA topology | 1.02 |
| *VDAG_JR2_Chr6g03590a* | DNA repair helicase RAD25 | DNA topology | 1.73 |
| *VDAG_JR2_Chr8g06530a* | Helicase C-terminal domain-containing protein | DNA topology | 1.09 |
| *VDAG_JR2_Chr1g04280a* | NAD(P)H-dependent D-xylose reductase | Secondary metabolism | 1.49 |
| *VDAG_JR2_Chr1g05220a* | Putative uncharacterized protein | Secondary metabolism | 1.50 |
| *VDAG_JR2_Chr1g10990a* | Putative uncharacterized protein | Secondary metabolism | 1.03 |
| *VDAG_JR2_Chr1g17090a* | Putative uncharacterized protein | Secondary metabolism | 1.27 |
| *VDAG_JR2_Chr1g17200a* | Conidial yellow pigment biosynthesis polyketide synthase | Secondary metabolism | 1.44 |
| *VDAG_JR2_Chr1g22830a* | Allantoate permease | Secondary metabolism | 1.27 |
| *VDAG_JR2_Chr1g23310a* | Putative uncharacterized protein | Secondary metabolism | 1.00 |
| *VDAG_JR2_Chr1g23320a* | 4-nitrophenylphosphatase | Secondary metabolism | 1.00 |
| *VDAG_JR2_Chr1g25130a* | Glyoxylate reductase | Secondary metabolism | 1.06 |
| *VDAG_JR2_Chr1g25460a* | Aflatoxin B1 aldehyde reductase member 3 | Secondary metabolism | 2.85 |
| *VDAG_JR2_Chr1g26700a* | 2-ketogluconate reductase | Secondary metabolism | 1.06 |
| *VDAG_JR2_Chr1g26890a* | Pleiotropic ABC multiple drug efflux transporter | Secondary metabolism | 1.11 |
| *VDAG_JR2_Chr1g29150a* | Glyoxalase/bleomycin resistance protein/dioxygenase | Secondary metabolism | 1.09 |
| *VDAG_JR2_Chr2g00060a* | TRI15 protein | Secondary metabolism | 1.09 |
| *VDAG_JR2_Chr2g03090a* | ATP-dependent permease MDL1 | Secondary metabolism | 1.06 |
| *VDAG_JR2_Chr2g03180a* | Epoxide hydrolase | Secondary metabolism | 2.04 |
| *VDAG_JR2_Chr2g04160a* | 4-coumarate-CoA ligase | Secondary metabolism | 1.07 |
| *VDAG_JR2_Chr2g06040a* | Putative uncharacterized protein | Secondary metabolism | 1.47 |
| *VDAG_JR2_Chr2g08250a* | O-acetylhomoserine (Thiol)-lyase | Secondary metabolism | 1.69 |
| *VDAG_JR2_Chr2g08790a* | Ferulic acid esterase A | Secondary metabolism | 1.21 |
| *VDAG_JR2_Chr3g08570a* | Ankyrin repeat protein | Secondary metabolism | 1.35 |
| *VDAG_JR2_Chr4g02070a* | Polyamine transporter 1 | Secondary metabolism | 1.56 |
| *VDAG_JR2_Chr4g03020a* | Bifunctional P-450:NADPH-P450 reductase | Secondary metabolism | 1.62 |
| *VDAG_JR2_Chr4g03530a* | Siderophore iron transporter mirB | Secondary metabolism | 2.01 |
| *VDAG_JR2_Chr4g03830a* | Putative uncharacterized protein | Secondary metabolism | 4.00 |
| *VDAG_JR2_Chr4g04680a* | L-aminoadipate-semialdehyde dehydrogenase large subunit | Secondary metabolism | 1.09 |
| *VDAG_JR2_Chr4g06340a* | Putative uncharacterized protein | Secondary metabolism | 1.14 |
| *VDAG_JR2_Chr4g06660a* | Fumarylacetoacetate hydrolase domain-containing protein | Secondary metabolism | 1.25 |
| *VDAG_JR2_Chr4g09680a* | Putative uncharacterized protein | Secondary metabolism | 1.04 |
| *VDAG_JR2_Chr4g11360a* | Quinate permease | Secondary metabolism | 1.35 |
| *VDAG_JR2_Chr5g00970a* | Choline monooxygenase | Secondary metabolism | 1.26 |
| *VDAG_JR2_Chr5g01070a* | Multidrug resistance protein | Secondary metabolism | 2.46 |
| *VDAG_JR2_Chr5g01440a* | FAD binding domain-containing protein | Secondary metabolism | 1.58 |
| *VDAG_JR2_Chr5g02800a* | Putative uncharacterized protein | Secondary metabolism | 1.83 |
| *VDAG_JR2_Chr5g04070a* | Cytochrome P450 71B28 | Secondary metabolism | 1.28 |
| *VDAG_JR2_Chr5g05090a* | Putative uncharacterized protein | Secondary metabolism | 1.32 |
| *VDAG_JR2_Chr5g08040a* | Phenylalanine ammonia-lyase | Secondary metabolism | 1.08 |
| *VDAG_JR2_Chr5g10110a* | MFS gliotoxin efflux transporter GliA | Secondary metabolism | 1.84 |
| *VDAG_JR2_Chr5g10770a* | 1-aminocyclopropane-1-carboxylate synthase | Secondary metabolism | 1.01 |
| *VDAG_JR2_Chr5g10870a* | Benzoate 4-monooxygenase cytochrome P450 | Secondary metabolism | 1.76 |
| *VDAG_JR2_Chr5g11050a* | Cellulose-binding protein | Secondary metabolism | 1.20 |
| *VDAG_JR2_Chr5g11650a* | D-xylose-proton symporter | Secondary metabolism | 1.07 |
| *VDAG_JR2_Chr5g11790a* | Putative uncharacterized protein | Secondary metabolism | 1.42 |
| *VDAG_JR2_Chr6g02300a* | Choline dehydrogenase | Secondary metabolism | 1.11 |
| *VDAG_JR2_Chr6g02980a* | Acetylcholinesterase | Secondary metabolism | 1.44 |
| *VDAG_JR2_Chr6g04570a* | Het-eN | Secondary metabolism | 1.03 |
| *VDAG_JR2_Chr6g06060a* | 4-trimethylaminobutyraldehyde dehydrogenase | Secondary metabolism | 1.71 |
| *VDAG_JR2_Chr6g08420a* | Amine oxidase B | Secondary metabolism | 1.00 |
| *VDAG_JR2_Chr6g08530a* | Ent-kaurene oxidase | Secondary metabolism | 1.39 |
| *VDAG_JR2_Chr6g08650a* | Phenylacetone monooxygenase | Secondary metabolism | 1.52 |
| *VDAG_JR2_Chr6g08940a* | Cytochrome P450 | Secondary metabolism | 1.02 |
| *VDAG_JR2_Chr6g10570a* | MFS transporter | Secondary metabolism | 2.82 |
| *VDAG_JR2_Chr7g02120a* | Methyltransferase | Secondary metabolism | 1.04 |
| *VDAG_JR2_Chr7g03400a* | Phthalate transporter | Secondary metabolism | 1.11 |
| *VDAG_JR2_Chr7g08670a* | Putative uncharacterized protein | Secondary metabolism | 1.03 |
| *VDAG_JR2_Chr7g10470a* | Putative uncharacterized protein (Fragment) | Secondary metabolism | 1.27 |
| *VDAG_JR2_Chr8g00310a* | LolT-1 | Secondary metabolism | 1.39 |
| *VDAG_JR2_Chr8g01170a* | D-alanine-poly(Phosphoribitol) ligase subunit 1 | Secondary metabolism | 1.55 |
| *VDAG_JR2_Chr8g01650a* | 3-carboxy-cis,cis-muconate cycloisomerase | Secondary metabolism | 1.27 |
| *VDAG_JR2_Chr8g01660a* | Putative uncharacterized protein | Secondary metabolism | 1.23 |
|  |  |  |  |
| *VDAG_JR2_Chr8g04330a* | Choline transport protein | Secondary metabolism | 1.02 |
