# Supplementary material for: Verticillium dahliae Vta3 promotes ELV1 virulence factor gene expression in xylem sap, but tames Mtf1-mediated late stages of fungus-plant interactions and microsclerotia formation
Source: PLoS Pathog. 2023 Jan 30;19(1):e1011100. doi: 10.1371/journal.ppat.1011100 (PMC9910802; doi:10.1371/journal.ppat.1011100)
Supplement: S7 Table — (DOCX) [file ppat.1011100.s020.docx]

**S7 Table. List of *Verticillium dahliae* genes induced in their transcription dependent on Vta3 with domains of derived proteins and their putative function.**

| **Gene identifier** | **Putative functional category** | **Domains** | **log_2_(fold change)** |
| --- | --- | --- | --- |
| **Cell wall/cell surface (5 candidates)** | | | |
| *VDAG_JR2_Chr1g03390a* | Cell wall/cell surface | Cell wall protein YJL171C/Tos1, C-terminal (PF10287, IPR018805), Cell wall protein YJL171C/Tos1, N-terminal (PF10290, IPR018807) | -2.16 |
| *VDAG_JR2_Chr1g10580a* | Cell wall/cell surface (GPI anchored protein) | - | -3.11 |
| *VDAG_JR2_Chr1g28810a* | Cell wall/cell surface | Cell wall mannoprotein 1 (PF12296, IPR021054) | -4.01 |
| *VDAG_JR2_Chr2g04040a* | Cell wall/cell surface | Agglutinin-like protein repeat (PF05792, IPR008440) | -2.57 |
| VDAG_JR2_Chr6g01720a | Cell wall/cell surface | Extracellular membrane protein, CFEM domain (PF05730, IPR008427) | -2.17 |
| **Metabolism (13 candidates)** | | | |
| *VDAG_JR2_Chr2g02980a* | Metabolism | Tannase/feruloyl esterase (PF07519, IPR011118) | -2.75 |
| *VDAG_JR2_Chr2g04120a* | Metabolism (carbohydrate) | Glycosyltransferase 2-like (PF00535, IPR001173) | -2.16 |
| *VDAG_JR2_Chr2g04540a* | Metabolism (carbohydrate) | Glycosyl hydrolase family 26 domain (PF02156, IPR022790) | -2.11 |
| *VDAG_JR2_Chr2g11980a* | Metabolism (sulfur amino acid, connection to virulence according to FungCat analysis) | ATP-sulfurylase PUA-like domain (PF14306, IPR025980), Sulphate adenylyltransferase catalytic domain (PF01747, IPR024951), Adenylyl-sulphate kinase (PF01583) | -2.05 |
| *VDAG_JR2_Chr3g02150a* | Metabolism (chitin) | Glycoside hydrolase family 18, catalytic domain (PF00704, IPR001223) | -2.65 |
| *VDAG_JR2_Chr3g02970a* | Metabolism (carbohydrate) | Glycoside hydrolase family 16 (PF00722, IPR000757) | -2.89 |
| *VDAG_JR2_Chr3g06450a* | Metabolism (chitin) | Glycoside hydrolase family 18, catalytic domain (PF00704, IPR001223) | -2.59 |
| *VDAG_JR2_Chr3g09940a* | Metabolism (carbohydrate) | Glycosyl hydrolase family 81, C-terminal domain (PF17652, IPR040720), Glycosyl hydrolase family 81, N-terminal (PF03639, IPR040451) | -3.71 |
| *VDAG_JR2_Chr3g12270a* | Metabolism (chitin) | Chitin synthase (PF03142) | -2.65 |
| *VDAG_JR2_Chr4g08080a* | Metabolism (carbohydrate) | Carbohydrate-binding WSC (PF01822, IPR002889), Glycoside hydrolase family 16 (PF00722, IPR000757) | -2.14 |
| *VDAG_JR2_Chr6g09410a* | Metabolism (carbohydrate) | Glycoside hydrolase, family 5 (PF00150, IPR001547) | -2.14 |
| *VDAG_JR2_Chr8g05670a* | Metabolism (lipid) | Patatin-like phospholipase domain (PF01734, IPR002641) | -2.63 |
| *VDAG_JR2_Chr8g10320a* | Metabolism (sulfur) | Serine hydrolase FSH (PF03959, IPR005645) | -2.56 |
| **Others (2 candidates)** | | | |
| *VDAG_JR2_Chr1g10860a* | Others (phosphorylation) | Protein kinase domain (PF00069, IPR000719) | -2.17 |
| *VDAG_JR2_Chr5g08500a* | Others (GPI anchor synthesis) | GPI mannosyltransferase (PF03901, IPR005599) | -2.04 |
| **Proteolysis (4 candidates)** | | | |
| *VDAG_JR2_Chr1g23740a* | Proteolysis | F-box domain (PF12937, IPR001810) | -2.01 |
| *VDAG_JR2_Chr4g11990a* | Proteolysis | F-box domain (PF12937, IPR001810), Leucine-rich repeat (PF13516, IPR001611) | -2.03 |
| *VDAG_JR2_Chr5g09980a* | Proteolysis | Serine proteases, trypsin domain (PF00089, IPR001254) | -2.47 |
| *VDAG_JR2_Chr6g06110a* | Proteolysis | Alpha/beta hydrolase fold-1 (PF00561, IPR000073), Peptidase S33 tripeptidyl aminopeptidase-like, C-terminal (PF08386, IPR013595) | -3.55 |
| **Redox process (13 candidates)** | | | |
| *VDAG_JR2_Chr1g08040a* | Redox process | PPPDE putative peptidase domain (PF05903, IPR008580), Thioredoxin domain (PF00085, IPR013766), PUL domain (PF08324, IPR013535) | -2.05 |
| *VDAG_JR2_Chr1g24570a* | Redox process | Luciferase-like domain (PF00296, IPR011251) | -2.32 |
| *VDAG_JR2_Chr1g24600a* | Redox process | Flavin monooxygenase-like (PF00743, IPR020946) | -2.90 |
| *VDAG_JR2_Chr2g00570a* | Redox process | Acyl-CoA dehydrogenase/oxidase, N-terminal (PF02771, IPR013786), Acyl-CoA dehydrogenase, C-terminal domain (PF08028, IPR013107) | -3.35 |
| *VDAG_JR2_Chr3g06380a* | Redox process | FAD-binding domain (PF01494, IPR002938) | -2.11 |
| *VDAG_JR2_Chr3g12280a* | Redox process (carbohydrate metabolism) | UDP-glucose/GDP-mannose dehydrogenase, N-terminal (PF03721, IPR001732), UDP-glucose/GDP-mannose dehydrogenase, dimerization (PF00984, IPR014026) | -4.62 |
| *VDAG_JR2_Chr4g04660a* | Redox process | FMN-dependent dehydrogenase (PF01070, IPR000262) | -2.03 |
| *VDAG_JR2_Chr4g10620a* | Redox process | FAD-binding domain (PF01494, IPR002938) | -2.66 |
| *VDAG_JR2_Chr6g01130a* | Redox process | FAD-binding domain (PF01494, IPR002938), Acetoacetate decarboxylase (PF06314, IPR010451) | -2.11 |
| *VDAG_JR2_Chr7g02710a* | Redox process | Cytochrome P450 (PF00067, IPR001128) | -2.50 |
| *VDAG_JR2_Chr8g00900a* | Redox process | NAD(P)-binding Rossmann-like domain (PF13450) | -2.11 |
| *VDAG_JR2_Chr8g07330a* | Redox process (stress response & detoxification) | Alternative oxidase (PF01786, IPR002680) | -2.42 |
| *VDAG_JR2_Chr8g10540a* | Redox process | Short-chain dehydrogenase/reductase SDR (PF00106, IPR002347) | -2.08 |
| **Secondary metabolism (2 candidates)** | | | |
| *VDAG_JR2_Chr2g10010a/*  *AYG1* | Secondary metabolism | Esterase FrsA-like (PF06500, IPR010520) | -2.12 |
| *VDAG_JR2_Chr8g10310a* | Secondary metabolism | Beta-ketoacyl synthase, N-terminal (PF00109, IPR014030), Beta-ketoacyl synthase, C-terminal (PF02801, IPR014031), Acyl transferase (PF00698, IPR014043), Polyketide synthase, dehydratase domain (PF14765, IPR020807), Alcohol dehydrogenase, N-terminal (PF08240, IPR013154), Alcohol dehydrogenase, C-terminal (PF00107, IPR013149), Polyketide synthase, ketoreductase domain (PF08659, IPR013968), Phosphopantetheine binding ACP domain (PF00550, IPR009081) | -3.01 |
| **Stress response & detoxification (5 candidates)** | | | |
| *VDAG_JR2_Chr1g11540a* | Stress response & detoxification (unfolded protein folding) | DnaJ domain (PF00226, IPR001623), Chaperone DnaJ, C-terminal (PF01556, IPR002939) | -2.15 |
| *VDAG_JR2_Chr1g22750a* | Stress response & detoxification | SNF2-related, N-terminal domain (PF00176, IPR000330), Zinc finger, C3HC4 RING-type (PF00097, IPR018957), Helicase, C-terminal (PF00271, IPR001650) | -3.45 |
| *VDAG_JR2_Chr3g03520a* | Stress response & detoxification (unfolded protein folding, response to heat) | DnaJ domain (PF00226, IPR001623), Chaperone DnaJ, C-terminal (PF01556, IPR002939), Heat shock protein DnaJ, cysteine-rich domain (PF00684, IPR001305) | -2.02 |
| *VDAG_JR2_Chr4g06820a* | Stress response & detoxification | Heat shock protein 70 family (PF00012, IPR013126) | -2.44 |
| *VDAG_JR2_Chr6g08130a* | Stress response & detoxification (unfolded protein folding) | Histidine kinase/HSP90-like ATPase (PF02518, IPR003594), Heat shock protein Hsp90 family (PF00183, IPR001404) | -2.38 |
| **Transcription (5 candidates)** | | | |
| *VDAG_JR2_Chr1g07600a* | Transcription | DNA-binding RFX-type winged-helix domain (PF02257, IPR003150) | -3.02 |
| *VDAG_JR2_Chr4g00280a* | Transcription (sequence similarity) | Regulator of drug sensitivity 2 (PTHR31986) | -2.10 |
| *VDAG_JR2_Chr4g04620a* | Transcription | Fungal transcription factor (PF11951, IPR021858) | -2.32 |
| *VDAG_JR2_Chr5g05720a* | Transcription | Zinc finger C2H2-type (PF00096, IPR013087) | -2.09 |
| *VDAG_JR2_Chr6g06120a* | Transcription | Fungal transcription factor (PF11951, IPR021858) | -2.18 |
| **Transport (6 candidates)** | | | |
| *VDAG_JR2_Chr1g05010a* | Transport (transmembrane) | Proton-dependent oligopeptide transporter family (PF00854, IPR000109) | -2.05 |
| *VDAG_JR2_Chr2g02180a* | Transport (transmembrane) | Major facilitator superfamily (PF07690, IPR011701) | -2.04 |
| *VDAG_JR2_Chr4g05940a* | Transport (transmembrane) | Major facilitator, sugar transporter-like (PF00083, IPR005828) | -2.11 |
| *VDAG_JR2_Chr7g02650a* | Transport (transmembrane) | Acetate transporter GPR1/FUN34/SatP family (PF01184, IPR000791) | -2.03 |
| *VDAG_JR2_Chr8g01840a* | Transport (transmembrane) | Major facilitator, sugar transporter-like (PF00083, IPR005828) | -2.03 |
| *VDAG_JR2_Chr8g09210a* | Transport (transmembrane) | Major facilitator, sugar transporter-like (PF00083, IPR005828) | -2.31 |
| **Unknown function (42 candidates)** | | | |
| *VDAG_JR2_Chr1g00545a* | Unknown function | Zinc finger, AN1-type (PF01428, IPR000058) | -2.30 |
| *VDAG_JR2_Chr1g03520a* | Unknown function (potentially extracellular, but no signal peptide) | - | -2.01 |
| *VDAG_JR2_Chr1g12610a* | Unknown function (potentially extracellular) | Collagen alpha (PTHR24023) | -2.80 |
| *VDAG_JR2_Chr1g18540a* | Unknown function | Viral A-type inclusion protein repeat (PF04508, IPR007596) | -2.17 |
| *VDAG_JR2_Chr2g00650a* | Unknown function | - | -2.29 |
| *VDAG_JR2_Chr2g04030a* | Unknown function | - | -2.56 |
| *VDAG_JR2_Chr2g04050a* | Unknown function (potentially extracellular) | - | -2.61 |
| *VDAG_JR2_Chr2g04760a* | Unknown function | Six-bladed beta-propeller, TolB-like (2.120.10.30, IPR011042) | -2.15 |
| *VDAG_JR2_Chr2g04970a* | Unknown function (potentially extracellular) | - | -3.87 |
| *VDAG_JR2_Chr2g10300a* | Unknown function | - | -3.75 |
| *VDAG_JR2_Chr2g11700a* | Unknown function | - | -2.16 |
| *VDAG_JR2_Chr3g10150a* | Unknown function | Phenazine biosynthesis PhzF protein (PF02567, IPR003719) | -2.31 |
| *VDAG_JR2_Chr3g12290a* | Unknown function | - | -2.60 |
| *VDAG_JR2_Chr4g00900a* | Unknown function | - | -2.24 |
| *VDAG_JR2_Chr4g06480a* | Unknown function | - | -2.20 |
| *VDAG_JR2_Chr4g06600a* | Unknown function (potentially extracellular) | Alpha/beta hydrolase fold-1 (PF12697, IPR000073) | -2.30 |
| *VDAG_JR2_Chr4g11920a* | Unknown function | - | -2.82 |
| *VDAG_JR2_Chr5g00210a* | Unknown function (potential membrane protein) | - | -2.64 |
| *VDAG_JR2_Chr5g01470a* | Unknown function | Protein of unknown function DUF3140 (PF11338, IPR021487), Hypervirulence associated protein, TUDOR domain (PF11160, IPR021331) | -2.20 |
| *VDAG_JR2_Chr5g02010a* | Unknown function | - | -2.65 |
| *VDAG_JR2_Chr5g03120a* | Unknown function (potentially extracellular) | CHRD (PF07452, IPR010895) | -2.22 |
| *VDAG_JR2_Chr5g07170a* | Unknown function | - | -2.12 |
| *VDAG_JR2_Chr5g10560a* | Unknown function | Hemerythrin-like (PF01814, IPR012312) | -2.00 |
| *VDAG_JR2_Chr6g01910a* | Unknown function | - | -2.02 |
| *VDAG_JR2_Chr6g01920a* | Unknown function (potential effector) | - | -2.42 |
| *VDAG_JR2_Chr6g01930a* | Unknown function | SET domain (PF00856, IPR001214) | -2.06 |
| *VDAG_JR2_Chr6g03140a* | Unknown function (potentially extracellular; no signal peptide) | Domain of unknown function DUF1996 (PF09362, IPR018535) | -2.22 |
| *VDAG_JR2_Chr6g03150a* | Unknown function | - | -2.21 |
| *VDAG_JR2_Chr6g03400a* | Unknown function | - | -4.83 |
| *VDAG_JR2_Chr6g05080a* | Unknown function | - | -3.16 |
| *VDAG_JR2_Chr6g05100a* | Unknown function | - | -2.75 |
| *VDAG_JR2_Chr6g09620a* | Unknown function | - | -2.29 |
| *VDAG_JR2_Chr7g01020a* | Unknown function (potential extracellular, small, C-rich protein, no signal peptide) | - | -3.36 |
| *VDAG_JR2_Chr7g01720a* | Unknown function | - | -2.20 |
| *VDAG_JR2_Chr7g02030a* | Unknown function (potential effector) | - | -5.75 |
| *VDAG_JR2_Chr7g02720a* | Unknown function (potential membrane protein, connection to virulence according to FungCat analysis) | PTH11-like integral membrane protein (AFU_ORTHOLOG AFUA_5G11245) (PTHR33048) | -2.54 |
| *VDAG_JR2_Chr7g04080a* | Unknown function | - | -2.05 |
| *VDAG_JR2_Chr8g01850a* | Unknown function | - | -2.85 |
| *VDAG_JR2_Chr8g02710a* | Unknown function (potentially extracellular) | Chitin-binding, type 1 (PF00187, IPR001002) | -3.18 |
| *VDAG_JR2_Chr8g05600a* | Unknown function (potential membrane protein, connection to virulence according to FungCat analysis) | PTH11-like integral membrane protein (AFU_ORTHOLOG AFUA_5G11245) (PTHR33048) | -2.08 |
| *VDAG_JR2_Chr8g09410a* | Unknown function (potentially extracellular) | - | -2.08 |
| *VDAG_JR2_Chr8g10750a* | Unknown function | SMODS and SLOG-associating 2TM effector domain, fungi (PF18142, IPR041622) | -2.04 |
| **Virulence (3 candidates)** | | | |
| *VDAG_JR2_Chr2g04430a* | Virulence | Cutinase/acetylxylan esterase (PF01083, IPR000675) | -2.82 |
| *VDAG_JR2_Chr4g11280a* | Virulence | Pectate lyase PlyH/PlyE-like (PF03211, IPR004898) | -2.07 |
| ***VDAG_JR2_Chr6g05120a/***  ***ELV1*** | Virulence | Egh16-like virulence factor (PF11327, IPR021476) | -3.68 |

Candidates mentioned in the article are highlighted in yellow; bold: candidate investigated in this study.
