# Supplementary material for: Verticillium dahliae Vta3 promotes ELV1 virulence factor gene expression in xylem sap, but tames Mtf1-mediated late stages of fungus-plant interactions and microsclerotia formation
Source: PLoS Pathog. 2023 Jan 30;19(1):e1011100. doi: 10.1371/journal.ppat.1011100 (PMC9910802; doi:10.1371/journal.ppat.1011100)
Supplement: S8 Table — (DOCX) [file ppat.1011100.s021.docx]

**S8 Table. List of *Verticillium dahliae* genes reduced in their transcription dependent on Vta3 with domains of derived proteins and their putative function.**

| **Gene identifier** | **Putative functional category** | **Domains** | **log_2_(fold change)** |
| --- | --- | --- | --- |
| **Cell cycle & signaling (2 candidates)** | | | |
| *VDAG_JR2_Chr5g06830a* | Cell cycle & signaling (kinetochore-associated) | Nuclear MIS12/MIND complex subunit PMF1/Nnf1 (PTHR15459, IPR007128) | 2.15 |
| *VDAG_JR2_Chr8g10150a* | Cell cycle & signaling (chromatin organization) | Sirtuin family (PF02146, IPR003000) | 2.72 |
| **Metabolism (5 candidates)** | | | |
| *VDAG_JR2_Chr2g04570a* | Metabolism (carbohydrate) | Glycoside hydrolase family 16 (PF00722, IPR000757) | 4.14 |
| *VDAG_JR2_Chr5g01710a* | Metabolism (amino acid) | L-asparaginase II (PF06089, IPR010349) | 2.55 |
| *VDAG_JR2_Chr5g03660a* | Metabolism (carbohydrate) | Sugar-phosphate isomerase, RpiB/LacA/LacB family (PF02502, IPR003500) | 2.29 |
| *VDAG_JR2_Chr6g02080a* | Metabolism (riboflavin biosynthesis) | Bacterial bifunctional deaminase-reductase, C-terminal (PF01872, IPR002734) | 2.57 |
| *VDAG_JR2_Chr7g05780a* | Metabolism (carbohydrate) | Glycoside hydrolase family 61 (PF03443, IPR005103) | 2.03 |
| **Others (9 candidates)** | | | |
| *VDAG_JR2_Chr1g01700a* | Others (dephosphorylation) | Alkaline phosphatase (PF00245, IPR001952) | 2.06 |
| *VDAG_JR2_Chr1g02160a* | Others (cell fusion) | Cell fusion protein Dni1/Fig1 (PF12351, IPR033481) | 2.25 |
| *VDAG_JR2_Chr1g04360a* | Others (acetyltransferase) | GNAT domain (PF13302, IPR000182) | 2.04 |
| *VDAG_JR2_Chr1g06420a* | Others (energy) | Mitochondrial chaperone BCS1, N-terminal (PF08740, IPR014851), ATPase, AAA-type, core (PF00004, IPR003959) | 2.31 |
| *VDAG_JR2_Chr2g02370a* | Others (methylation) | PhnB-like (PF06983, IPR028973) | 4.90 |
| *VDAG_JR2_Chr4g01080a* | Others (ATPase) | ATPase, AAA-type, core (PF00004, IPR003959) | 4.17 |
| *VDAG_JR2_Chr4g05530a* | Others (heterokaryon incompatibility) | Heterokaryon incompatibility (PF06985, IPR010730) | 2.13 |
| *VDAG_JR2_Chr4g11240a* | Others (posttranslational modification of tubulin) | Tubulin-tyrosine ligase/Tubulin polyglutamylase (PF03133, IPR004344) | 2.04 |
| *VDAG_JR2_Chr6g05020a* | Others (proteolysis) | Peptidase M54, archaemetzincin (PF07998, IPR012962) | 2.53 |
| **Redox process (3 candidates)** | | | |
| *VDAG_JR2_Chr1g25460a* | Redox process | NADP-dependent oxidoreductase domain (PF00248, IPR023210) | 2.80 |
| *VDAG_JR2_Chr3g11580a* | Redox process | Short-chain dehydrogenase/reductase SDR (PF00106, IPR002347) | 2.16 |
| *VDAG_JR2_Chr8g03350a* | Redox process | Zinc-binding dehydrogenase (PF13602) | 2.24 |
| **RNA metabolism (2 candidates)** | | | |
| *VDAG_JR2_Chr1g02240a* | RNA metabolism | Tetratricopeptide-like repeat (PF18833, IPR040962) | 2.15 |
| *VDAG_JR2_Chr1g15680a* | RNA metabolism | DEAD/DEAH box helicase domain (PF00270, IPR011545), Helicase, C-terminal (PF00271, IPR001650) | 2.61 |
| **Stress response & detoxification (3 candidates)** | | | |
| *VDAG_JR2_Chr1g20940a* | Stress response & detoxification (DNA repair) | Alpha-ketoglutarate-dependent dioxygenase AlkB-like (PF13532, IPR027450), Zinc finger, GRF-type (PF06839, IPR010666) | 2.29 |
| *VDAG_JR2_Chr2g03180a* | Stress response & detoxification | Epoxide hydrolase, N-terminal (PF06441, IPR010497) | 2.04 |
| *VDAG_JR2_Chr8g05360a* | Stress response & detoxification (DNA repair) | VRR-NUC domain (PF08774, IPR014883) | 2.83 |
| **Transcription (2 candidates)** | | | |
| *VDAG_JR2_Chr1g07660a* | Transcription (sequence similarity) | bZIP domain-containing protein (PTHR39607:SF2) | 2.05 |
| ***VDAG_JR2_Chr2g08470a/***  ***MTF1*** | Transcription | Zinc finger C2H2-type (PF00096, IPR013087) | 2.08 |
|  |  |  |  |
| **Transport (5 candidates)** | | | |
| *VDAG_JR2_Chr3g12650a* | Transport (membrane transport vesicles) | Dynamin superfamily (PF00350, IPR022812), Dynamin central domain (PF01031, IPR000375), Dynamin GTPase effector (PF02212, IPR003130) | 2.55 |
| *VDAG_JR2_Chr4g03530a* | Transport (transmembrane) | Major facilitator superfamily (PF07690, IPR011701) | 2.01 |
| *VDAG_JR2_Chr5g01070a* | Transport (transmembrane) | ABC transporter type 1, transmembrane domain (PF00664, IPR011527), ABC transporter-like (PF00005, IPR003439) | 2.12 |
| *VDAG_JR2_Chr5g07830a* | Transport (proton transmembrane) | V-ATPase proteolipid subunit C-like domain (PF00137, IPR002379) | 2.02 |
| *VDAG_JR2_Chr6g10570a* | Transport (transmembrane) | Major facilitator superfamily (PF07690, IPR011701) | 2.71 |
| **Unknown function (31 candidates)** | | | |
| *VDAG_JR2_Chr1g04320a* | Unknown function | BTB/POZ domain (PF00651, IPR000210) | 6.58 |
| *VDAG_JR2_Chr1g06530a* | Unknown function (potential effector) | - | 2.36 |
| *VDAG_JR2_Chr1g25850a* | Unknown function (potential membrane protein) | - | 2.86 |
| *VDAG_JR2_Chr1g25880a* | Unknown function | Forkhead-associated (FHA) domain (PF00498, IPR000253) | 3.13 |
| *VDAG_JR2_Chr1g26450a* | Unknown function (potential effector) | - | 2.08 |
| *VDAG_JR2_Chr1g29710a* | Unknown function (potential cell wall protein) | - | 2.85 |
| *VDAG_JR2_Chr2g05110a* | Unknown function | NmrA-like domain (PF05368, IPR008030) | 3.84 |
| *VDAG_JR2_Chr2g06520a* | Unknown function | - | 2.53 |
| *VDAG_JR2_Chr2g10530a* | Unknown function (potential extracellular, small, C-rich protein, no signal peptide) | - | 3.32 |
| *VDAG_JR2_Chr3g02700a* | Unknown function | Protein of unknown function DUF3405 (PF11885, IPR021822) | 2.25 |
| *VDAG_JR2_Chr3g04160a* | Unknown function (potential effector) | - | 3.12 |
| *VDAG_JR2_Chr3g05470a* | Unknown function | - | 2.08 |
| *VDAG_JR2_Chr3g10760a* | Unknown function | - | 4.09 |
| *VDAG_JR2_Chr4g01040a* | Unknown function | Glyoxalase/Bleomycin resistance protein/Dihydroxybiphenyl dioxygenase (IPR029068) | 4.48 |
| *VDAG_JR2_Chr4g03540a* | Unknown function | WD40 repeat (IPR001680) | 3.21 |
| *VDAG_JR2_Chr4g03560a* | Unknown function (potential extracellular, small, C-rich protein, no signal peptide) | - | 2.83 |
| *VDAG_JR2_Chr4g03830a* | Unknown function (potential membrane protein) | Protein CBG02620 (PTHR35179) | 3.37 |
| *VDAG_JR2_Chr4g07510a* | Unknown function | - | 3.27 |
| *VDAG_JR2_Chr4g10070a* | Unknown function (potentially extracellular) | - | 2.56 |
| *VDAG_JR2_Chr4g10420a* | Unknown function (potentially extracellular) | Stigma-specific protein Stig1 (PF04885, IPR006969) | 2.07 |
| *VDAG_JR2_Chr5g02380a* | Unknown function | - | 2.22 |
| *VDAG_JR2_Chr5g02450a* | Unknown function (potentially extracellular) | - | 2.03 |
| *VDAG_JR2_Chr6g02820a* | Unknown function | - | 6.36 |
| *VDAG_JR2_Chr6g03920a* | Unknown function (potential effector) | - | 3.18 |
| *VDAG_JR2_Chr6g05270a* | Unknown function | Beta-lactamase-related (PF00144, IPR001466) | 2.84 |
| *VDAG_JR2_Chr7g00200a* | Unknown function | - | 2.70 |
| *VDAG_JR2_Chr7g01730a* | Unknown function | - | 2.15 |
| *VDAG_JR2_Chr7g02870a* | Unknown function | - | 2.72 |
| *VDAG_JR2_Chr7g05880a* | Unknown function | - | 2.56 |
| *VDAG_JR2_Chr8g03630a* | Unknown function | Alpha/beta hydrolase fold-1 (PF00561, IPR000073) | 3.42 |
| *VDAG_JR2_Chr8g09780a* | Unknown function | - | 2.21 |

Candidates mentioned in the article are highlighted in yellow; bold: candidate investigated in this study.
