# Supplementary material for: Verticillium dahliae Vta3 promotes ELV1 virulence factor gene expression in xylem sap, but tames Mtf1-mediated late stages of fungus-plant interactions and microsclerotia formation
Source: PLoS Pathog. 2023 Jan 30;19(1):e1011100. doi: 10.1371/journal.ppat.1011100 (PMC9910802; doi:10.1371/journal.ppat.1011100)
Supplement: S9 Table — (DOCX) [file ppat.1011100.s022.docx]

**S9 Table. qPCR primer oligonucleotides used in this study.**

| **Primer name** | **Primer sequence (5’ → 3’)** | **Target gene** | **Reference** |
| --- | --- | --- | --- |
| ***Solanum lycopersicum*** | | | |
| IM106 | GTG GGA TCG GAT TGA TAT CCT | *PR1a*/  *Solyc09g007020.2* | [1] |
| IM107 | CCT AAG CCA CGA TAC CAT GAA | *PR1a*/  *Solyc09g007020.2* | [1] |
| IM108 | CAC AAG CTC AAA ACT CCC CTC A | *PR1*/  *Solyc01g106630.2* | This study |
| IM109 | CCC AGC TCT TTG ATT GGC GTA | *PR1*/  *Solyc01g106630.2* | This study |
| IM110 | GAT CAA GGT GCT TCT AGT TGT GA | *PR3*/  *Solyc04g072000.3* | This study |
| IM111 | TGT GAC ATG AGC GAA GAA AGC AG | *PR3*/  *Solyc04g072000.3* | [2] |
| IM112 | GCC ACT TTC GAG GTA CGC AA | *PR5*/  *Solyc08g080650.2* | [2] |
| IM113 | ATC TTA GTG CCC CTC GGT G | *PR5*/  *Solyc08g080650.2* | This study |
| IM118 | GGA ACT TGA GAA GGA GCC TAA G | *EF1α*/  *Solyc06g005060.3* | [2] |
| IM119 | CAA CAC CAA CAG CAA CAG TCT | *EF1α*/  *Solyc06g005060.3* | [2] |
| IM120 | TCG TGG CCA CTA TAC CAT TG | *αTUB*/  *Solyc08g006890.3* | [3] |
| IM121 | AGT GAC CCA AGA CCT GAA CC | *αTUB*/  *Solyc08g006890.3* | [3] |
| ***Verticillium dahliae*** | | | |
| IM122 | ACG TGC AAC AAG GCC TTC TC | *MTF1*/  *VDAG_JR2_Chr2g08470a* | This study |
| IM123 | GTG GCG CTT CAT GTT GCT G | *MTF1*/  *VDAG_JR2_Chr2g08470a* | This study |
| IM124 | GAA ATG GCG CTG GCA ATG GA | *ELV1*/  *VDAG_JR2_Chr6g05120a* | This study |
| IM125 | CTA AGC CCA ATG CTG CGC | *ELV1*/  *VDAG_JR2_Chr6g05120a* | This study |
| IM126 | GGT CGG CGA TAC AGA ATA AAA TG | *AVE1*/  *VDAG_JR2_Chr5g02170a* | This study |
| IM127 | CAT GGT CGC CTT GTG TGC TGC | *AVE1*/  *VDAG_JR2_Chr5g02170a* | This study |
| IM128 | ACG GGT GGT ATC TCG TGT G | *PRY1*/  *VDAG_JR2_Chr5g03500a* | This study |
| IM129 | TGC AGT CTG GTG CGA TCT CA | *PRY1*/  *VDAG_JR2_Chr5g03500a* | This study |
| IM131 | CGC CAG GAC CAT CAA GTA CT | *424Y*/  *VDAG_JR2_Chr5g00880a* | This study |
| IM132 | GTT GTA GGT GCC GAA GCT C | *424Y*/  *VDAG_JR2_Chr5g00880a* | This study |
| IM133 | GCG GCA CTG ACA TTT GTG C | *CUT11*/  *VDAG_JR2_Chr1g25430a* | This study |
| IM134 | CGT AAA GCG CTG CGC AGC T | *CUT11*/  *VDAG_JR2_Chr1g25430a* | This study |
| IM135 | AGC GGT GAA GTC ACC TCT CTT | *SCP7*/  VDAG_JR2_Chr4g04190a | This study |
| IM136 | CTG GTC GTT GGG GGT CTT TT | *SCP7*/  VDAG_JR2_Chr4g04190a | This study |
| IM137 | AGG CTG TCG TGA CGA AGC A | *ICS1*/  *VDAG_JR2_Chr5g01530a* | This study |
| IM138 | TGA GCC ATG TAC CCG ACG A | *ICS1*/  *VDAG_JR2_Chr5g01530a* | This study |
| IM139 | GGC CGA TGA GGG CGA TTC T | *SCP41*/  VDAG*_JR2_Chr8g01000a* | This study |
| IM140 | CGT CTG CGT GAA GCT CGG T | *SCP41*/  VDAG*_JR2_Chr8g01000a* | This study |
| JST290 | TTC AGA AAA TCC TAA CCT GCA G | *HAC1*/  *VDAG_JR2_Chr2g09780a* | [4] |
| JST291 | ACA CCA ACC GCA ATG CCT | *HAC1*/  *VDAG_JR2_Chr2g09780a* | [4] |
| OLG70 | CAG CGA AAC GCG ATA TGT AG | *5.8S rRNA* | [5] |
| OLG71 | GGC TTG TAG GGG GTT TAG A | *5.8S rRNA* | [5] |
| q*CAP20*-F | TAT CTC CAG AAG GCC GAC AC | *CAP20*/  *VDAG_JR2_Chr6g06890a* | [6] |
| q*CAP20*-R | GCC ATT GCC AAG TTT CTT GT | *CAP20*/  *VDAG_JR2_Chr6g06890a* | [6] |
| q*SNOD1*-F | CCC AAA AGC AGG TCA AGA AG | *CP1*/  *VDAG_JR2_Chr7g00860a* | [6] |
| q*SNOD1*-R | ATG GCG AGG ACA TTG ATG GT | *CP1*/  *VDAG_JR2_Chr7g00860a* | [6] |
| q*SOM1*-F | CCA ACA AGC AGA TGC CTA ATG C | *SOM1*/  *VDAG_JR2_Chr1g09120a* | [6] |
| q*SOM1*-R | CTT GCA GAG CGT GGT TAC TTC C | *SOM1*/  *VDAG_JR2_Chr1g09120a* | [6] |
| q*VDH1*-F | ACG ATT GCT CTG TTT GCT GGA G | *VDH1*/  *VDAG_JR2_Chr2g02500a* | [6] |
| q*VDH1*-R | CCT GGC ACT CTT TGG GGT AGA | *VDH1*/  *VDAG_JR2_Chr2g02500a* | [6] |
| q*VEL1*-F1 | CTA CCC TCG AGG ACA GCA AG | *VEL1*/  *VDAG_JR2_Chr7g04890a* | [6] |
| q*VEL1*-R1 | AGA AAT AGC CAG CCT CAG CA | *VEL1*/  *VDAG_JR2_Chr7g04890a* | [6] |
| q*VTA1*-F | CAC AGG GGC GAG TCT AGG TA | *VTA1*/  *VDAG_JR2_Chr1g15920a* | [6] |
| q*VTA1*-R | CCC GAG GTA CCC GAT CAT AG | *VTA1*/  *VDAG_JR2_Chr1g15920a* | [6] |
| q*VTA2*-F | TAC TCC TTC GTT CCG ATT CCT G | *VTA2*/  *VDAG_JR2_Chr5g09630a* | [7] |
| q*VTA2*-R | GCG CAT TGA GAT GGT TCA GAG T | *VTA2*/  *VDAG_JR2_Chr5g09630a* | [7] |
| q*VTA3*-F | GGA TGG CAA AGT CAA CGT CT | *VTA3*/  *VDAG_JR2_Chr1g07600a* | [6] |
| q*VTA3*-R | CGA ACA GAC CGA ATT GAT CC | *VTA3*/  *VDAG_JR2_Chr1g07600a* | [6] |
| SZ9 | AAC ACC CAG AAC AAG ATG CGC | *H2A*/  *VDAG_JR2_Chr4g01430a* | [4] |
| SZ10 | GCT TGA CCT TGA GAT CCT TG | *H2A*/  *VDAG_JR2_Chr4g01430a* | [4] |
| SZ11 | TGC ATT CTT GGC AAG AGA TGT GTG | *EIF2B*/  *VDAG_JR2_Chr4g00410a* | [8] |
| SZ12 | AGC TTG TTA TCC TTG TCC TCG GT | *EIF2B*/  *VDAG_JR2_Chr4g00410a* | [8] |

**References**

1. Martínez-Medina A, Fernández I, Sánchez-Guzmán MJ, Jung SC, Pascual JA, Pozo MJ. Deciphering the hormonal signaling network behind the systemic resistance induced by *Trichoderma harzianum* in tomato. Front Plant Sci. 2013;4: 206. doi:10.3389/fpls.2013.00206
2. Morales NP. Study of the hydrophobin genes in *Verticillium dahliae* and characterization of the hydrophobin gene *VDH5*. PhD Thesis, The University of Western Ontario, London, Ontario, Canada; 2015. Available: <https://ir.lib.uwo.ca/etd/2818/>
3. Di X, Gomila J, Takken FLW. Involvement of salicylic acid, ethylene and jasmonic acid signalling pathways in the susceptibility of tomato to *Fusarium oxysporum*. Mol Plant Pathol. 2017;18: 1024–1035. doi:10.1111/mpp.12559
4. Starke J, Harting R, Maurus I, Leonard M, Bremenkamp R, Heimel K, et al. Unfolded protein response and scaffold independent pheromone MAP kinase signaling control *Verticillium dahliae* growth, development, and plant pathogenesis. J Fungi (Basel). 2021;7: 305. doi:10.3390/jof7040305
5. Eynck C, Koopmann B, Grunewaldt-Stoecker G, Karlovsky P, von Tiedemann A. Differential interactions of *Verticillium longisporum* and *V. dahliae* with *Brassica napus* detected with molecular and histological techniques. Eur J Plant Pathol. 2007;118: 259–274. doi:10.1007/s10658-007-9144-6
6. Bui T-T, Harting R, Braus-Stromeyer SA, Tran V-T, Leonard M, Höfer A, et al. *Verticillium dahliae* transcription factors Som1 and Vta3 control microsclerotia formation and sequential steps of plant root penetration and colonisation to induce disease. New Phytol. 2019;221: 2138–2159. doi:10.1111/nph.15514
7. Tran V-T, Braus-Stromeyer SA, Kusch H, Reusche M, Kaever A, Kühn A, et al. *Verticillium* transcription activator of adhesion Vta2 suppresses microsclerotia formation and is required for systemic infection of plant roots. New Phytol. 2014;202: 565–581. doi:10.1111/nph.12671
8. Harting R, Höfer A, Tran V-T, Weinhold L-M, Barghahn S, Schlüter R, et al. The Vta1 transcriptional regulator is required for microsclerotia melanization in *Verticillium dahliae*. Fungal Biol. 2020;124: 490–500. doi:10.1016/j.funbio.2020.01.007
