# Supplementary material for: Verticillium dahliae Vta3 promotes ELV1 virulence factor gene expression in xylem sap, but tames Mtf1-mediated late stages of fungus-plant interactions and microsclerotia formation
Source: PLoS Pathog. 2023 Jan 30;19(1):e1011100. doi: 10.1371/journal.ppat.1011100 (PMC9910802; doi:10.1371/journal.ppat.1011100)
Supplement: S10 Table — (DOCX) [file ppat.1011100.s023.docx]

**S10 Table. Proteins significantly enriched in three replicates of Vta3-GFP versus wild-type with LFQ intensities, MS/MS count, unique peptides, sequence coverage and predicted domains.**

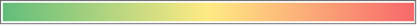


22.35 33.17

Log2(x)LFQ intensity
